# Cooperative reversible assembly in triply interlocked Al_6_L_4_ and Ga_6_L_4_ cages

**DOI:** 10.1039/d5sc05441a

**Published:** 2025-09-24

**Authors:** Ignacio Izquierdo, Laura Martínez-Castro, Gregori Ujaque, Antonio J. Martínez-Martínez

**Affiliations:** a Department of Chemistry and Center for Research in Sustainable Chemistry (CIQSO), Supramolecular Organometallic and Main Group Chemistry Laboratory, University of Huelva Huelva 21007 Spain antonio.martinez@dqcm.uhu.es; b Departamento de Química, Centro de Innovación en Química Avanzada (ORFEO-CINQA), Universitat Autònoma de Barcelona 08193 Cerdanyola Spain

## Abstract

Understanding and controlling the assembly of mechanically interlocked molecules remains a significant challenge. Formation of mechanically interlocked metal–organic cages has, to date, relied exclusively on transition metals due to their predictable coordination geometries and robust bonding. Here, we report, for the first time, the reversible assembly of mechanically interlocked cages based on main-group metals, Al_6_L_4_ and Ga_6_L_4_. Structural and computational analyses reveal helical [2]catenane quadruple-decker cage topologies stabilized by six metal–ligand nodes, bridging μ-OH groups, extensive π-stacking, and directional CH⋯O interactions. Remarkably, simple acid–base cycling triggers fully reversible cage unlocking–recatenation processes in water at room temperature. Unlike transition-metal-mediated cage interlocking, they assemble instantaneously and selectively *via* an unprecedented cooperative main-group interlocking pathway, without detectable monomeric cage intermediates. Thermodynamic analyses reveal metal-dependent switching, involving entropy-driven disassembly coupled to strongly enthalpy-driven reassembly, with the Ga_6_L_4_ cage ∼500-fold more stable than Al_6_L_4_. These findings provide fundamental understanding of new assembly dynamics beyond conventional transition metals.

## Abstract 2

Main-group metals go mechanical: the first triply interlocked main-group metal cages, Al_6_L_4_ and Ga_6_L_4_, assemble cooperatively and reversibly in water at room temperature *via* simple acid–base cycling.

## Graphical abstract

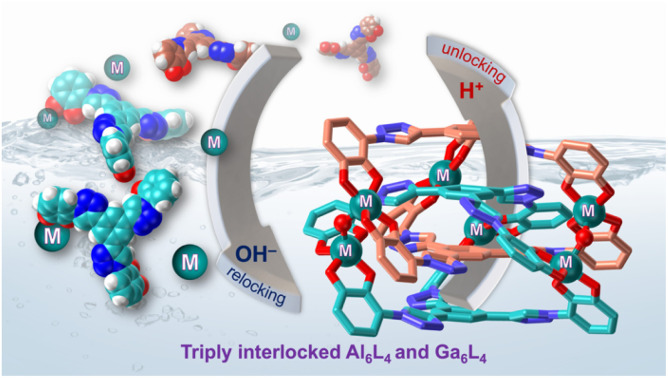

## Introduction

**Fig. 1:**
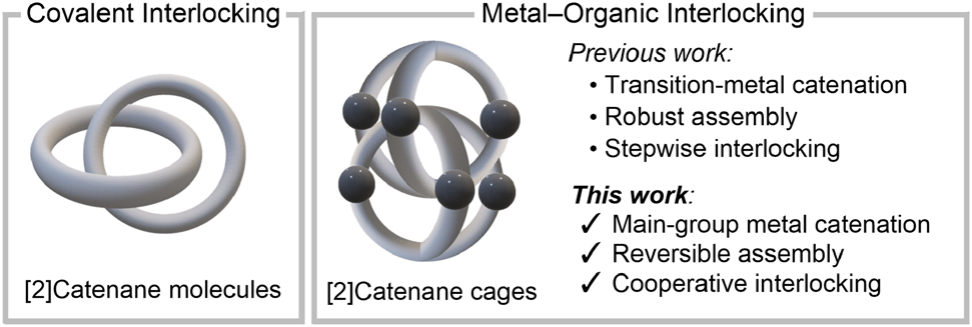
From covalent to metal–organic interlocking: transition-metal *vs.* main-group metal-mediated catenation (this work).

Mechanically interlocked molecules, including catenanes,^1–7^ rotaxanes,^8–13^ clippanes^14^ and molecular knots,^15–23^ enable unique controlled molecular motion, gated transport and stimuli-responsive behaviour.^24,25^ Because their mechanical bonds can only be disrupted by covalent cleavage, these topologically complex architectures underpin emerging technologies from molecular machines,^26,27^ stimulus-gated catalysis^28,29^ to adaptive materials and soft robotics.^30–34^ However, precisely controlling their assembly remains a fundamental challenge. Covalent synthetic routes offer exact connectivity but lack error correction, whereas purely non-covalent methods typically compromise directional precision.^35^ In contrast, metal-directed self-assembly is a powerful tool, combining predictable coordination geometries, thermodynamic error correction, and tunable lability of metal–ligand bonds.^36,37^ These features enable the formation of mechanically interlocked metal–organic architectures (Fig. 1) whose cavity size, shape, and charge can be encoded during self-assembly.

Since Fujita's seminal report of the first mechanically interlocked metal–organic cage, a triply interwoven [2]catenane M_6_L_4_ structure assembled using Pd or Pt,^38^ the field has expanded rapidly. Numerous examples have been developed exclusively using transition metals, including Co,^39^ Cu,^40^ Zn,^39,41^ Ru,^42,43^ Rh,^44–46^ Pd,^47–56^ Ag^57^ and Pt.^58,59^ These cage assemblies exhibit diverse stimulus-responsive functionalities, such as shape-switching,^48,50,54,56^ high-affinity guest binding,^51^ and artificial-muscle-like actuation.^44^ However, reversible mechanical interlocking switching under mild conditions, essential for programmable and responsive supramolecular function, remains rare.^60,61^ Main-group metals offer an orthogonal and unexplored toolkit for switchable mechanical interlocking. Although some examples of discrete monomeric main-group metal–organic cages have been developed,^62–74^ mechanical interlocking involving such metals remains completely unexplored.

Here we introduce first examples of mechanically interlocked metal–organic cages assembled from main-group metals, Al_6_L_4_IC1 and Ga_6_L_4_IC2. Through a simple tritopic ligand, H_6_L, we demonstrate fully reversible acid–base-triggered disassembly and reassembly of their triply interwoven helical [2]catenane topologies in water at room temperature. In contrast to transition-metal cage interlocking assembly, which typically proceeds through dimerization of monomeric cage intermediates, they assemble instantaneously *via* an unprecedented cooperative main-group pathway, without detectable monomeric M_3_L_2_ cage intermediates. Thermodynamic analyses reveal a distinctive metal-dependent switching dynamics, driven by entropy during disassembly and enthalpy during reassembly, with the Ga_6_L_4_ cage exhibiting ∼500-fold higher stability than its Al_6_L_4_ analogue.

## Results and discussion

**Scheme 1:**
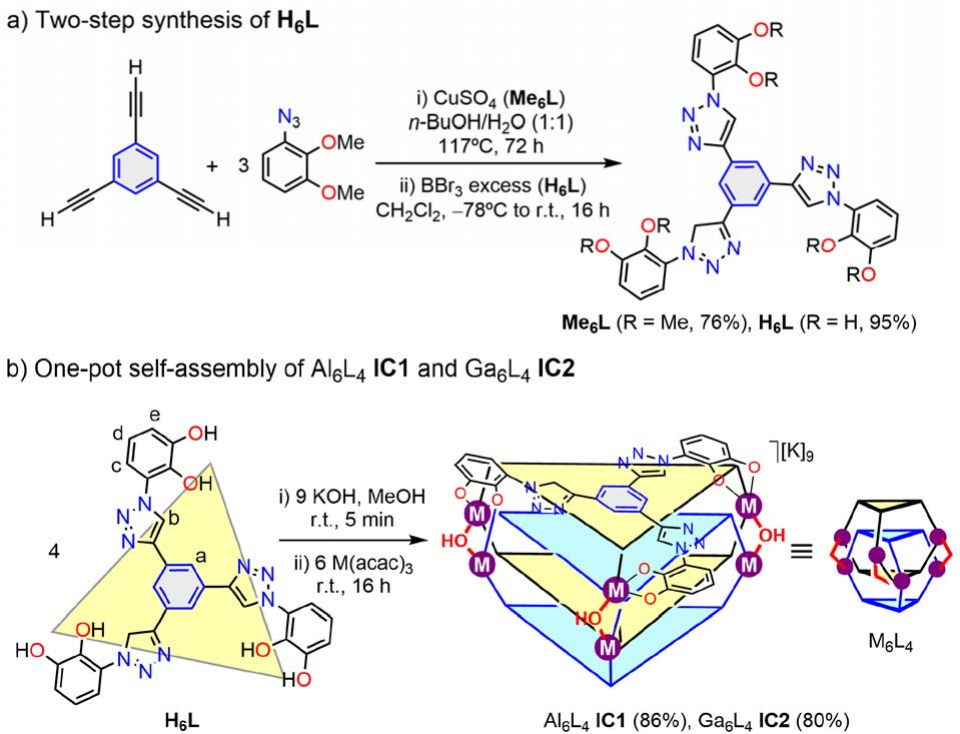
(a) Synthesis of ligands Me_6_L and H_6_L. (b) Self-assembly of triply interlocked quadruple-decker cages Al_6_L_4_IC1 and Ga_6_L_4_IC2. Isolated yields are shown in parenthesis.

The tritopic ligand H_6_L was obtained in two steps (Scheme 1a). A Cu-catalyzed three-fold alkyne–azide “click” reaction (CuAAC)^75,76^ between 1,3,5-triethynylbenzene and 1-azido-2,3-dimethoxybenzene afforded the tris(1,2,3-triazole) precursor Me_6_L (76%). Subsequent demethylation with BBr_3_ gave H_6_L quantitatively (95%). The terminal catechol groups offer robust chelation for oxophilic M^3+^ ions, while the rigid *C*_3v_ phenylene C_6_H_3_ core pre-arranges three metal binding sites within a trigonal array. Furthermore, the bridging triazole units add conformational adaptability and promote π⋯π/CH⋯π stackings. Self-assembly of H_6_L with Al(acac)_3_ or Ga(acac)_3_ (acac = acetylacetonate), and KOH in a ratio 4 : 6 : 9 in MeOH, selectively produced the triply interlocked cages Al_6_L_4_IC1 and Ga_6_L_4_IC2 within 16 hours at room temperature (Scheme 1b). This click-demethylation-assembly sequence provided IC1 and IC2 in 86% and 80% yield, respectively, after simple trituration with diethyl ether. Notably, IC1 and IC2 represent the first examples of mechanically interlocked metal–organic cages constructed exclusively from main-group metals.^60^

**Fig. 2:**
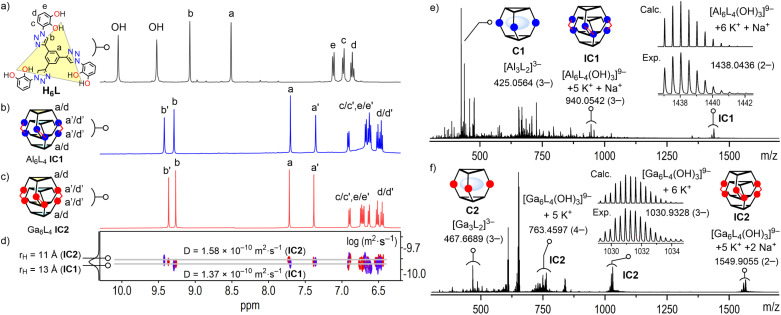
(a) ^1^H NMR spectra (400 MHz, 298 K) of ligand H_6_L in DMSO-*d*_6_, (b) cage Al_6_L_4_IC1 (blue) and (c) Ga_6_L_4_IC2 (red) in D_2_O; duplicated ligand environments are labelled with a/d and a′/d′. (d) Overlay of DOSY NMR (400 MHz, 298 K) for IC1 and IC2 in D_2_O. (e) ESI-TOF HR mass spectra (negative mode) of IC1 and (f) IC2, showing selected peaks for interlocked cages [M_6_L_4_]^*n*−^ and [M_3_L_2_]^*n*−^C1 and C2 units.

Disappearance of the catechol OH signals of H_6_L (*δ* 10.05 and 9.52 ppm) in the ^1^H NMR spectra of IC1 and IC2 confirms metal–catecholate coordination (Fig. 2a–d). Each interlocked cage displays two sets of sharp resonances in D_2_O at 298 K, reflecting two chemically inequivalent ligand environments. This spectral duplication evidences the mechanical interpenetration of two cage monomeric units, Al_3_L_2_C1 or Ga_3_L_2_C2, within each dimeric assembly. The triazole protons H^b^/H^b^′ shift downfield (IC1: *δ* 9.28/9.42 ppm; IC2: *δ* 9.26/9.36 ppm) relative to H_6_L (*δ* 9.08 ppm), consistent with a peripheral cage location. In contrast, the phenylene C_6_H_3_ core protons H^a^/H^a^′ are strongly shielded (IC1: *δ* 7.70/7.36 ppm; IC2: *δ* 7.71/7.38 ppm; *vs.*H_6_L: *δ* 8.52) owing to the π-stacking within the cage interior. ^13^C NMR data in D_2_O also shows duplicated sets at 298 K, confirming ligand interpenetration (Fig. S18 and S35, SI).

Complementary ^1^H NMR in CD_3_OD shows that the duplicated ligand resonances visible in D_2_O coalesce into single broadened sets at 298 K. H^b^/H^b^′ appear as averaged and broad singlets at *δ* 9.12 (IC1) and 9.14 (IC2) ppm, and H^a^/H^a^′ at *δ* 8.43 (IC1) and 8.34 (IC2) ppm (Fig. S16 and S33, SI). This solvent-dependent modulation reflects the weaker hydrophobic/solvophobic driving force in CD_3_OD relative to D_2_O, which attenuates π-stacking in IC1 and IC2, thereby reducing inner/outer chemical-shift differences (Δ*δ*) and averaging them on the NMR timescale at 298 K. Variable-temperature ^1^H NMR in CD_3_OD (318–238 K) progressively resolves these resonances as exchange slows upon cooling to 238 K. At 238 K, the ligand resonances desymmetrize, H^b^/H^b^′ resolve into five (IC1) and four (IC2) partially overlapping broad singlets spanning *δ* 9.04–9.31 ppm and *δ* 9.31–9.60 ppm, respectively. Likewise, H^a^/H^a^′ split into five (IC1) and three (IC2) overlapping broad singlets *δ* 8.38–8.80 ppm and *δ* 8.29–8.37 ppm, respectively; with the catecholate H^c^/H^d^/H^e^ showing analogous behavior (Fig. S55 and S56, SI). Warming up to 298 K restores the initial state. These low-temperature spectra are consistent with freezing of the interlocked topologies IC1 and IC2, where four ligands are arranged in a non-equivalent fashion (*vide infra*).

High-resolution ESI-TOF MS (negative mode) confirmed the M_6_L_4_ formulation for IC1 and IC2. Each spectrum displays isotopically resolved series of peaks for intact anions [Al_6_L_4_]^*n*−^ (for IC1) and [Ga_6_L_4_]^*n*−^ (for IC2) in charge states 4−, 3−, and 2−, detected as Na^+^/K^+^ adducts drawn from the ionization medium (Fig. 2e and f). A second family of peaks reveals that each cage retains three hydroxide OH^−^ groups. IC1 gives representative peaks at *m*/*z* 940.0542 (3−) and 1438.0436 (2−) for [Al_6_L_4_(OH)_3_ + *x*A]^*n*−^, whereas IC2 furnishes an analogous series at *m*/*z* 763.4597 (4−), 1030.9328 (3−) and 1549.9055 (2−) for [Ga_6_L_4_(OH)_3_ + *x*A]^*n*−^ (A = Na^+^, K^+^). In addition, in-source collisional activation cleaves each interlocked assembly into units [Al_3_L_2_]^3−^C1 (*m*/*z* 425.0564) and [Ga_3_L_2_]^3−^C2 (*m*/*z* 467.6689), evidencing mechanical unlocking of IC1 and IC2 during ionization.

**Fig. 3:**
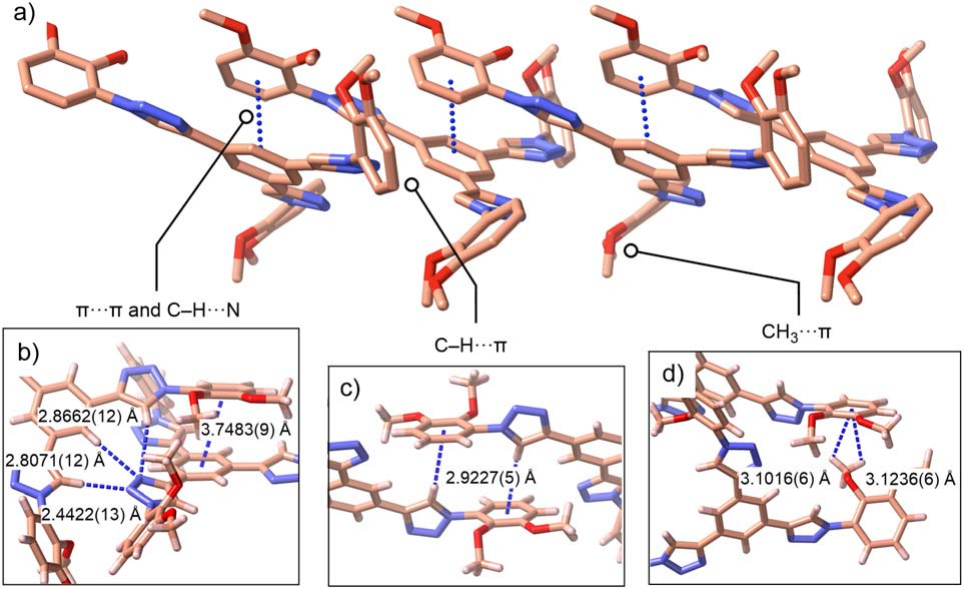
Crystal packing of Me_6_L highlighting key non-covalent interactions: (a) side-on view of a slipped stack showing π-overlap, (b) close-up of π⋯π and C–H⋯N contacts, (c) representative CH⋯π contacts and (d) Me⋯π interactions.

Single crystals of Me_6_L suitable for X-ray diffraction were obtained by slow evaporation of a concentrated EtOAc solution (50 mM, 0.5 mL) at room temperature over 24 h. The crystal structure of Me_6_L reveals a planar 1,3,5-phenylene C_6_H_3_ core flanked by three peripheral 1,2,3-triazoles, promoting extended ligand conjugation (Fig. 3a). The catechol termini are pre-organized at distances of ∼15.1 Å, ideal for chelating remote M^3+^ nodes. Packing is governed by π⋯π contacts (centroid⋯centroid 3.7483(9) Å) supplemented by C–H⋯π/Me⋯π/C–H⋯N interactions (Fig. 3b and c), underscoring the intrinsic propensity of this ligand framework for stacking. DOSY NMR studies corroborate a significant aggregation of both Me_6_L and H_6_L in solution. The measured diffusion coefficients (*D*) of 3.10 × 10^−10^ m^2^ s^−1^ for Me_6_L and 2.60 × 10^−10^ m^2^ s^−1^ for H_6_L correspond to Stokes–Einstein hydrodynamic radii (*r*_H_) of 9 and 11 Å (Fig. S57 and S58, SI), respectively, roughly twice the estimated monomer size (6 Å) and consistent with predominant dimer aggregation. This pre-organization is expected to reduce the entropic cost of cage assembly using H_6_L.

**Fig. 4:**
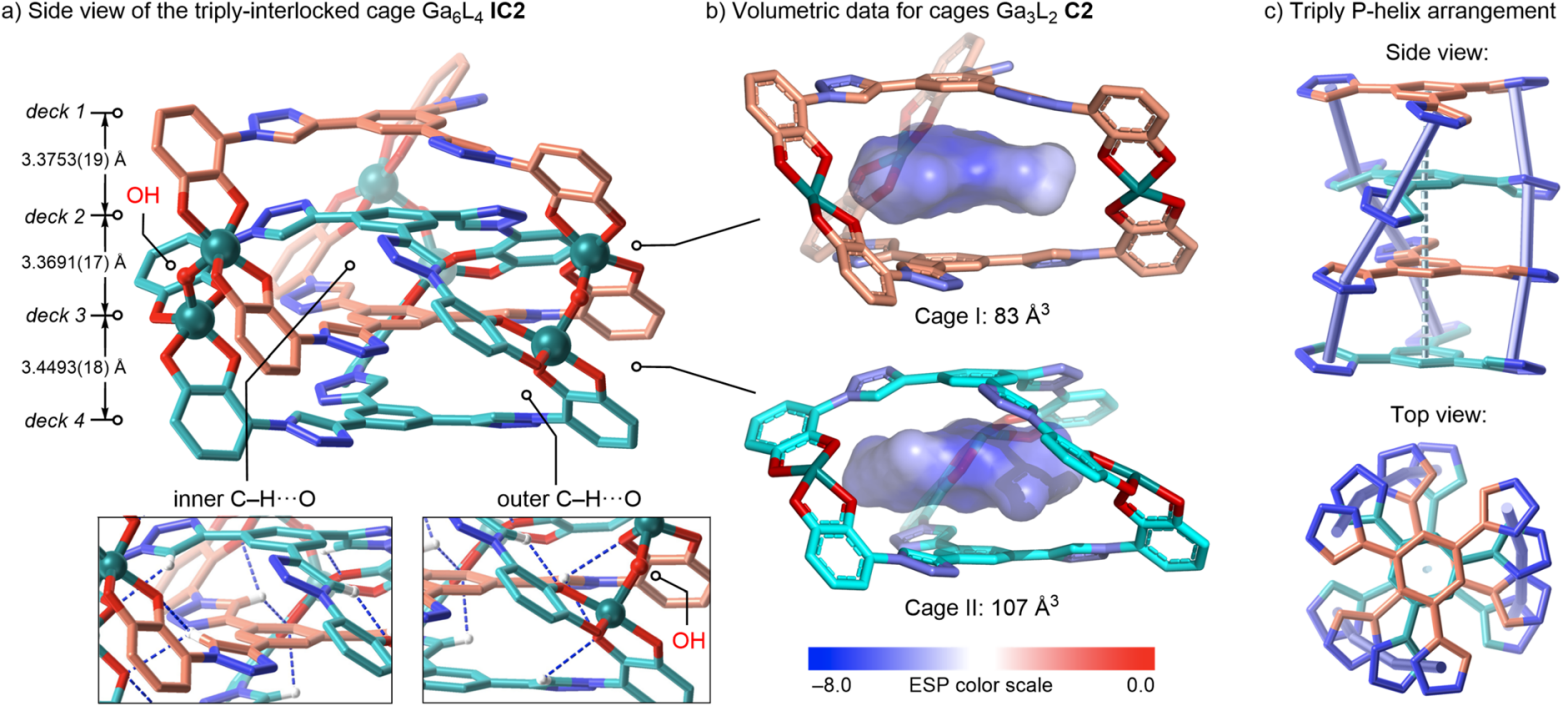
Crystal structure of the triply interlocked cage Ga_6_L_4_IC2 (anionic form [Ga_6_L_4_(OH)_3_]^9−^). (a) Side view of the quadruple-decker assembly; μ-OH bridges and Ga atoms are shown ball-and-sticks, and key CH⋯O contacts as dashed lines. (b) Isolated Ga_3_L_2_C2 units (cage I and cage II) rendered with internal voids as ESP isosurfaces calculated using CageCavityCalc-C3 (grid size 0.7 Å, eem charge model). (c) P-Helical arrangement of the four ligand decks (side and top). H atoms (except those in CH⋯O contacts), K^+^ ions, and solvent are omitted; C beige/cyan, N blue, O red, Ga teal.

Single crystals of the cage Ga_6_L_4_IC2 suitable for X-ray diffraction, as its potassium salt form K_9_[Ga_6_L_4_(OH)_3_], were grown by vapor diffusion of acetone (2 mL) into a 1 : 1 H_2_O/MeOH solution of the cage (10 mM, 0.5 mL) at room temperature over one week. These crystals confirm the anticipated triply interlocked [2]catenane topology (Fig. 4). Two mechanically threaded monomeric units Ga_3_L_2_C2 (cage I and cage II) are connected by six Ga–catecholate chelate nodes (Ga–O 1.8926(18)–1.9544(19) Å). At each of the three crossing nodes a μ-OH bridge spans a Ga_2_ unit (Ga–OH 1.890(2)–1.928(2) Å), rendering every Ga^3+^ center five-coordinate within the anionic [Ga_6_L_4_(OH)_3_]^9−^ framework. Each Ga_3_L_2_C2 cage encloses a prolate cavity with 6.8180(18) and 6.7422(19) Å inter-deck spacings and voids of 83 and 107 Å^3^ (calculated using CageCavityCalc-C3),^77^ ideally sized to host the tris(triazole)phenylene hub of its interpenetrating Ga_3_L_2_ partner (Fig. 4b). ESP mapping shows a uniformly negative potential inside each cavity, reflecting the π-rich ligand walls in each formal Ga_3_L_2_C2 unit, suggesting that dispersion, π-stacking and Ga–O coordination likely drive interlocking.

Additional NCI analyses of DFT-optimized (B3LYP-D3/6-31g*) structures of IC1 and IC2 provided deeper insights into the non-covalent forces underpinning these assemblies. The presence of diffuse attractive isosurfaces highlights extensive π-stacking between ligand layers in both interlocked assemblies IC1 and IC2 (Fig. S97 and S98, SI). The distinct interaction maps, despite similar, suggest greater stability of the cage IC2 compared to IC1 through enhanced π-stacking (slightly larger and greener surfaces).

Four ligand decks form an eclipsed π-stack (inter-deck phenylene centroid⋯centroid: 3.3691(17), 3.3753(19) and 3.4493(18) Å). These decks are successively rotated by 17.28(3)–29.16(3)°, generating P/M helices that crystallize as a racemate in *P*1̄ space group (P-helix is shown, Fig. 4c). An additional π-staircase array of contacts between triazoles (3.5120(19)–4.044(2) Å), supported by directional internal CH⋯O interactions (2.266(2)–2.782(2) Å), further consolidate this quadruple-decker. When interlocked, three tris(triazole)-phenylene cores remain planar (Fig. 4a: decks 1, 3 and 4), whereas one bends (deck 2) to accommodate the interlocked array IC2, underscoring the adaptive flexibility of the ligand L^6−^ framework. In addition, these discrete anionic interlocked [Ga_6_L_4_(OH)_3_]^9−^ cages pack into a three-dimensional lattice *via* outer-sphere K^+^ bridges and solvent channels (Fig. S95, SI).

The four ligands are crystallographically non-equivalent in IC2, partitioning into two inner and two outer decks. This solid-state asymmetry rationalizes the inner/outer twofold NMR splitting observed D_2_O solution and the further desymmetrisation observed in CD_3_OD at 238 K. Moreover, ^1^H,^1^H-NOESY cross-peaks between inner phenylene H^a^′ and outer triazole H^b^ confirm face-edge proximity required for the quadruple-decker helix (Fig. S22 and S39, SI). Additional inter-deck H^a^/H^a^′ NOESY contacts between neighboring phenylene C_6_H_3_ cores confirm retention of the π-stacking motif of IC1 and IC2 in solution. DOSY experiments further supports intact cage architectures in D_2_O solution (IC1: *D* = 1.37 × 10^−10^ m^2^ s^−1^; IC2: *D* = 1.58 × 10^−10^ m^2^ s^−1^, both in D_2_O, Fig. 2d), yielding *r*_H_ of 13 and 11 Å, respectively, demonstrating retention of the interlocked topologies in water (estimated crystallographic radii of 13 Å for IC2, see SI). Additional DOSY results in CD_3_OD solution (IC1: *D* = 2.75 × 10^−10^ m^2^ s^−1^, *r*_H_ = 13 Å; IC2: *D* = 2.90 × 10^−10^ m^2^ s^−1^, *r*_H_ = 12 Å) closely matching the structural cage dimensions, confirming intact interlocked topologies in both D_2_O and CD_3_OD.

**Fig. 5:**
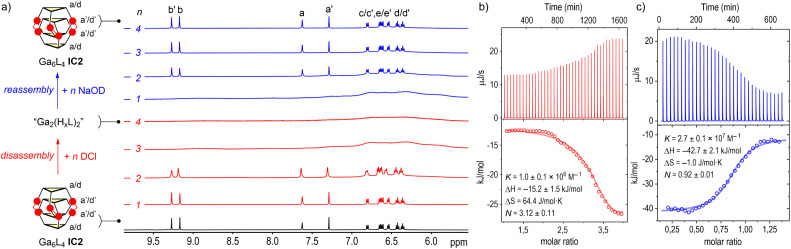
Reversible acid–base switching of the interlocked cage Ga_6_L_4_IC2: (a) ^1^H NMR (500 MHz, 298 K, 4 mM) stack for Ga_6_L_4_IC2 during titration with DCl (1–4 equiv., red) and back-titration with NaOD (1–4 equiv., blue) in D_2_O (identical spectra are obtained in the 1–5 mM range). ITC traces: (b) disassembly (1–4 equiv. of DCl) and (c) reassembly (0–1.5 equiv. of NaOD) of IC2 in D_2_O (1 mM) at 298 K.

Motivated by the unique topological features and the presence of stabilizing μ-OH bridges, we examined the response of the cages IC1 and IC2 to protonation. Titration of solutions of IC1 and IC2 with incremental additions of deutero-hydrochloric acid (DCl) in D_2_O were monitored by ^1^H NMR (Fig. 5). Cage IC1 underwent rapid structural disruption upon addition of one equivalent of acid as evidenced by progressive disappearance and broadening of the cage resonances after four equivalents (Fig. S64, SI). By contrast, cage IC2 remains intact until the third equivalent of acid, underscoring its higher kinetic and thermodynamic robustness (Fig. 5a). Remarkably, subsequent neutralization with sodium deuteroxide (NaOD, 1–4 equiv.) fully restores the diagnostic spectra of each interlocked cage IC1 and IC2. While IC1 recatenates after 3–4 equivalents of base, IC2 readily interlocks after one equivalent. Identical spectral changes were obtained across the window 1–5 mM, the highest range permitted by the solubility of IC1 and IC2, demonstrating a fully reversible unlocking/recatenation process. The absence of resonances attributable to monomeric M_3_L_2_ cages C1 or C2 further supports a highly cooperative and effectively direct assembly pathway under these conditions.

Lyophilization of the acidified solutions allowed us to analyze the unlocked intermediates resulting from disassembling both cages, IC1 and IC2. ESI-TOF mass analysis reveal dominant ions at *m*/*z* 626.1121 (2−) and 669.0500 (2−) corresponding to partially protonated species [Al_2_(H_*x*_L)_2_]^*n*−^ and [Ga_2_(H_*x*_L)_2_]^*n*−^ (Fig. S74 and S85, SI). The absence of higher-mass ions suggests that protonation lowers the net charge, favoring H-bonded “M_2_(H_*x*_L)_2_” species that exchange rapidly in D_2_O solution and thus broaden the ^1^H NMR signals during disassembly. Notably, under these partially acidified conditions (4 equiv.), the dominant [M_2_(H_*x*_L)_2_]^*n*−^ ions do not contain OH^−^ groups, in contrast to the intact cage [M_6_L_4_(OH)_3_]^*n*−^ ions, where μ-OH bridging is implicit. This indicates protonation/disruption of the μ-OH bridges during unlocking; conversely, reappearance of the IC1 and IC2 resonances upon neutralization with base correlates with re-formation of these bridges. IR spectroscopy showed a broad *ν*(OH) at 3500 cm^−1^ and ^1^H NMR in DMSO-*d*_6_ confirming partial catechol reprotonation (Fig. S81–S84, SI). Together, these observations indicate that full deprotonation of H_6_L and intact μ-OH bridges are pre-requisites for cage assembly.

While controlled addition of acid (1–4 equiv.) resulted in partial protonated “M_2_(H_*x*_L)_2_” species, addition of a large excess of acid (30 equiv.) fully protonated the system, regenerating back the neutral ligand H_6_L. To map the direct interlocking pathway, we conducted *in situ* NMR monitoring starting from the fully protonated ligand H_6_L. Titration of H_6_L with incremental additions of NaOD (1–6 equiv.) in D_2_O generates the hexaanionic ligand species, L^6−^, in its sodium form Na_6_L (Fig. S68–S70, SI). DOSY NMR analysis reveals a significant dimeric pre-organization of Na_6_L in solution (*D* = 2.08 × 10^−10^ m^2^ s^−1^, *r*_H_ = 9 Å), akin to Me_6_L and H_6_L. Addition of two equivalents of AlCl_3_ and GaCl_3_ as water soluble sources of Al^3+^ and Ga^3+^ ions, immediately produced the duplicated ligand ^1^H NMR resonances of IC1 and IC2. Although partial replacement of the bridging μ-OH groups for μ-Cl and outer-sphere exchange of K^+^ for Na^+^ cannot be excluded under these conditions, the formation of IC1 and IC2 is essentially instantaneous (<2 min), underscoring the high kinetic facility of this unusual main-group interlocking process. The rapid assembly is consistent with cooperative metal–catecholate chelation together with a hydrophobic/solvophobic contribution that further strengthens π-stacking and releases structured solvent molecules and counterions from the cavity regions during the assembly of IC1 and IC2. This essentially direct main-group interlocking pathway, occurring without detectable formation of monomeric cage intermediates M_3_L_2_C1 or C2, contrasts markedly with the established monomer-to-dimer interlocking pathways typical for transition-metal-mediated cage assemblies.^41,48,50,52,54,55,78^

**Fig. 6:**
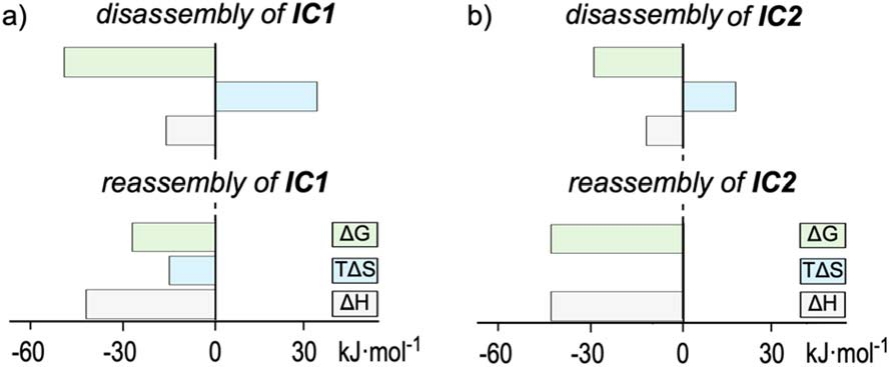
Thermodynamic ITC data (Δ*G*, *T*Δ*S* and Δ*H*) for acid-induced disassembly and base-induced reassembly: (a) Al_6_L_4_IC1 and (b) Ga_6_L_4_IC2.

To quantify the thermodynamic driving forces underlying the reversible assembly of IC1 and IC2, isothermal titration calorimetry (ITC) was carried out under the same acid–base NMR cycling regimes at 1 mM concentrations (Fig. 5b, c and S72, SI). The ITC isotherms captured single cooperative transition equilibria for both the disassembly and reassembly of IC1 and IC2, therefore, data were fitted to one-set-of-sites models (independent-sites/Wiseman). Hence, the overall macroscopic equilibria returned N (acid/base titrant equivalents per cage at the transition) and apparent macroscopic equilibrium constants *K* (M^−1^) for the overall disassembly (cage + *n*H^+^ ⇄ disassembled state) and reassembly (disassembled state + *n*OH^−^ ⇄ cage) modelled steps at the specific transition N equivalents. Acid-induced disassembly is entropy-driven (*T*Δ*S* > 0, Fig. 6) for both cages IC1 (*T*Δ*S* = 32.9 kJ mol^−1^) and IC2 (*T*Δ*S* = 19.1 kJ mol^−1^), consistent with the gain in translational/rotational and conformational freedom upon releasing ligands and metal ions. The modest exothermicity (IC1: Δ*H* = −16.0 kJ mol^−1^; IC2: Δ*H* = −15.2 kJ mol^−1^) is attributed to protonation of μ-OH bridges and catecholate groups. Overall, unlocking is easier for IC1 (Δ*G* = −48.9 kJ mol^−1^) than for IC2 (Δ*G* = −34.3 kJ mol^−1^), consistent with the higher intrinsic stability of the Ga_6_L_4_ cage IC2. Consistently, the ITC stoichiometries indicate *N* = 1.06 ± 0.03 acid equivalent per cage for IC1 and *N* = 3.12 ± 0.11 for IC2 during disassembly, matching the macroscopic equivalence points seen by ^1^H NMR titrations. Reassembly on base addition is strongly enthalpy-driven (IC1: Δ*H* = −42.1 kJ mol^−1^; IC2: Δ*H* = −42.7 kJ mol^−1^). This reflects the formation of twelve M–O chelate bonds and three μ-OH bridges, reinforced by dense π⋯π and C–H⋯O contacts within the quadruple-decker stacks, providing a large exothermic gain that outweighs the desolvation costs. The reassembly entropy terms diverge. IC1 pays a small entropic penalty (*T*Δ*S* = −15.3 kJ mol^−1^), whereas IC2 incurs essentially none (*T*Δ*S* = −0.3 kJ mol^−1^). This reflects the balance between ordering penalties and the favorable hydrophobic/solvophobic release of structured solvent molecules and counterions from catecholate solvation shells and π-rich cavities during interlocking. We ascribe the minimal entropic term in IC2 to a more extensive π-stacking and stronger Ga–O chelation, which together favor a more pre-organized unlocked ensemble and better compensates ordering in water compared to IC1. The resulting reassembly free energies (IC1: Δ*G* = −26.8 kJ mol^−1^; IC2: Δ*G* = −42.4 kJ mol^−1^) confirm the greater thermodynamic stability of the Ga_6_L_4_ cage IC2. In line with this, recatenation proceeds with *N* = 3.62 ± 0.04 base equivalents per cage for IC1*vs. N* = 0.92 ± 0.01 for IC2, again consistent with the ^1^H NMR titrations. The obtained thermodynamic magnitudes are consistent with other metal–organic cages, where guest capture/assembly is generally exothermic and enthalpy-driven (typically Δ*H* ∼ −20 to −60 kJ mol^−1^; *T*Δ*S* ∼ −15 to 25 kJ mol^−1^), while release/exchange commonly shows favorable entropy (*T*Δ*S* > 10 kJ mol^−1^).^79–81^

At 298 K, the recatenation free energies are Δ*G* = −26.8 kJ mol^−1^ (*K* = 5.1 × 10^4^ M^−1^) for IC1 and Δ*G* = −42.4 kJ mol^−1^ (*K* = 2.7 × 10^7^ M^−1^) for IC2. For unlocking, Δ*G* is −48.9 kJ mol^−1^ for IC1 (*K* = 3.7 × 10^8^ M^−1^) and −34.3 kJ mol^−1^ (*K* = 1.0 × 10^6^ M^−1^) for IC2. The resulting ΔΔ*G*(IC2–IC1) = −15.6 kJ mol^−1^ for recatenation, corresponds to a ∼500-fold greater thermodynamic preference for the Ga_6_L_4_ cage IC2 over the Al_6_L_4_ cage IC1. This metal dependence is consistent with the generally higher affinity of Ga–O chelation relative to Al–O (*e.g.*, phenolate chelates exhibit log *K* ∼ 31 for Ga *vs.* 22 for Al in water),^82^ reinforcing that stronger Ga–catecholate binding together with more extensive π-stacking, seen by NCI, stabilizes the triply interlocked Ga_6_L_4_IC2 topology.

## Conclusions

We have demonstrated an unprecedented reversible assembly of the first examples of mechanically interlocked metal–organic cages involving main-group metals, Al_6_L_4_ and Ga_6_L_4_. A simple tritopic ligand, H_6_L, directly folds each metal into helical, triply interwoven [2]catenane quadruple-decker topologies. Structural and computational studies confirm cage stabilization *via* six metal–ligand nodes, bridging μ-OH groups, extensive π-stacking and directional CH⋯O interactions. Remarkably, simple acid–base cycling induces fully reversible cage disassembly–reassembly in water at room temperature. Unlike transition-metal-mediated cage interlocking, which typically assemble through detectable monomeric cages, this main-group metal assembly proceeds rapidly through an unusual cooperative pathway without observable monomeric M_3_L_2_ cage intermediates. Thermodynamic analyses reveal a metal-dependent switching behavior through entropy-driven unlocking coupled with strongly enthalpy-driven recatenation. The Ga_6_L_4_ cage exhibits ∼500-fold higher stability than Al_6_L_4_ (Δ*G* = −42.4 *vs.* −26.8 kJ mol^−1^), reflecting enhanced Ga–ligand affinity and π-stacking. These results provide new fundamental insights into main-group metal-driven interlocking, thereby opening opportunities for novel stimuli-responsive supramolecular materials beyond conventional transition-metal systems.

## Author contributions

I. I. conducted all experiments, synthesis, characterisation, NMR and ITC assembly studies. L. M. C. and G. U. performed electronic structure calculations of molecular models. A. J. M. M.: conceived the idea, project supervision, wrote the manuscript and conducted X-ray crystallography. All authors provided comments and approved the final version of the manuscript.

## Conflicts of interest

There are no conflicts to declare.

## Supplementary Material

SC-OLF-D5SC05441A-s001

SC-OLF-D5SC05441A-s002

## Data Availability

The datasets supporting this article have been uploaded as part of the Supplementary Information (SI). Supplementary information: experimental procedures, characterisation data, selected figures, crystallographic and computational details. See DOI: https://doi.org/10.1039/d5sc05441a. CCDC 2467878 and 2467879 contain the supplementary crystallographic data for this paper.^83*a*,*b*^
