# Supplementary material for: Cooperative reversible assembly in triply interlocked Al6L4 and Ga6L4 cages
Source: Chem Sci. 2025 Sep 24;16(43):20406–13. doi: 10.1039/d5sc05441a (PMC12495313; doi:10.1039/d5sc05441a)
Supplement: SC-016-D5SC05441A-s001 [file SC-016-D5SC05441A-s001.pdf]

Supplementary Information (SI) for Chemical Science.
This journal is © The Royal Society of Chemistry 2025

Electronic Supporting Information

Cooperative Reversible Assembly in Triply Interlocked Al_6L_4 and Ga_6L_4 Cages

Ignacio Izquierdo,[a] Laura Martínez-Castro,[b] Gregori Ujaque,[b] Antonio J. Martínez-Martínez[a,*]

[a] *Department of Chemistry and Supramolecular Organometallic and Main Group Chemistry Laboratory, Center for Research in Sustainable Chemistry (CIQSO), University of Huelva, Huelva 21007, Spain*

[b] *Departamento de Química and Centro de Innovación en Química Avanzada (ORFEO-CINQA), Universitat Autònoma de Barcelona, 08193 Cerdanyola, Spain*

* *E-mail*: antonio.martinez@dqcm.uhu.es

Table of Contents

S1 General Information ... **3**

S2 Ligand syntheses and characterization ... **4**

S2.1 Synthesis of Me_6L ... 4

S2.2 Synthesis of H_6L ... 8

S3 Self-assembly and characterization of coordination cages ... **12**

S3.1 Self-assembly of IC1 ... 12

S3.2 Self-assembly of IC2 ... 21

S4 Variable Temperature (VT) NMR Spectroscopic Studies ... **34**

S4.1 ^{1}H VT NMR Spectroscopic Study of IC1 in CD_3OD (238–318 K) ... 34

S4.2 ^{1}H VT NMR Spectroscopic Study of IC2 in CD_3OD (238–318 K) ... 35

S5 Diffusion Ordered NMR Spectroscopic (DOSY) studies ... **35**

S5.1 DOSY NMR general information ... 35

S5.2 Study of solvent and aggregation effects ... 36

S5.3 DOSY NMR studies of ligands ... 37

S5.4 DOSY NMR studies of coordination cages ... 39

S6 NMR studies of the disassembly–reassembly of IC1 and IC2 ... **44**

S6.1 In-situ NMR titration monitoring of disassembly–reassembly of IC1 with DCl–NaOD ... 44

S6.2 In-situ NMR titration monitoring of disassembly–reassembly of IC2 with DCl–NaOD ... 45

S6.3 In-situ NMR monitoring of assembly of IC1 via titration with $AlCl_3$ ... 47

S6.4 In-situ NMR monitoring of assembly of IC2 via titration with $GaCl_3$ ... 48

S6.5 In-situ NMR monitoring of the deprotonation–reprotonation of H_6L with NaOD–DCl ... 49

S7 Calorimetric studies of the disassembly–reassembly of IC1 and IC2 ... **52**

S7.1 ITC general procedures ... 52

S7.2 ITC study of the disassembly–reassembly of IC1 ... 53

S7.3 ITC study of the dissasembly–reassembly of IC2 ... 54

S8 Lyophilization studies of the disassembly of IC1 and IC2 ... **56**

S8.1 Lyophilization and characterization of disassembled species for IC1 ... 56

S8.2 Lyophilization and characterization of disassembled species for IC2 ... 60

S9 X-ray crystallography ... **65**

S9.1 Single-crystal X-ray structural determination details ... 65

S9.2 Selected crystallographic and refinement data ... 66

S9.3 Supplementary X-ray figures and metrics ... 68

S9.4 Cavity-volume and ESP calculations for IC2 ... 75

S10 Computational details ... **76**

S10.1 NCI Analyses of IC1 and IC2 ... 76

S10.2 Cartesian coordinates of IC1 and IC2 ... 78

S11 References ... **83**

S1 General Information

Unless otherwise stated, all experiments were carried out under air atmosphere except for the synthesis and handling of the supramolecular coordination compounds **IC1** and **IC2**, which were carried out under an inert nitrogen atmosphere using either a glove box (MBraun, LabMaster SP) or standard Schlenk techniques to control their hydration levels. Methanol was dried over Mg for two hours and then distilled under a nitrogen atmosphere, subsequently, it was deoxygenated with nitrogen gas and stored under an atmosphere of nitrogen over molecular sieves (3 Å). Dichloromethane (DCM) was obtained from an SPS system and stored under an atmosphere of nitrogen gas. Deuterated chloroform ($CDCl_3$), deuterium oxide (D_2O), deuterated dimethyl sulfoxide (DMSO-d_6) and deuterated dimethyl formamide (DMF-d_7) were purchased from Cambridge Isotopes or Merck. 1,3,5-Tribromobenzene, trimethyl silyl acetylene (TMSA), CuI, $Pd(OAc)_2$, PPh_3, iPr_2NH, KOH, NaN_3, 2,3-dimethoxyphenylboronic acid, $CuSO_4$, L-ascorbic acid, *n*-BuOH, BBr_3, $Ga(acac)_3$ and $Al(acac)_3$ (acac = acetyl acetonate) were purchased from Merck and used as received. 1,3,5-Triethynylbenzene[1] and 2,3-dimethoxyphenyl azide[2] were prepared according to reported procedures. All other chemicals were purchased from commercial suppliers and used without further purification unless otherwise specified.

NMR experiments were conducted using 5 mm NMR tubes. Multinuclear NMR spectra were collected on either a Bruker AVIIIHD 400 nanobay (equipped with a 9.4 tesla magnet) or Bruker AVIII 500 (equipped with an 11.7 tesla magnet and a Prodigy cryoprobe) spectrometer. Deuterated solvents (D_2O, $CDCl_3$) were purchased from Cambridge Isotope Laboratories Inc. and used as received without any further purification. Standard NMR solutions of deuterium chloride DCl (7.6N in D_2O) and sodium deuteroxide NaOD (40% weight in D_2O) were purchased from Eurisotop and used as received without further purification. Assignments of NMR resonance signals in the ^{1}H and $^{13}C\{^{1}H\}$ NMR spectra were performed with the assistance of two-dimensional NMR experiments ($^{1}H,^{1}H$-COSY, $^{1}H,^{13}C$-HSQC, $^{1}H,^{13}C$-HMBC). For NOESY (phase sensitive) experiments, standard pulse sequences were used, consisting of 256 transients with 32 scans in the f1 domain using an optimized 300 ms mixing time and 2 sec relaxation delay. Chemical shifts (δ) are reported in parts per million (ppm) and referenced to the residual solvent resonances. Coupling constants (J) are given in Hertz (Hz). Signal multiplicities are abbreviated as follows: s (singlet), d (doublet), sept (septet), m (multiplet) and br (broad). RT (25ºC).

Electrospray ionization high resolution mass spectrometry (ESI-HRMS) was carried out using a Bruker Compact QToF. Typical acquisition parameters were used (sample rate flow: 3 µL min^{-1}, nebulizer gas pressure: 0.3 bar, drying gas: nitrogen at 200 K flowing at 3 L min^{-1}, capillary voltage: 3.5 kV, exit voltage: 0.5 kV). The spectrometer was calibrated using an ESI-L low concentration tuning mix (Agilent) ranging from m/z of 112 to 2833 in a mixture of acetonitrile (HPLC grade) and DI (deionized, HPLC grade) H_2O (95:5 mixture). Samples were diluted to a concentration below ca. 1×10^{-6} M in an appropriate solvent using 1.5 mL glass vials capped with appropriate septum and then sampled by direct infusion using a 500 µL gas tight syringe. For **Me_6L** and **H_6L**, the samples were dissolved in

MeOH. For **IC1** and **IC2** the samples were dissolved in DI H_2O or a 1:1 DI H_2O:methanol solvent mixture, respectively.

CHN elemental microanalysis for **Me₆L** and **H₆L** was carried out by the elemental analysis services from the Metropolitan University of London using a ThermoFlash 2000 Elemental Analyzer. Several attempts to obtain meaningful elemental microanalysis data for **IC1** and **IC2** were attempted, however, due to the highly hygroscopic nature of these compounds the results systematically showed significant degrees of hydration $(H_2O)_n$ (n = 9–36).

S2 Ligand syntheses and characterization

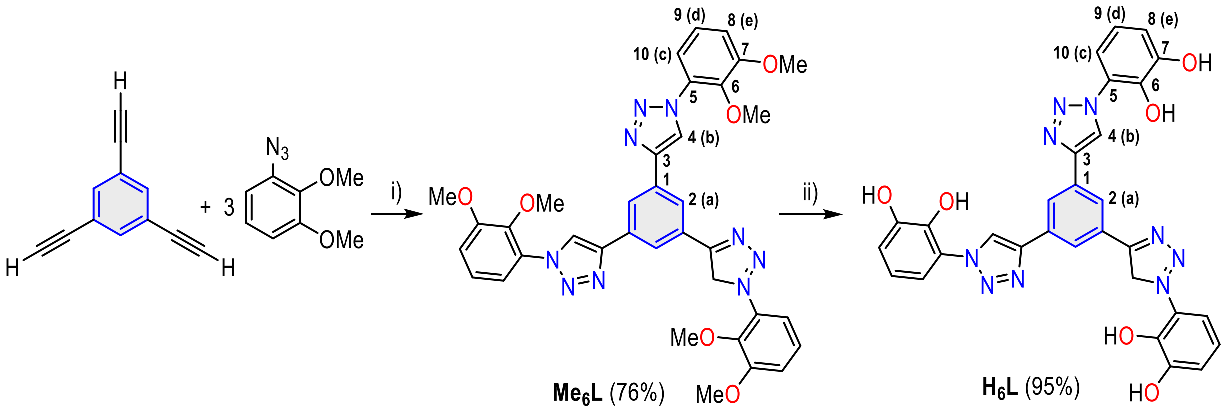

Scheme S1. Synthesis of ligands **Me$_6$L** and **H$_6$L**: i) $CuSO_4 \cdot 5H_2O$, L-ascorbic acid, *n*-BuOH/H_2O, 117ºC (72 h); ii) BBr_3 excess (16 equiv), CH_2Cl_2, –78ºC to RT (16 h).

S2.1 Synthesis of Me$_6$L

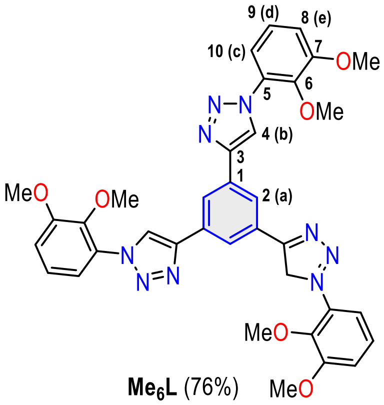

To a solution of 1,3,5-triethynylbenzene[1] (150 mg, 1 mmol) and 1-azido-2,3-dimethoxybenzene[2] (536 mg, 3 mmol) in a 1:1 mixture of *n*-butanol:water (15 mL) in a 100 mL round-bottom flask, L-ascorbic acid (40 mg, 0.2 mmol, 20 mol%) and $CuSO_4 \cdot 5H_2O$ (25 mg, 0.1 mmol, 10 mol%) were added. The reaction mixture was stirred at 117ºC until TLC showed the completion of the reaction (72 hours). Then, *n*-butanol was removed under reduced pressure, and the viscous residue was extracted with $CHCl_3$ (3 × 20 mL). The combined organic extractions were treated with saturated brine and anhydrous Na_2SO_4 and then organic extractions were filtered and $CHCl_3$ was removed under reduced pressure in a rotary evaporator to afford **Me$_6$L** as a light-brown solid (525 mg, 0.764 mmol, 76% yield). Crystals suitable for X-ray diffraction crystallographic study were obtained by slow evaporation of a concentrated solution of **Me$_6$L** (50 mM, 0.5 mL) in ethyl acetate after 24 hours.

^{1}H NMR (400 MHz, $CDCl_3$, 298 K): δ 8.57 (s, 3H, H4(b)), 8.51(s, 3H, H2(a)), 7.45 (d, 3H, H8(e), $^3J_{H8(e)-H9(d)}$ = 8.0 Hz), 7.22 (t, 3H, H9(d), $^3J_{H9(d)-H8(e)} = J_{H9(d)-H10(c)}$ = 8.0 Hz), 7.04 (d, 3H, H10(c), $^3J_{H10(c)-H9(d)}$ = 8.0 Hz), 3.96 (s, 9H, C7-OCH_3), 3.80 (s, 9H, C6-OCH_3).

^{13}C{^{1}H} NMR (100.6 MHz, $CDCl_3$, 298 K): δ 153.8 (C7), 147.1 (C1 or C3), 141.6 (C6), 132.0 (C1 or C3), 131.2 (C5), 124.5 (CH9(d)), 122.9 (CH2), 122.3 (CH4(b)), 117.1 (CH8), 113.1 (CH10(c)), 61.4 (C7-OCH_3), 56.3 (C6-OCH_3).

IR (KBr/Nujol, cm^{-1}): ν(C=N, C=C) 1612, 1595, 1488.

Elemental analysis found (calculated): $C_{36}H_{35}N_9O_7$ ($C_{36}H_{33}N_9O_6 \cdot H_2O$); C, 61.43 (61.27); H, 4.84 (5.00); N, 17.66 (17.86).

HRMS-QTOF-ESI (positive-ion detection) m/z found (calculated) for $C_{36}H_{33}N_9O_6$ [M]: $[M + Na]^+$, 710.2452 (710.2446); $[M + H]^+$, 688.2630 (688.2627).

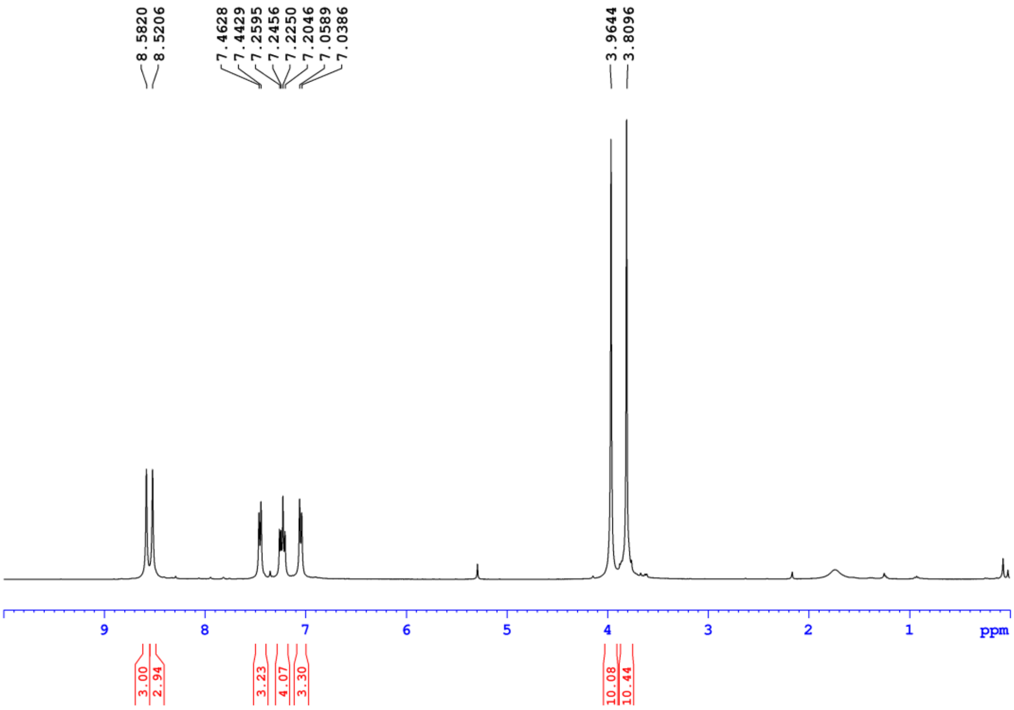

Figure S1. ^{1}H NMR (400 MHz, $CDCl_3$, 298 K) spectrum of **Me_6L**.

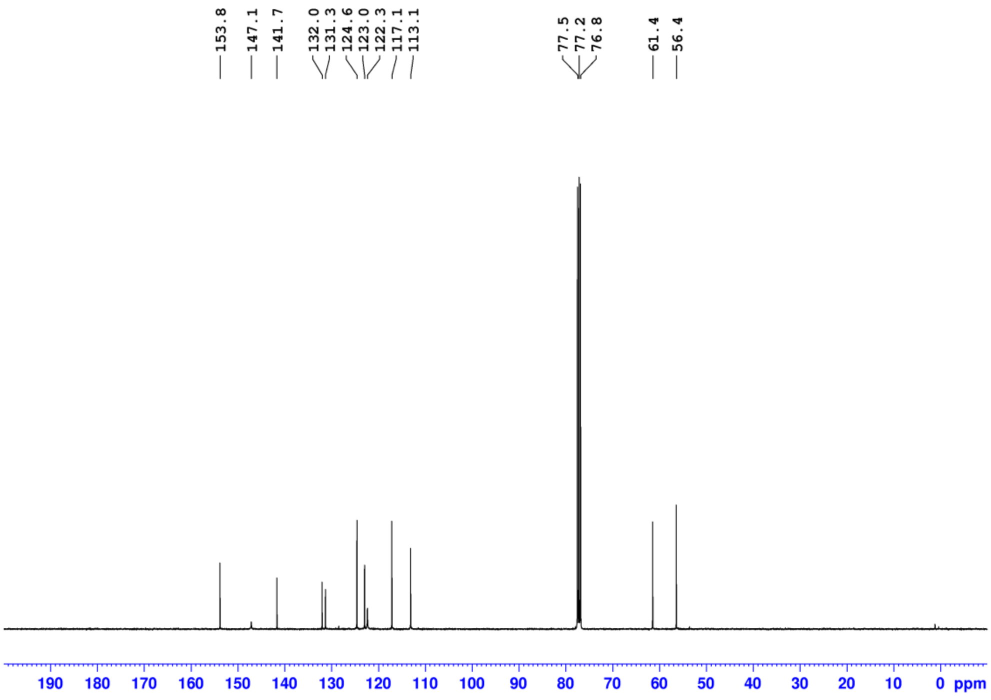

Figure S2. $^{13}C\{^{1}H\}$ NMR (100.6 MHz, $CDCl_3$, 298 K) spectrum of **Me_6L**.

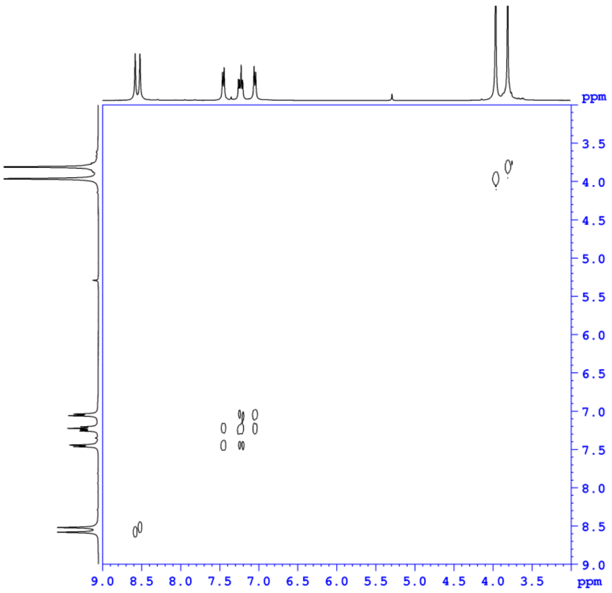

Figure S3. $^{1}H,^{1}H$-COSY NMR (400 MHz, $CDCl_3$, 298 K) spectrum of **Me_6L**.

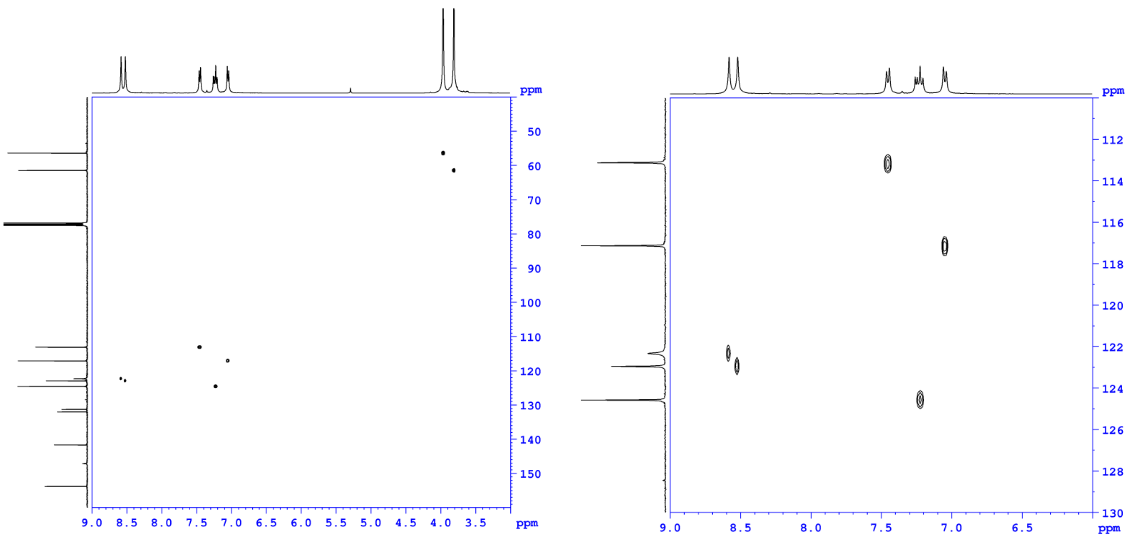

Figure S4. Selected sections of the $^{1}H,^{13}C$-HSQC (400 MHz, $CDCl_3$, 298 K) spectrum of **Me_6L**.

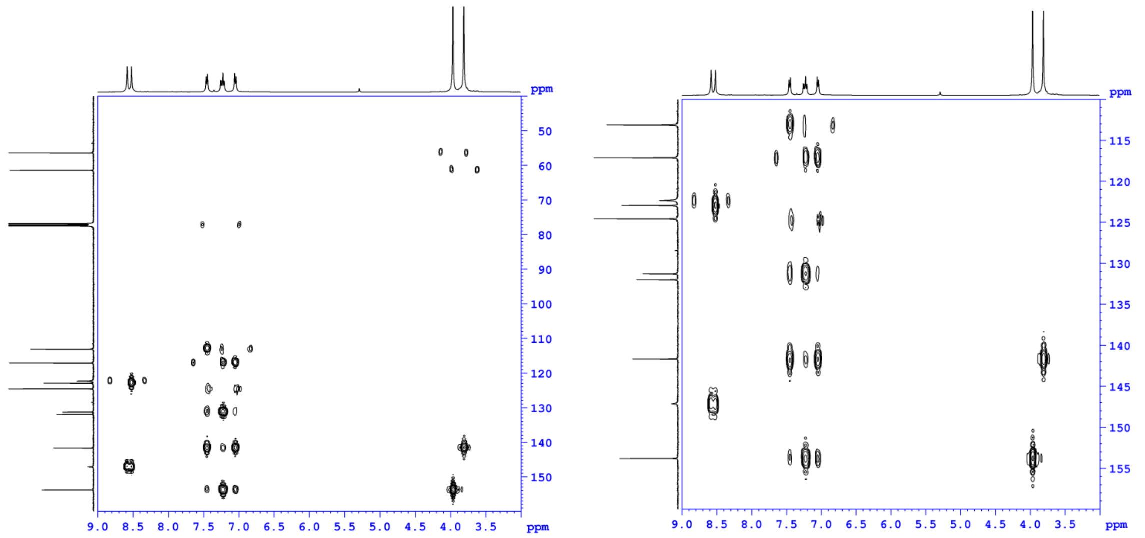

Figure S5. Selected sections of the $^{1}H,^{13}C$-HMBC (400 MHz, $CDCl_3$, 298 K) spectrum of **Me_6L**.

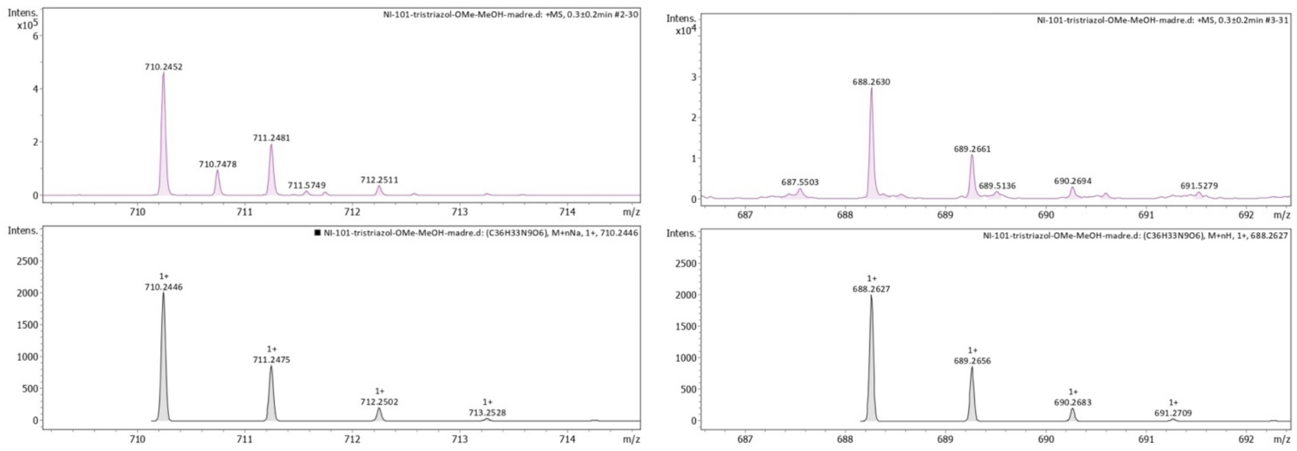

Figure S6. High-resolution mass spectra (ESI) data of **Me_6L** corresponding to the species [**Me_6L** + Na]$^+$ (left) and [**Me_6L** + H]$^+$ (right). Top: experimental; Bottom: calculated.

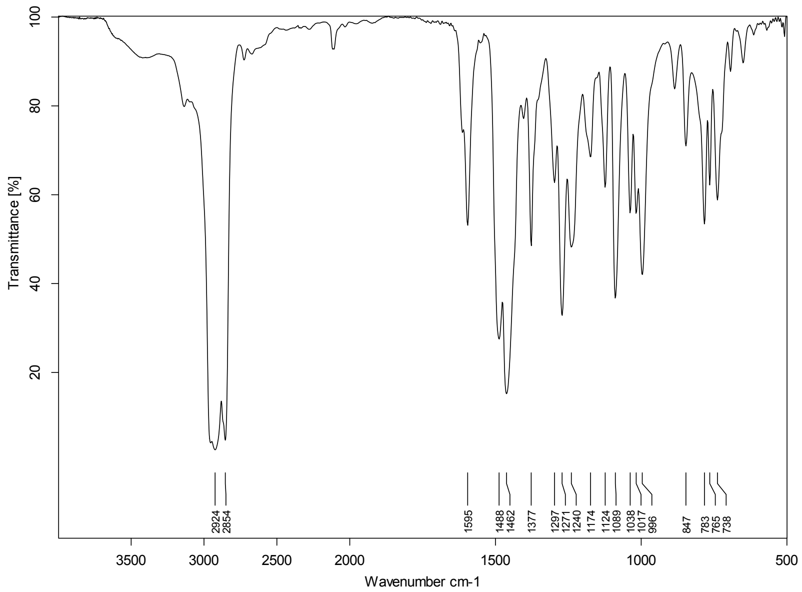

Figure S7. IR (KBr/Nujol, cm^{-1}) spectrum of **Me$_6$L**.

S2.2 Synthesis of H$_6$L

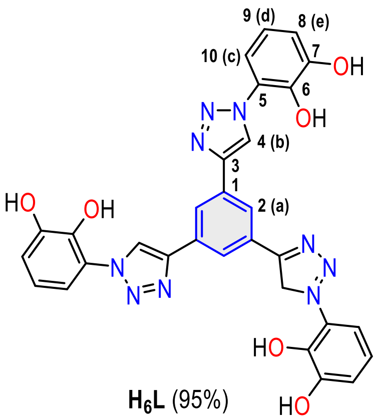

H$_6$L (95%)

To a solution of **Me$_6$L** (500 mg, 0.73 mmol) in CH_2Cl_2 (35 mL) under an atmosphere of nitrogen gas in a cold acetone bath at −78 ºC, BBr_3 (1.12 mL, 11.6 mmol) was added via syringe. The reaction mixture was allowed to warm to room temperature and stirred for 16 hours. All volatiles were removed under vacuum, and the resulting brown residue was stirred in water at 100ºC for 2 hours. The resulting light-brown precipitate was collected by filtration and dried under high vacuum (< 1 × 10^{-2} mbar) at 70ºC for 6 hours to give **H$_6$L** as a light brown solid (420 mg, 0.696 mmol, 95% yield). **H$_6$L** is a highly hygroscopic compound and was stored in a glove box under an atmosphere of dry nitrogen gas.

^{1}H NMR (400 MHz, DMSO-*d*$_6$, 298 K; high concentration: 70 mM): δ 11.00-8.7 (v br s, 6H, OH), 9.08 (s, 3H, H4(b)), 8.52 (s, 3H, H2(a)), 7.12 (d, 3H, H8(e), $^3J_{H8(e)\text{-}H9(d)}$ = 7.9 Hz), 6.99 (d, 3H, H10(c), $^3J_{H10(c)\text{-}H9(d)}$ = 7.9 Hz), 6.86 (t, 3H, H9(d), $^3J_{H9(d)\text{-}H8(d)}$ = $J_{H9(d)\text{-}H10(c)}$ = 7.9 Hz).

^{1}H NMR (400 MHz, DMSO-*d*$_6$, 298 K; low concentration: 15 mM): δ 10.05 (s, 3H, OH), 9.52 (s, 3H, OH), 9.08 (s, 3H, H4(b)), 8.52 (s, 3H, H2(a)), 7.12 (d, 3H, H8(e), $^3J_{H8(e)\text{-}H9(d)}$ = 7.9 Hz), 6.99 (d, 3H, H10(c), $^3J_{H10(c)\text{-}H9(d)}$ = 7.9 Hz), 6.86 (t, 3H, H9(d), $^3J_{H9(d)\text{-}H8(e)}$ = $J_{H9(d)\text{-}H10(c)}$ = 7.9 Hz).

^{13}C{^{1}H} NMR (100.6 MHz, DMSO-*d*$_6$, 298 K): δ 146.7 (C7), 145.6 (C3), 138.8 (C6), 132.0 (C1), 125.4 (C5), 123.6 (CH4(b)), 121.5 (CH2), 119.1 (CH9(d)), 115.8 (CH10(c)), 115.6 (CH8(e)).

IR (KBr/Nujol, cm^{-1}): ν(O-H) 3498 (br); ν(C=N, C=C) 1620, 1520, 1491.

Elemental analysis found (calculated): $C_{30}H_{28}N_9O_{9.5}$ ($C_{30}H_{21}N_9O_6 + (H_2O)_{3.5}$); C, 54.25 (54.05); H, 4.00 (4.23); N, 18.68 (18.91).

HRMS-QTOF-ESI (positive-ion detection) m/z found (calculated) for $C_{30}H_{21}N_9O_6$ [M]: $[M + H]^+$, 604.1692 (604.1688).

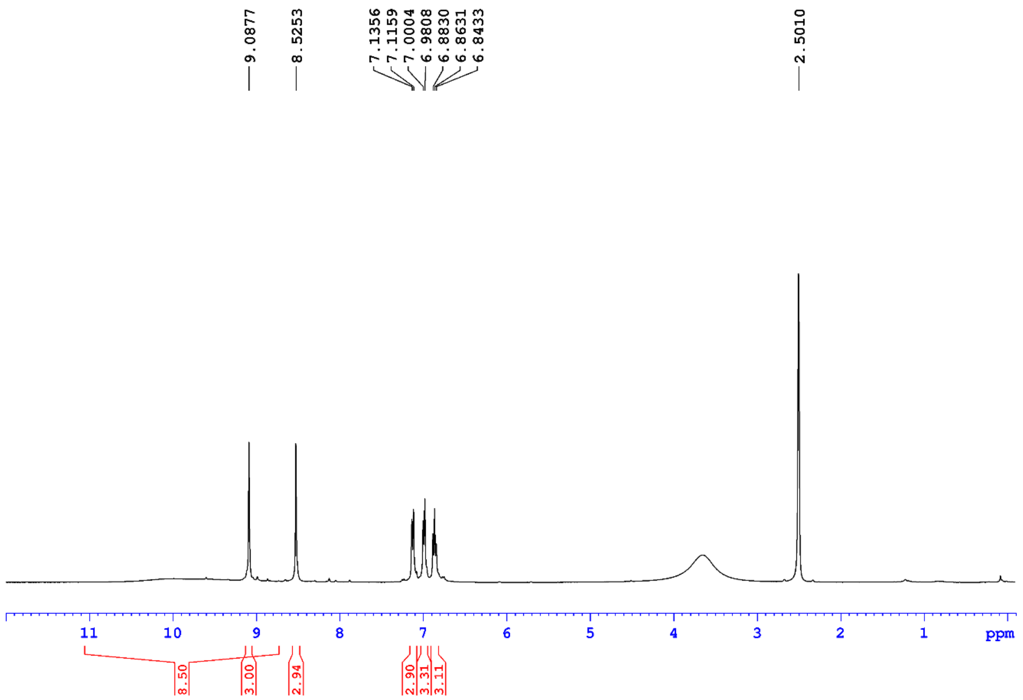

Figure S8. ^{1}H NMR (400 MHz, DMSO-d_6, 298 K; 70 mM) spectrum of **H_6L**.

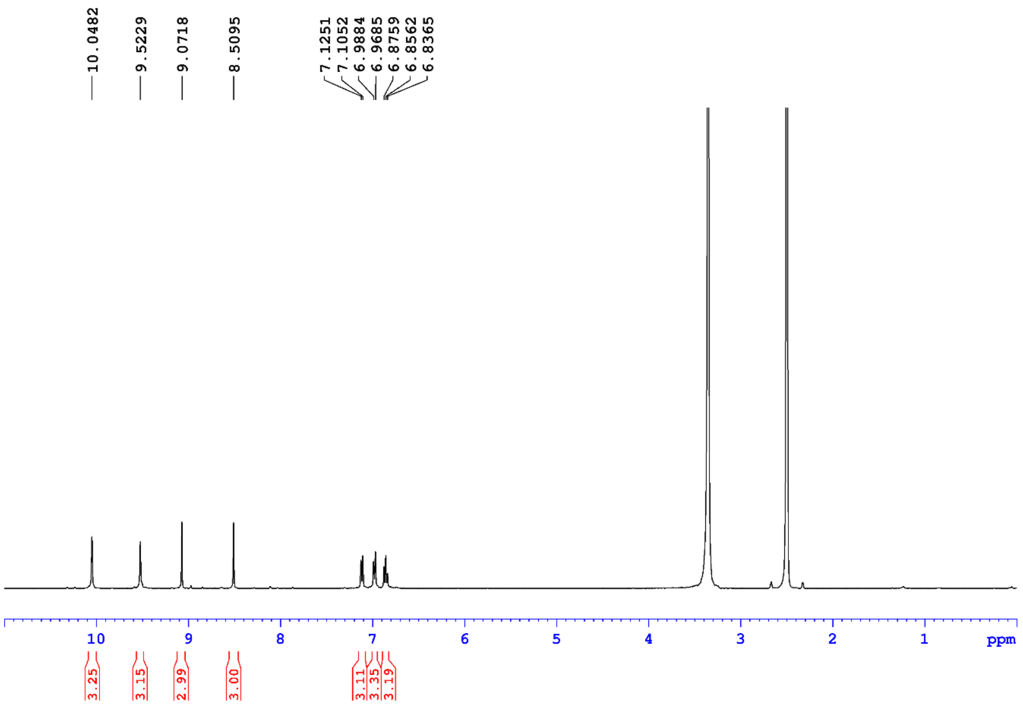

Figure S9. ^{1}H NMR (400 MHz, DMSO-d_6, 298 K; 15 mM) spectrum of **H_6L**.

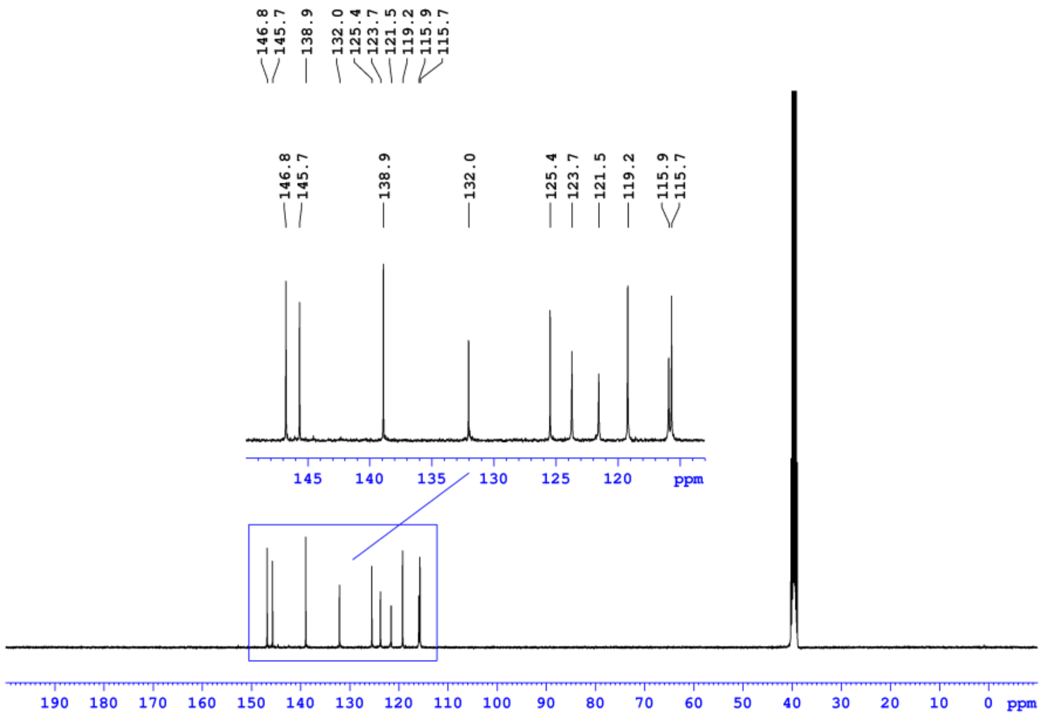

Figure S10. $^{13}C\{^1H\}$ NMR (100.6 MHz, DMSO-d_6, 298 K) spectrum of $\mathbf{H_6L}$.

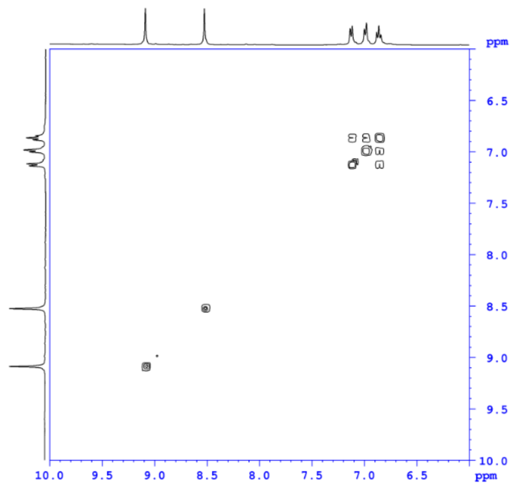

Figure S11. $^1H,^1H$-COSY NMR (400 MHz, DMSO-d_6, 298 K) spectrum of $\mathbf{H_6L}$.

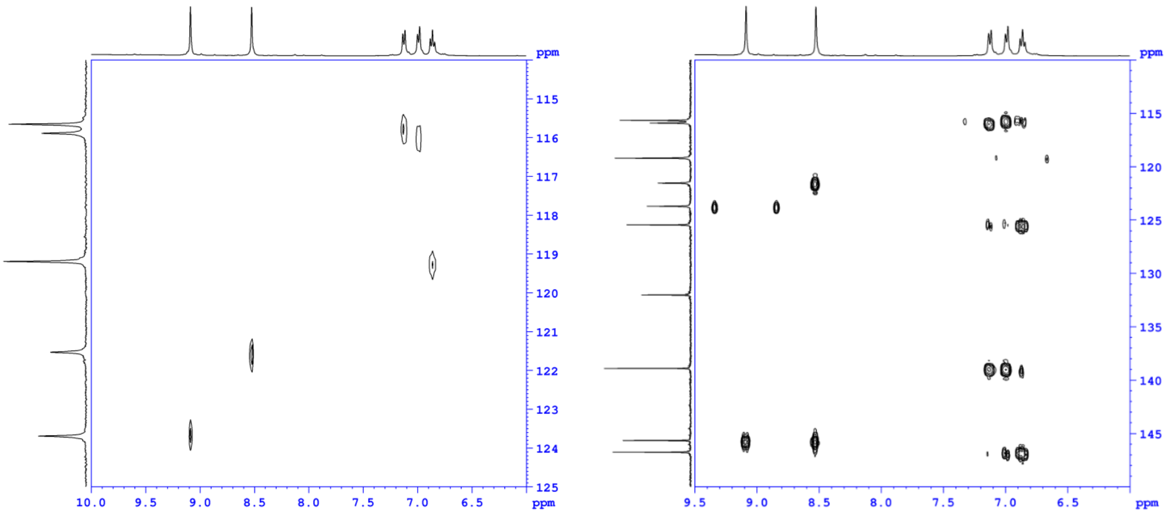

Figure S12. $^{1}H,^{13}C$-HSQC (left) and HMBC (right) spectra (400 MHz, DMSO-d_6, 298 K) of $\mathbf{H_6L}$.

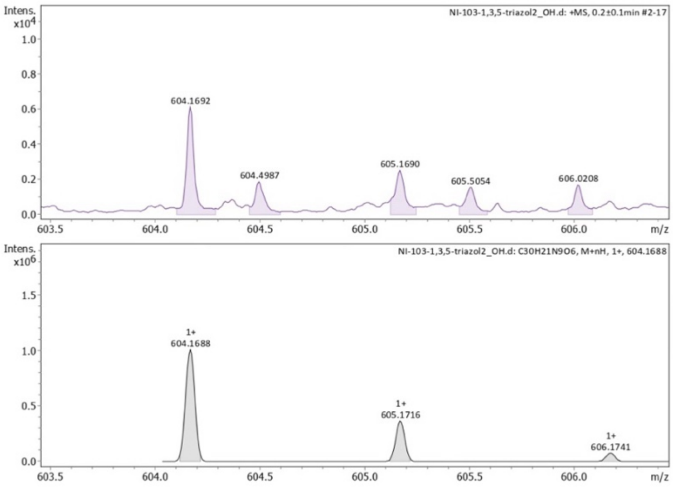

Figure S13. High-resolution mass spectra (ESI) of $\mathbf{H_6L}$ corresponding to the species [$\mathbf{H_6L}$ + H]$^+$. Top: experimental; Bottom: calculated.

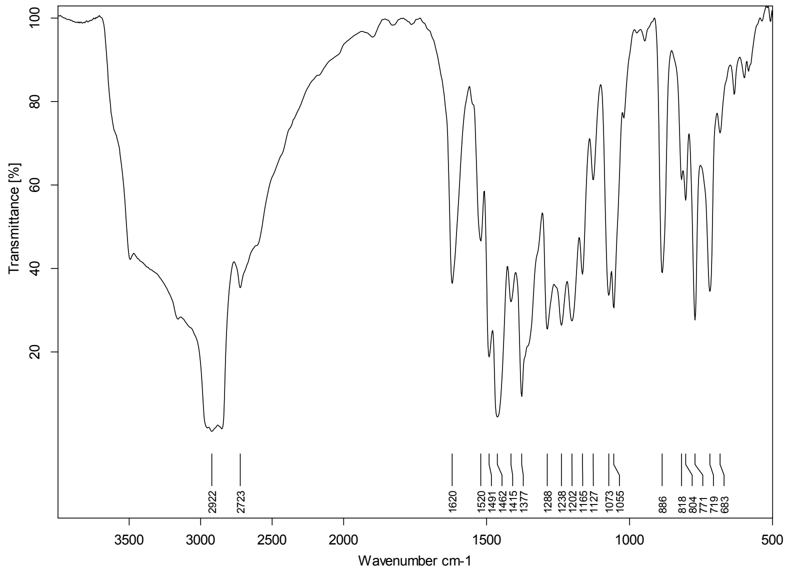

Figure S14. IR (KBr/Nujol, cm^{-1}) spectrum of **H_6L**.

S3 Self-assembly and characterization of coordination cages

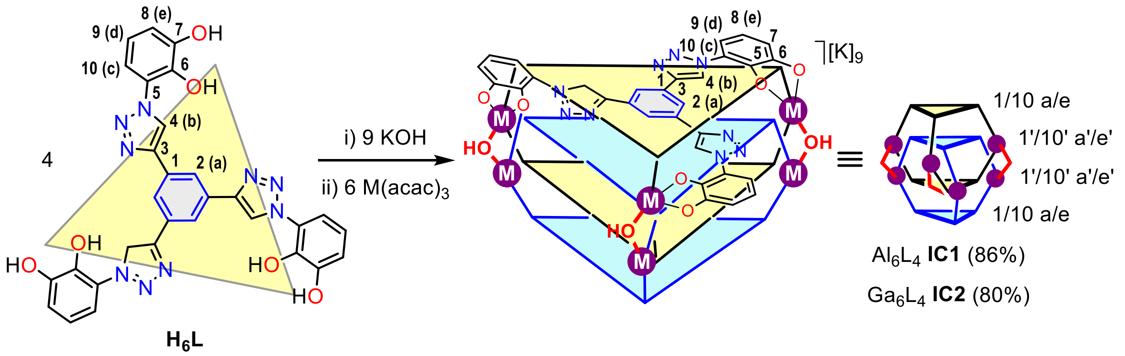

Scheme S2. Self-assembly of cages **IC1** and **IC2**.

S3.1 Self-assembly of IC1

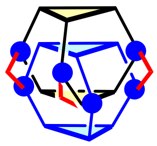

Al_6L_4 **IC1** (86%)

To a suspension of **H_6L** (100 mg, 0.166 mmol) in deoxygenated MeOH (25 mL) under an atmosphere of nitrogen gas, KOH was added (0.75 mL from a 0.5 M solution in MeOH, 0.373 mmol) was added via syringe. After 5 min of stirring a solution was obtained to which $Al(acac)_3$ (81 mg, 0.249 mmol) was then added under nitrogen gas. The reaction mixture was then stirred at room temperature for 16 hours. Then, the reaction mixture was concentrated under reduced pressure (5 mL) and then Et_2O (10 mL) was added to give a brown silky suspension. The solid was filtered and dried under high vacuum (< 1 × 10^{-2} mbar) for 6 hours to give $[Al_6L_4(OH)_3]K_9$ **IC1** as a light brown solid (106 mg, 0.0358 mmol, 86% yield). Due to its highly hygroscopic nature, **IC1** was stored in a glove box under dry nitrogen gas to prevent hydration of the

solid. *Note*: repeated the preparative self-assembly in methanol over 1-15 mM consistently remained high isolated yields and showed no systematic dependence on concentration.

^{1}H NMR (500 MHz, D_2O, 298 K): δ 9.42 (s, 6H, H4'(b')), 9.28 (s, 6H, H4(b)), 7.70 (s, 6H, H2(a)), 7.35 (s, 6H, H2'(a')), 6.91 (dd, 6H, H8(e) or H10(c), H8'(e') or H10', $^{3}J_{H\text{-}H}$ = 8.0 Hz, $^{4}J_{H\text{-}H}$ = 1.2 Hz), 6.67 (dd, 6H, H8(e) or H10(c), H8'(e') or H10'(c'), $^{3}J_{H\text{-}H}$ = 8.0 Hz, $^{4}J_{H\text{-}H}$ = 1.2 Hz), 6.62 ("t", 12H, H8(e) or H10(c), H8'(e') or H10'(c'), $^{3}J_{H\text{-}H}$ = 8.0 Hz), 6.50 (t, 6H, H9(d) or H9'(d'), $^{3}J_{H\text{-}H}$= 8.0 Hz), 6.46 (t, 6H, H9(d) or H9'(d'), $^{3}J_{H\text{-}H}$= 8.0 Hz).

^{1}H NMR (400 MHz, CD_3OD, 298 K): δ 9.12 (br s, 12H, H4(b+b')), 8.43 (br s, 12H, H2(a+a')), 6.85 (v br d, 12H, H8(e+e') or H10(c+c'), $^{3}J_{H\text{-}H}$ = 7.4 Hz), 6.59 (v br d, 12H, H8(e+e') or H10(c+c'), $^{3}J_{H\text{-}H}$ = 7.5 Hz), 6.47 (v br t, 12H, H9(d+d'), $^{3}J_{H\text{-}H}$= 7.5 Hz).

^{1}H NMR (400 MHz, CD_3OD, 238 K): δ 9.04–9.31 (several br s, 12H, H4(b+b')), 8.38–8.80 (several br s, 12H, H2(a+a')), 6.39–6.92 (several br m, 12H, H8(e+e') + H9(d+d') + H10(c+c')).

^{13}C{^{1}H} NMR (125.6 MHz, D_2O, 298 K): δ 155.0 (C7 or C7'), 153.9 (C7 or C7'), 146.6 (C6 or C6'), 146.2 (C6 or C6'), 145.9 (C1), 144.3 (C1'), 131.1 (C3 or C3'), 130.7 (C3 or C3'), 123.6 (CH4(b)), 122.5 (CH2), 122.0 (C5 or C5'), 121.6 (CH4'(b')), 121.4 (C5 or C5'),121.2 (CH2'), 115.5 (CH9(d) or CH9'(d')), 115.2 (CH9(d) or CH9'(d')), 111.7 (CH8(e) or CH8'(e'), CH10(c) or CH10'(c')), 111.5 (CH8(e) or CH8'(e'), CH10(c) or CH10'(c')), 110.9 (CH8(e) or CH8'(e'), CH10(c) or CH10'(c')), 110.3 (CH8(e) or CH8'(e'), CH10(c) or CH10'(c')).

IR (KBr/Nujol, cm^{-1}): ν(O-H) 3386 (v br); ν(C=N, C=C) 1585 (br), 1532.

HRMS-QTOF-ESI (negative-ion detection, H_2O, ⧉$^{6-}$ = $[L_4Al_6]^{6-}$, □$^{3-}$ = $[L_2Al_3]^{3-}$): m/z found (calc.), [□$^{3-}$] = 425.0564 (425.0584); [□$^{3-}$ + $K^+]^{2-}$ = 657.0654 (657.0692); [□$^{3-}$ + OH^- + $K^+]^{2-}$ = 685.0490 (685.0525); [□$^{3-}$ + 4 OH^- + 1 K^+ + 3 Na^+ + $H^+]^{2-}$ = 726.0689 (726.0633); [⧉$^{6-}$ + 3 OH^- + 5 K^+ + 1 $Na^+]^{3-}$ = 940.0548 (940.0561); [⧉$^{6-}$ + 3 OH^- + 6 $K^+]^{3-}$ = 945.7085 (945.7143); [⧉$^{6-}$ + 3 OH^- + 6 K^+ + 1 $Na^+]^{2-}$ = 1430.0573 (1430.0661); [⧉$^{6-}$ + 3 OH^- + 7 $K^+]^{2-}$ = 1438.0436 (1438.0529).

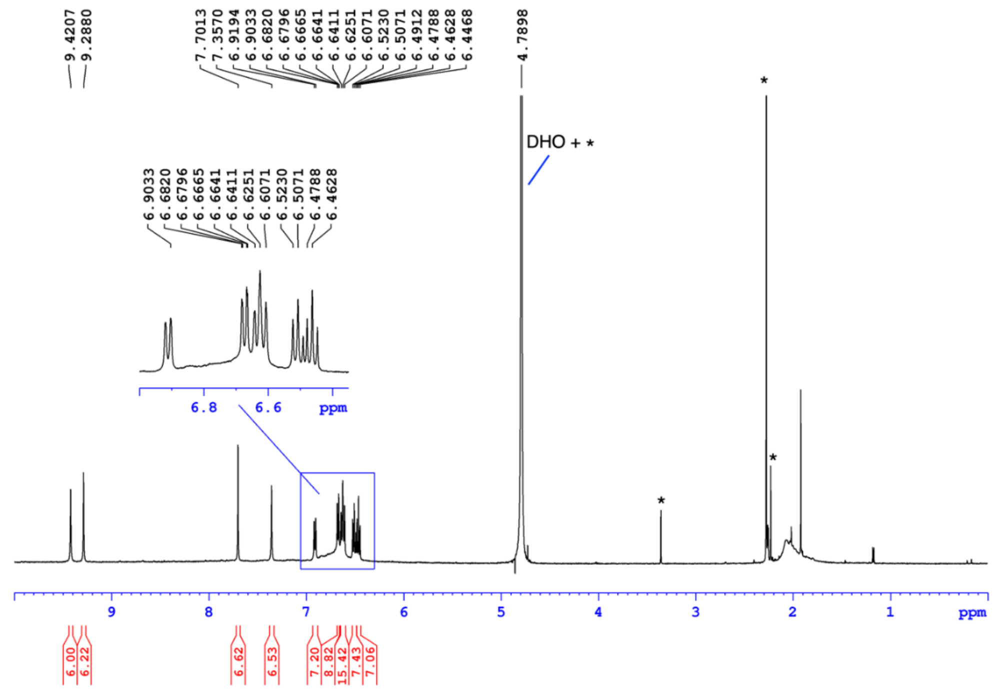

Figure S15. ^{1}H NMR (500 MHz, D_2O, 298 K) spectrum of **IC1**. The symbol * denotes small residual signal from acetylacetone from isolation.

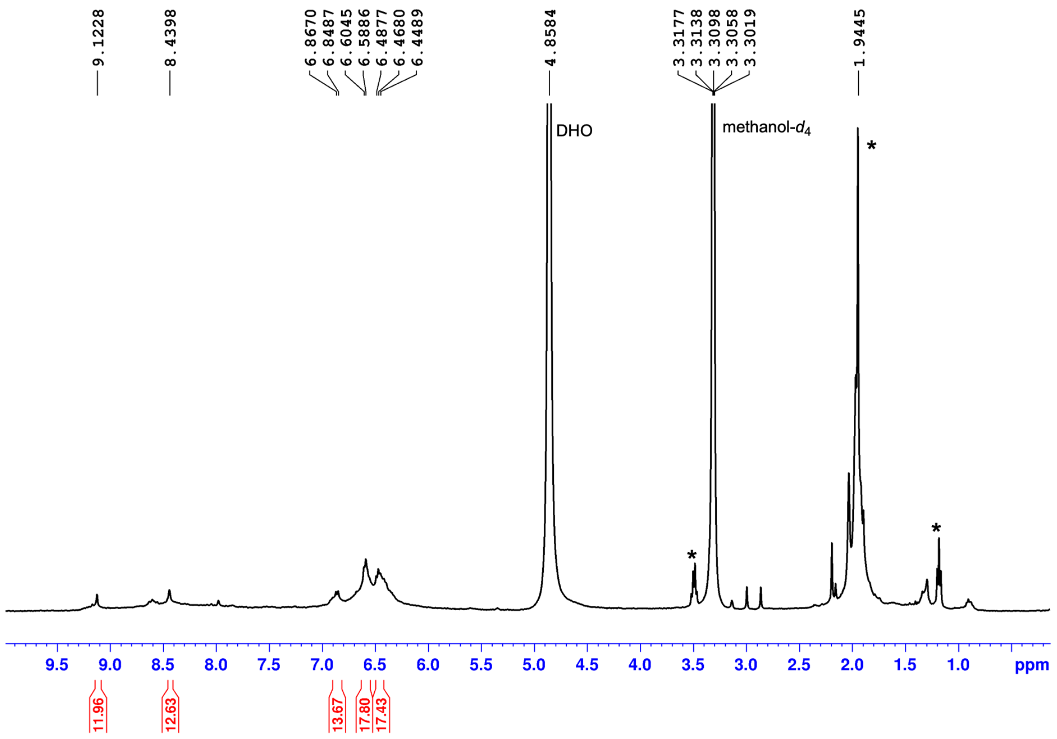

Figure S16. ^{1}H NMR (400 MHz, CD_3OD, 298 K) spectrum of **IC1**. The symbol * denotes small residual signal from acetylacetone and diethyl ether from isolation.

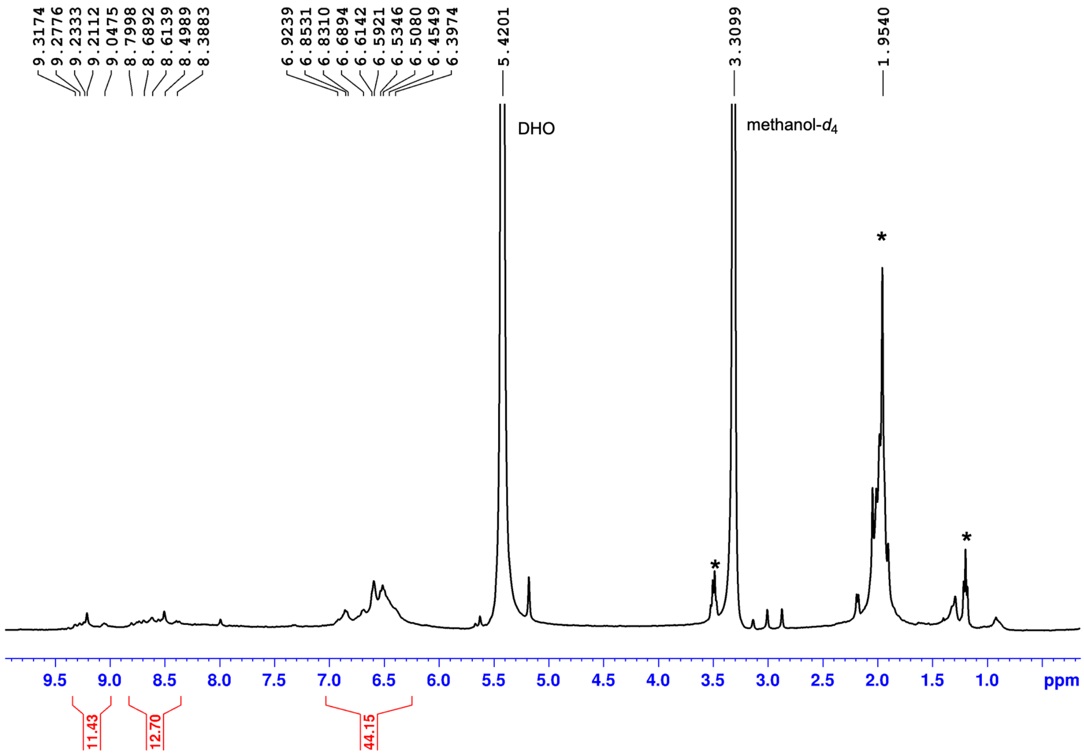

Figure S17. ^{1}H NMR (400 MHz, CD_3OD, 238 K) spectrum of **IC1**. The symbol * denotates small residual signal from acetylacetone and diethyl ether from isolation.

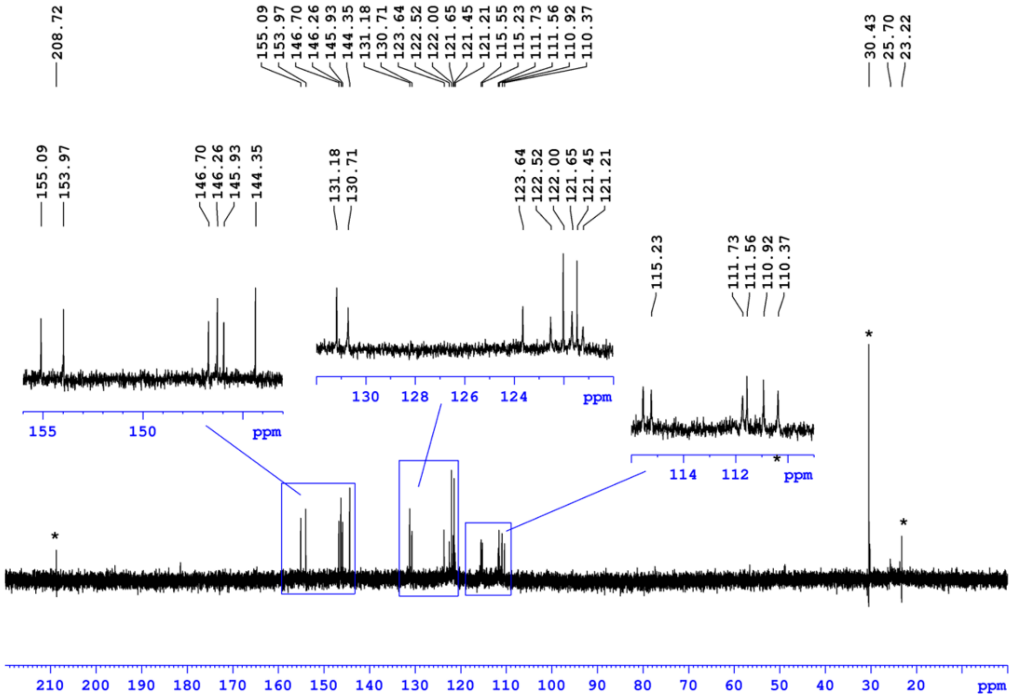

Figure S18. $^{13}C\{^1H\}$ NMR (125.6 MHz, D_2O, 298 K) spectrum of **IC1**. The symbol * denotates small residual signal from acetylacetone from isolation.

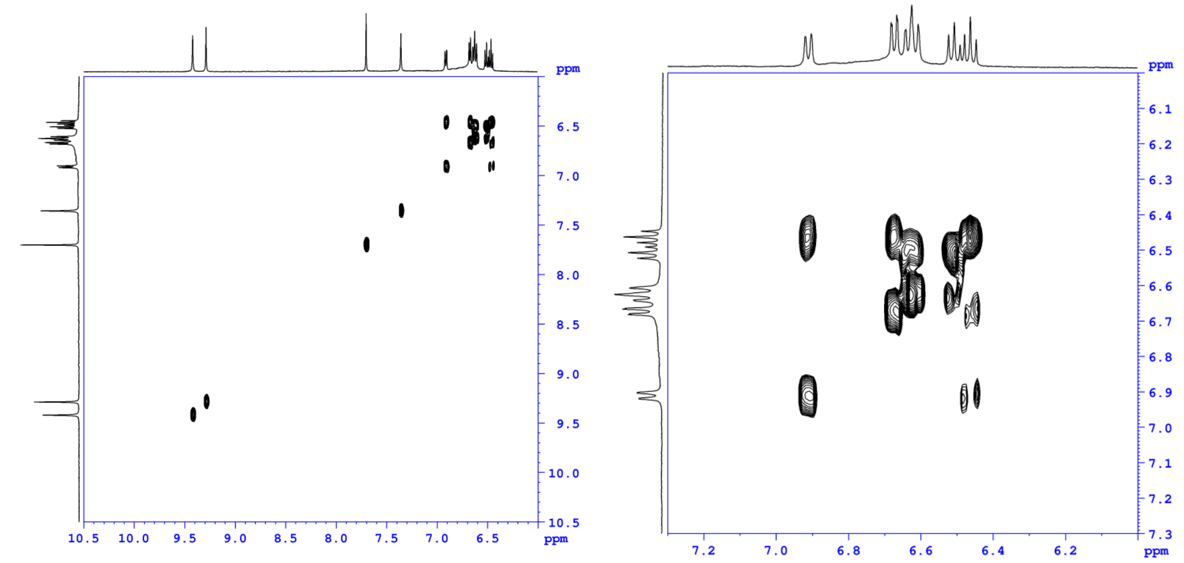

Figure S19. Selected sections for the $^{1}H,^{1}H$-COSY NMR (500 MHz, D_2O, 298 K) spectrum of **IC1**.

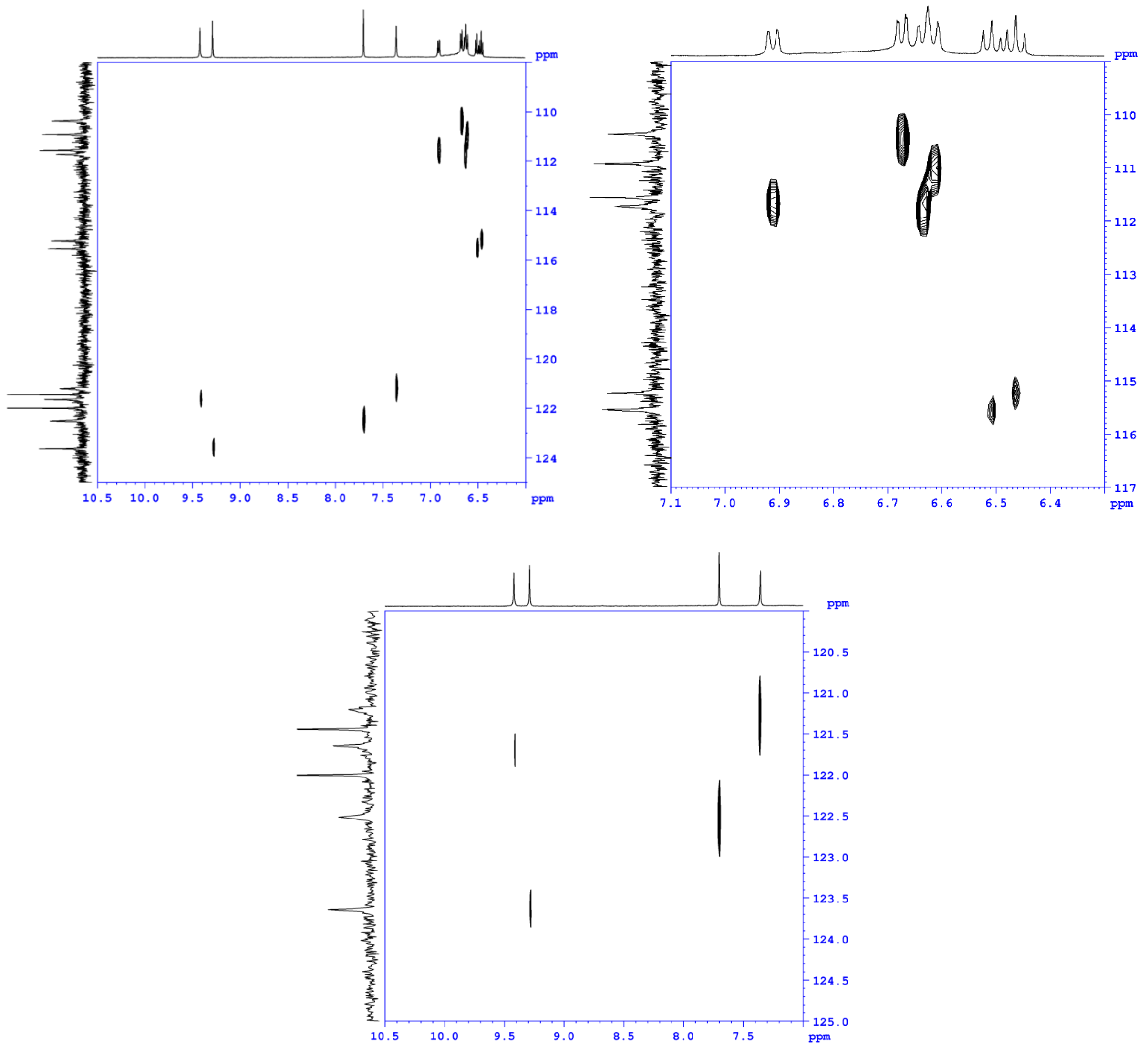

Figure S20. Selected sections of the $^{1}H,^{13}C$-HSQC (500 MHz, D_2O, 298 K) spectrum of **IC1**.

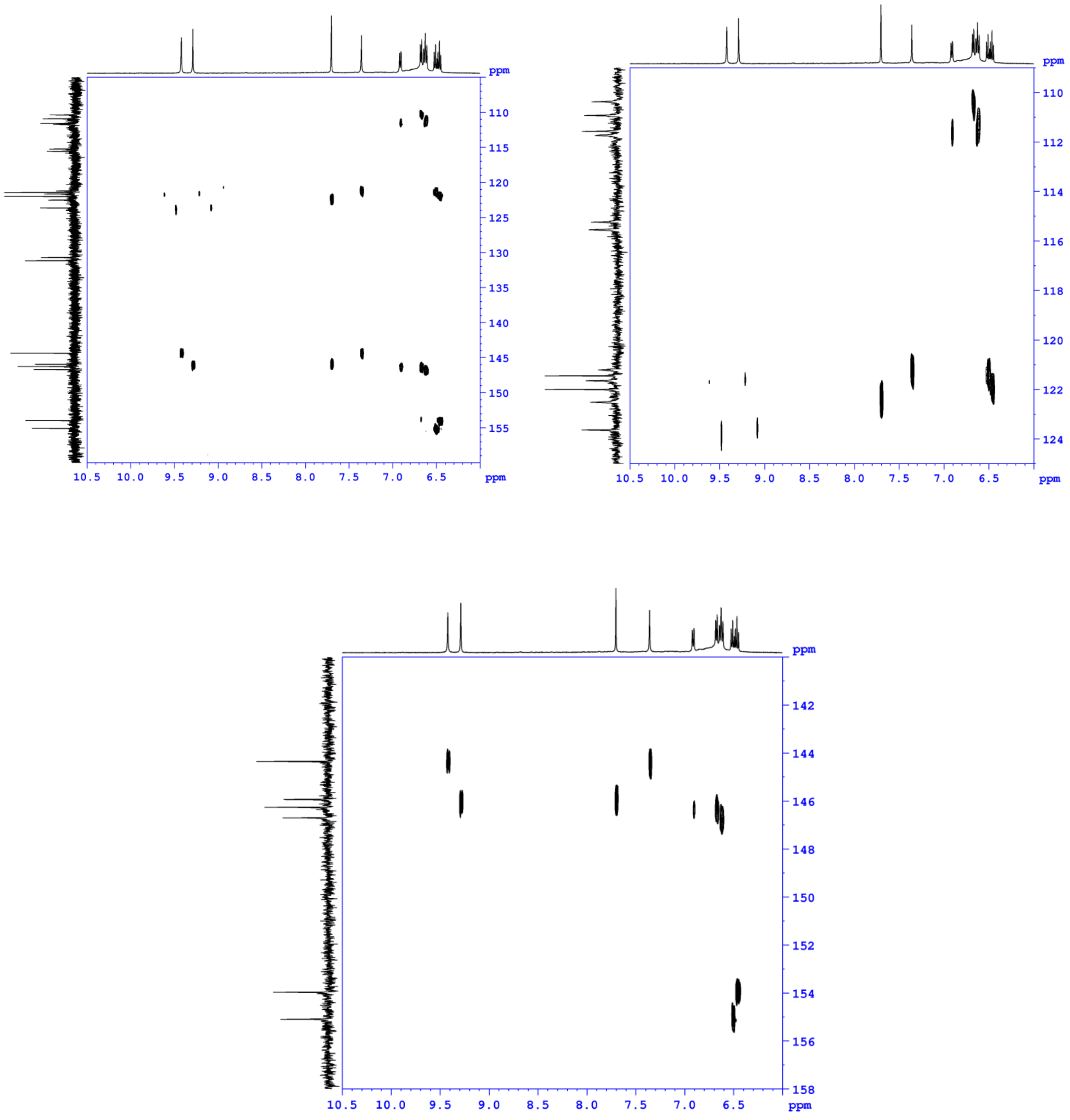

Figure S21. Selected sections for the ^{1}H,^{13}C-HMBC (500 MHz, D_2O, 298 K) spectrum of **IC1**.

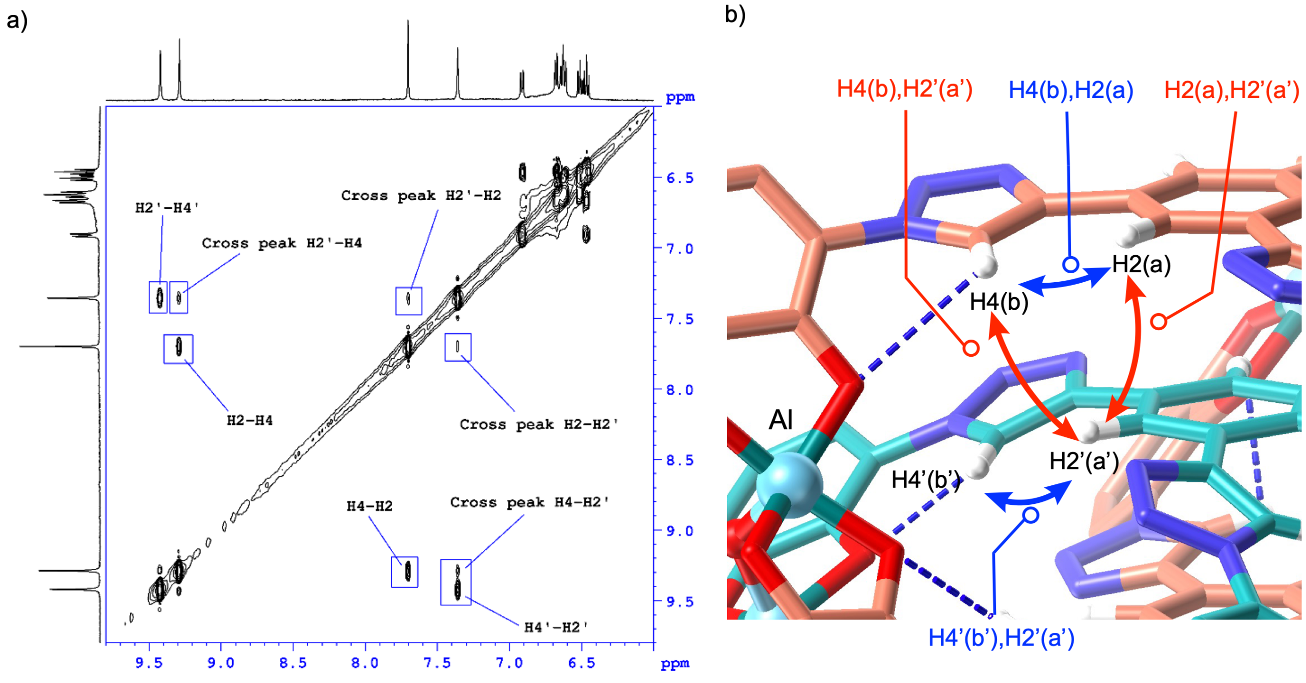

Figure S22. a) ^{1}H,^{1}H-NOESY spectrum (500 MHz, D_2O, 298 K) of **IC1**. NOE couplings are highlighted with a blue box. The highlighted cross peaks H4(b)-H2'(a') and H2(a)-H2'(a')/H2'(a')-H2(a) indicate ^{1}H atoms that are close between the inner and outer ligand decks (ie. they do not belong to the same ligand system) within the supramolecular molecular geometry of **IC1**. b) Illustration of NOE interactions in the computed structure of **IC1.**

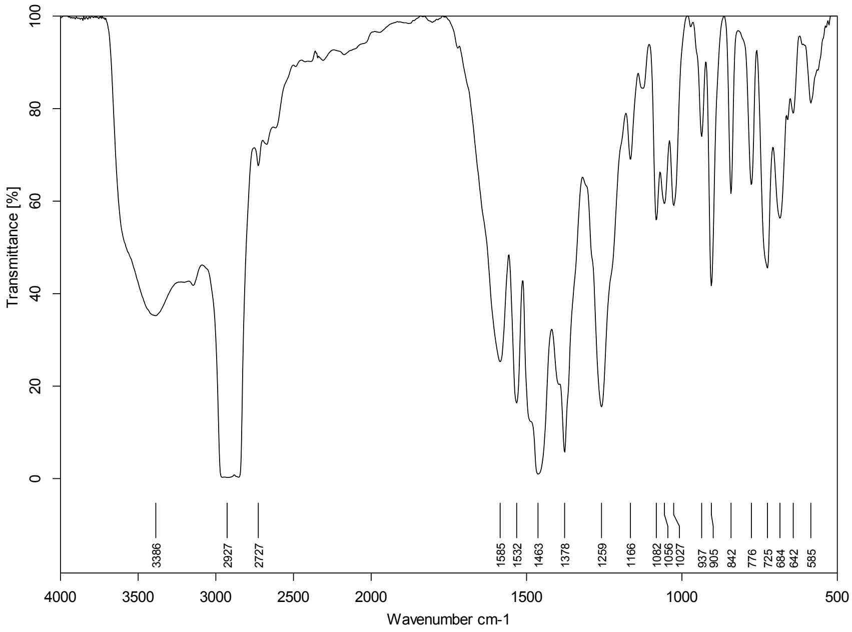

Figure S23. IR (KBr/Nujol, cm^{-1}) spectrum of **IC1**.

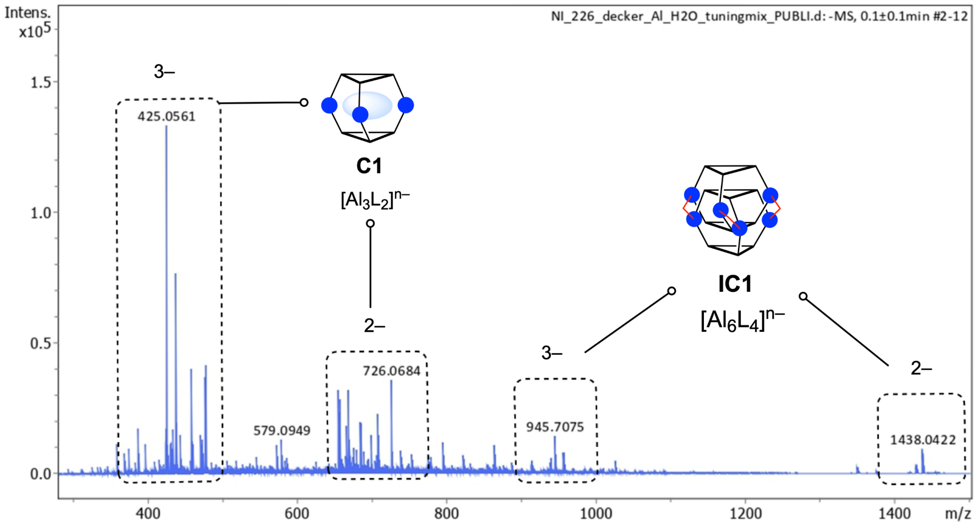

Figure S24. Full view for HRMS-ESI (negative-ion detection mode) of **IC1**. Selected sections highlighted with a dashed box are those areas of interest that were fully studied in detail.

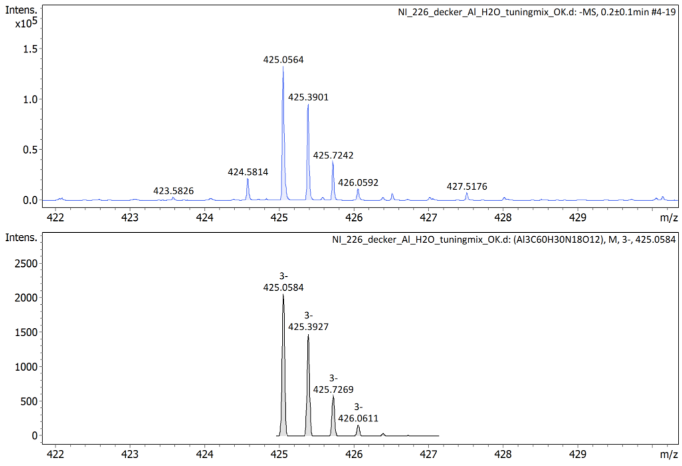

Figure S25. Section of the HRMS-ESI (negative-ion detection mode) corresponding to the species $\mathbf{[Al_3L_2]^{3-}}$. Top: experimental; bottom: calculated.

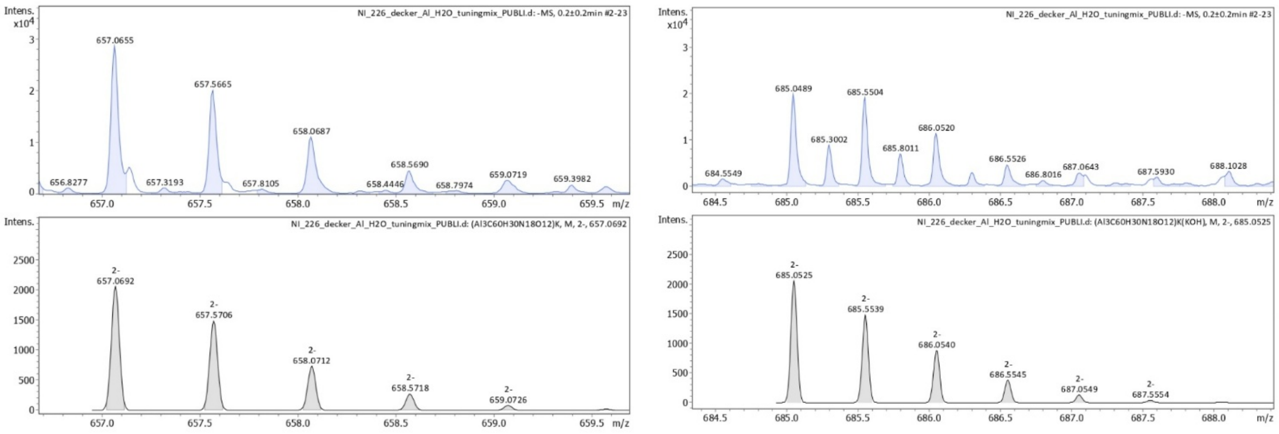

Figure S26. Sections of the HRMS-ESI (negative-ion detection mode) corresponding to the species $\mathbf{[Al_3L_2]^{3-} + K^+}$ (left) and $\mathbf{[Al_3L_2(OH)]^{4-} + 2\ K^+}$ (right). Top: experimental; bottom: calculated.

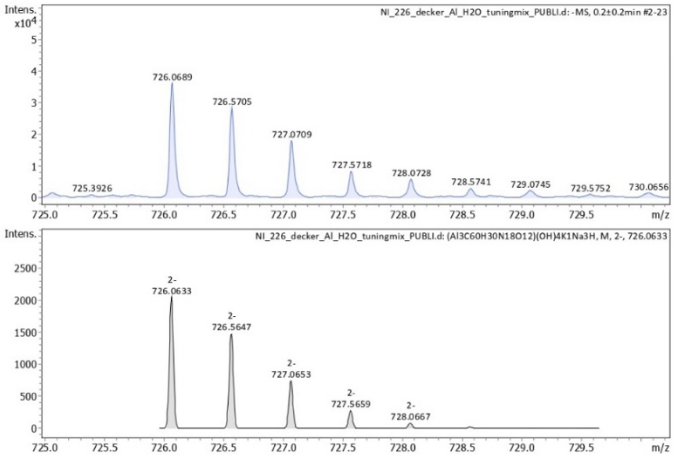

Figure S27. Sections of the HRMS-ESI (negative-ion detection mode) corresponding to the species **$[Al_3L_2(OH)_3]^{6-}$ + OH^- + K^+ + 3 Na^+ + H^+**. Top: experimental; bottom: calculated.

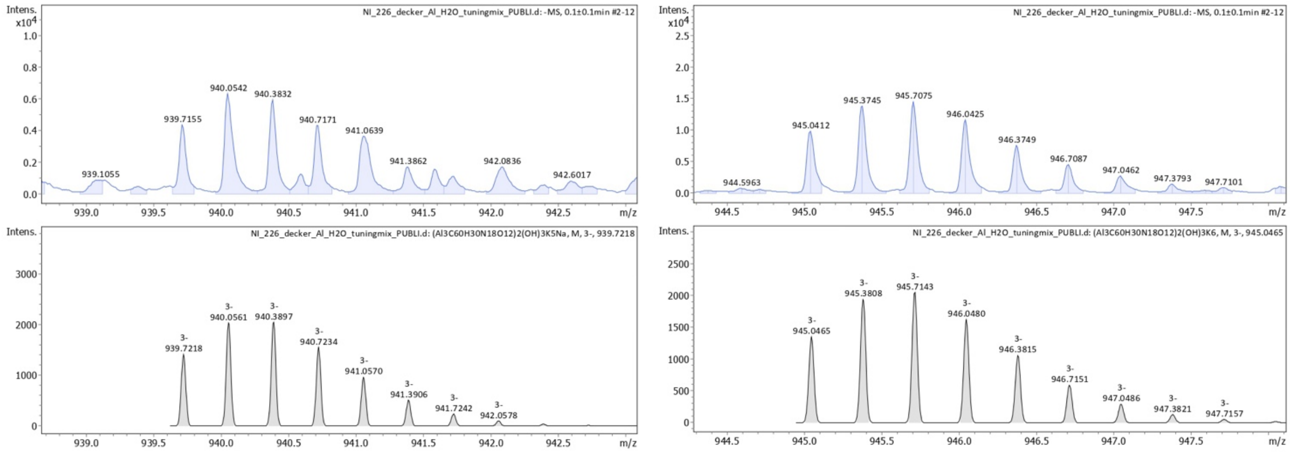

Figure S28. Sections of the HRMS-ESI (negative-ion mode) corresponding to **$[Al_6L_4(OH)_3]^{9-}$ + 5 K^+ + Na^+** (left) and **$[Al_6L_4(OH)_3]^{9-}$ + 6 K^+** (right). Top: experimental; bottom: calculated.

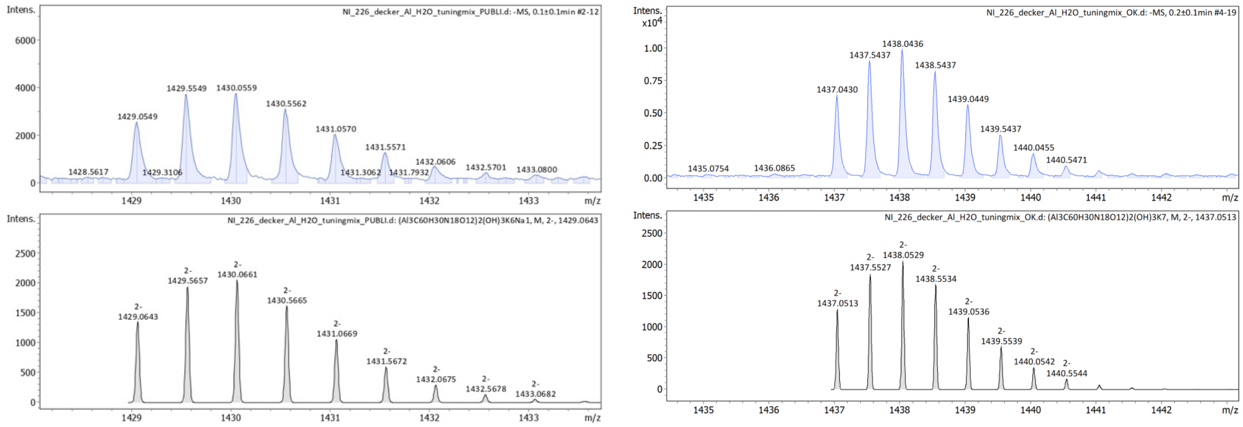

Figure S29. Sections of the HRMS-ESI (negative-ion mode) corresponding to **$[Al_6L_4(OH)_3]^{9-}$ + 6 K^++ Na^+** (left) and **$[Al_6L_4(OH)_3]^{2-}$ + 7K^+** (right). Top: experimental; bottom: calculated.

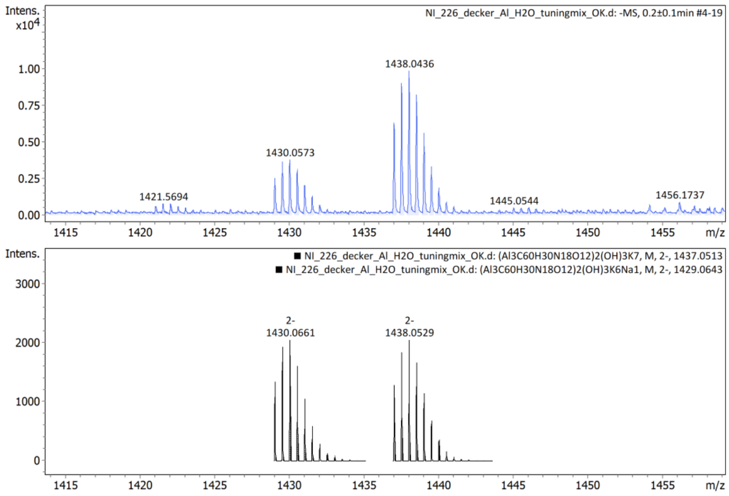

Figure S30. Section of the HRMS-ESI (negative-ion detection mode) corresponding to the species **$[Al_6L_4(OH)_3]^{9-}$ + 7 K^+** and **$[Al_6L_4(OH)_3]^{9-}$ + 6 K^++ Na^+**. Top: experimental; bottom: calculated.

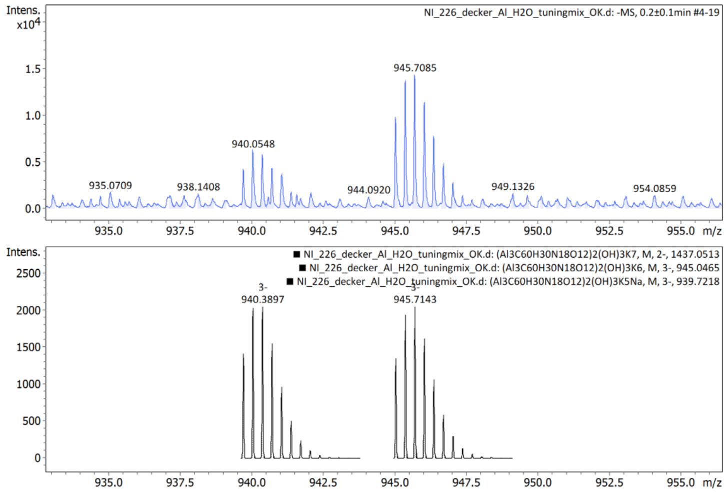

Figure S31. Section of the HRMS-ESI (negative-ion detection mode) corresponding to the species **$[Al_6L_4(OH)_3]^{9-}$ + 6 K^+** and **$[Al_6L_4(OH)_3]^{9-}$ + 5 K^++ Na^+**. Top: experimental; bottom: calculated.

S3.2 Self-assembly of IC2

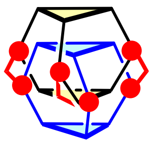

Ga_6L_4 **IC2** (80%)

Following a similar procedure than that of **IC1**, to a solution of **H_6L** (100 mg, 0.166 mmol) in deoxygenated MeOH (25 mL) under an atmosphere of nitrogen gas, KOH was added (0.75 mL from a 0.5 M solution in MeOH, 0.373 mmol) was added via syringe. After 5 min of stirring a solution was obtained to which $Ga(acac)_3$ (91 mg, 0.249 mmol) was then added under nitrogen gas. The reaction mixture was then stirred at room temperature for 16 hours. Then, the reaction mixture was concentrated under reduced pressure (5 mL) and then Et_2O (10 mL) was added to give a brown silky suspension. The solid was filtered and dried under high vacuum (< 1 × 10^{-2} mbar) for 6 hours to give $[Ga_6L_4(OH)_3]K_9$ **IC2** as a light brown solid (107 mg, 0.0332 mmol, 80% yield). Due to its highly hygroscopic nature, **IC2** was stored in a glove box

under dry nitrogen gas to prevent hydration of the solid. *Note*: repeated the preparative self-assembly in methanol over 1-15 mM consistently remained high isolated yields and showed no systematic dependence on concentration. Crystals suitable for an X-ray diffraction crystallographic study were obtained by vapor diffusion of acetone (2 mL) into a solution of **IC2**) in a 1:1 mixture of water and methanol (10 mM, 0.5 mL) after 7 days.

^{1}H NMR (500 MHz, D_2O, 298 K): δ 9.36 (s, 6H, H4’(b’)), 9.26 (s, 6H, H4(b)), 7.71 (s, 6H, H2(a)), 7.38 (s, 6H, H2’(a’)), 6.89 (dd, 6H, H8(e) or H10(c), H8’(e’) or H10’(c’), $^3J_{H\text{-}H}$ = 8.0 Hz, $^4J_{H\text{-}H}$ = 1.2 Hz), 6.73 (dd, 6H, H8(e) or H10(c), H8’(e’) or H10’(c’), $^3J_{H\text{-}H}$ = 8.0 Hz, $^4J_{H\text{-}H}$ = 1.2 Hz), 6.70 (dd, 6H, H8(e) or H10(c), H8’(e’) or H10’(c’), $^3J_{H\text{-}H}$ = 8.0 Hz, $^4J_{H\text{-}H}$ = 1.2 Hz), 6.63 (dd, 6H, H8(e) or H10(c), H8’(e’) or H10’(c’), $^3J_{H\text{-}H}$ = 8.0 Hz, $^4J_{H\text{-}H}$ = 1.2 Hz), 6.51 (t, 6H, H9(d) or H9’(d’), $^3J_{H\text{-}H}$= 8.0 Hz), 6.40 (t, 6H, H9(d) or H9’(d’), $^3J_{H\text{-}H}$= 8.0 Hz).

^{1}H NMR (400 MHz, CD_3OD, 298 K): δ 9.14 (v br s, 12H, H4(b+b’)), 8.34 (br s, 12H, H2(a+a’)), 6.79 (br m, 12H, H8(e+e’) or H10(c+c’)), 6.49 (br t, 12H, H9(d+d’), $^3J_{H\text{-}H}$= 8.0 Hz).

^{1}H NMR (400 MHz, CD_3OD, 238 K): δ 9.31–9.60 (v br s, 12H, H4(b+b’)), 8.29–8.37 (v br s, 12H, H2(a+a’)), 6.45–6.93 (several br m, 12H, H8(e+e’) + H9(d+d’) + H10(c+c’)).

^{13}C{^{1}H} NMR (125.6 MHz, D_2O, 298 K): δ 153.4 (C7 or C7’), 152.6 (C7 or C7’), 145.8 (C1), 145.6 (C6 or C6’), 145.6 (C6 or C6’), 144.3 (C1’), 131.0 (C3), 130.7 (C3’), 123.7 (CH4(b)), 122.4 (C5 or C5’), 122.3 (CH2), 121.8 (C5 or C5’), 121.7 (CH4’(b’)), 121.1 (CH2’), 115.2 (CH9(d) or CH9’(d’)), 114.9 (CH9(d) or CH9’(d’)), 112.0 (CH8(e) or CH8’(e)’, CH10(c) or CH10’(c’)), 111.9 (CH8(e) or CH8’(e’), CH10(c) or CH10’(c’)), 111.5 (CH8(e) or CH8’(e’), CH10(c) or CH10’(c’)), 110.7 (CH8(e) or CH8’(e’), CH10(c) or CH10’(c’)).

IR (KBr/Nujol, cm^{-1}): ν(O-H) 3357 (v br); ν(C=N, C=C) 1576 (br), 1529.

HRMS-TOF-ESI (negative-ion detection, H_2O, ⧉$^{6-}$ = $[L_4Ga_6]^{6-}$, □$^{3-}$ = $[L_2Ga_3]^{3-}$): m/z found (calc.), [□$^{3-}$] = 467.6689 (467.6691); [□$^{3-}$ + K^+ + OH^-]$^{3-}$ = 486.3246 (486.3246); [□$^{3-}$ + 2 K^+ + 2 OH^-]$^{3-}$ = 504.9808 (504.9800); [□$^{3-}$ + 3 K^+ + 3 OH^-]$^{3-}$ = 523.6358 (523.6355); [□$^{3-}$ + K^+]$^{2-}$ = 720.9847 (720.9852); [⧉$^{6-}$ + 4 K^+ + 2 OH^-]$^{4-}$ = 749.9661 (749.9685); [□$^{3-}$ + 2 K^+ + OH^-]$^{2-}$ = 748.9681 (748.9684); [⧉$^{6-}$ + 5 K^+ + 2 OH^- + H^-]$^{4-}$ = 759.4660 (759.4669); [⧉$^{6-}$ + 5 K^+ + 2 OH^- + H^-]$^{4-}$ = 763.4597 (759.4604); [□$^{3-}$ + 3 K^+ + 2 OH^-]$^{2-}$ = 776.9530 (776.9516); [□$^{3-}$ + 4 K^+ + 3 OH^-]$^{2-}$ = 804.9348 (804.9381); [⧉$^{6-}$ + 4 K^+ + 3 OH^- + 2 Na^+]$^{3-}$ = 1020.2828 (1020.2856); [⧉$^{6-}$ + 5 K^+ + 3 OH^- + 1 Na^+]$^{3-}$ = 1025.6077 (1025.6102); [⧉$^{6-}$ + 6 K^+ + 3 OH^-]$^{3-}$ = 1030.9328 (1030.9328); [⧉$^{6-}$ + 5 K^+ + 3 OH^- + 2 Na^+]$^{2-}$ = 1549.9055 (1549.9099); [⧉$^{6-}$ + 6 K^+ + 3 OH^- + 1 Na^+]$^{2-}$ = 1557.8929 (1557.8969); [⧉$^{6-}$ + 7 K^+ + 3 OH^-]$^{2-}$ = 1562.8806 (1557.8838).

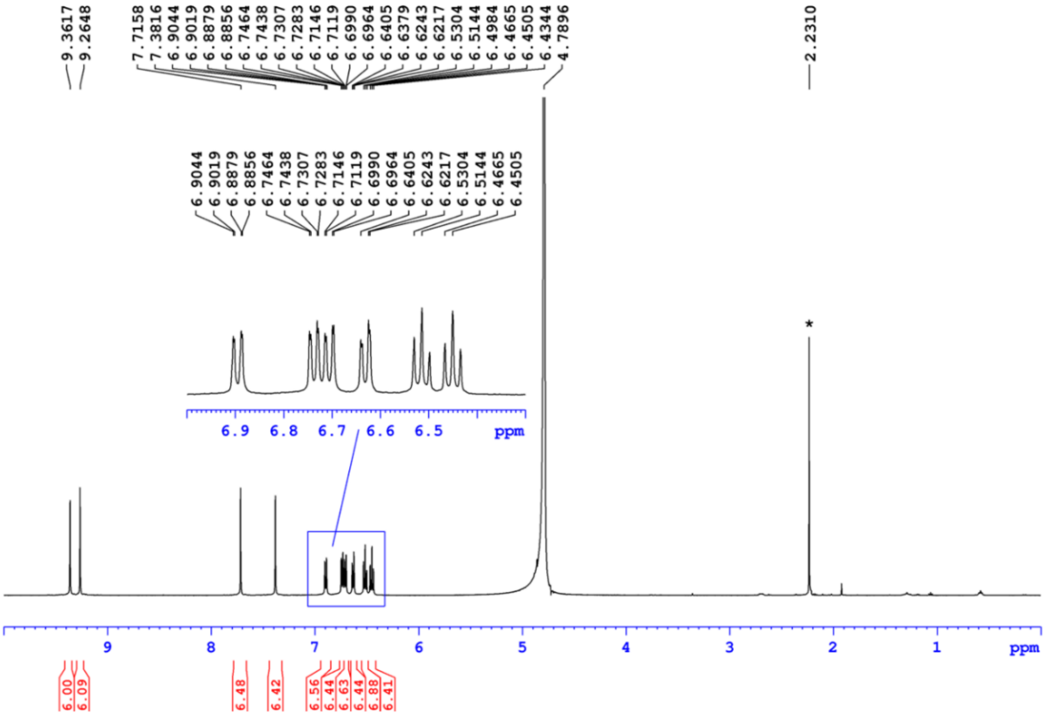

Figure S32. ^1H NMR (500 MHz, D_2O, 298 K) spectrum of **IC2**. The symbol * denotes small residual signal for acetone from crystallization and isolation.

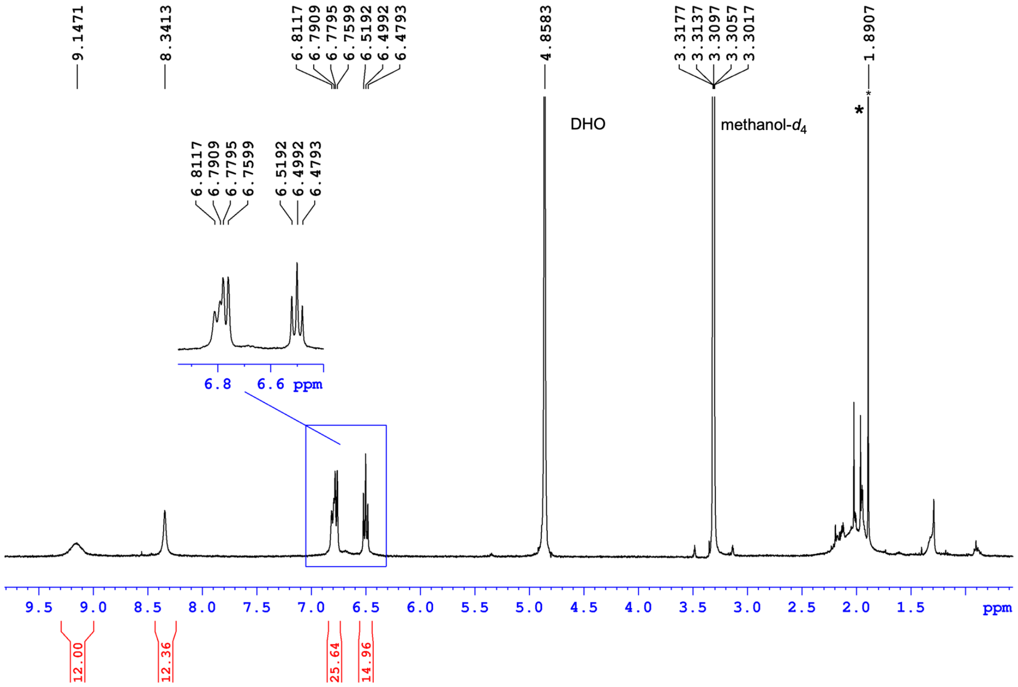

Figure S33. ^1H NMR (400 MHz, CD_3OD, 298 K) spectrum of **IC2**. The symbol * denotes small residual signal from acetylacetone from isolation.

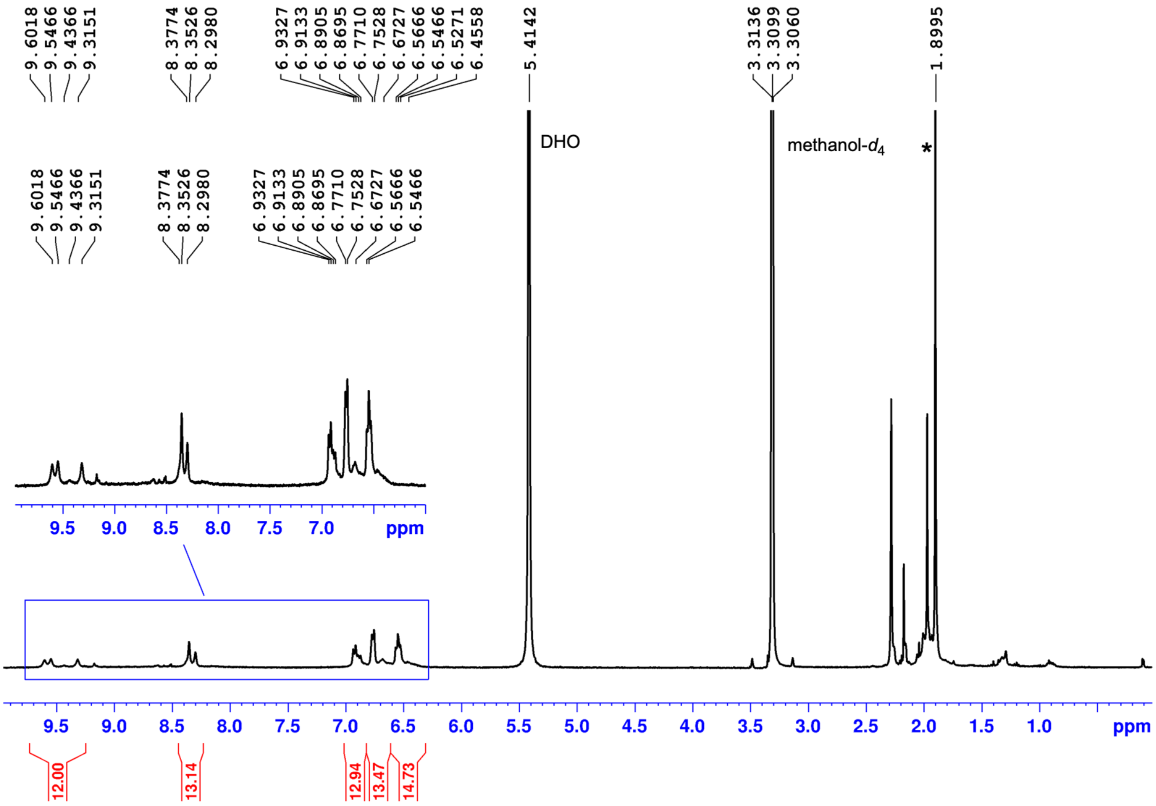

Figure S34. ^{1}H NMR (400 MHz, CD_3OD, 238 K) spectrum of **IC2**. The symbol * denotes small residual signal from acetylacetone from isolation.

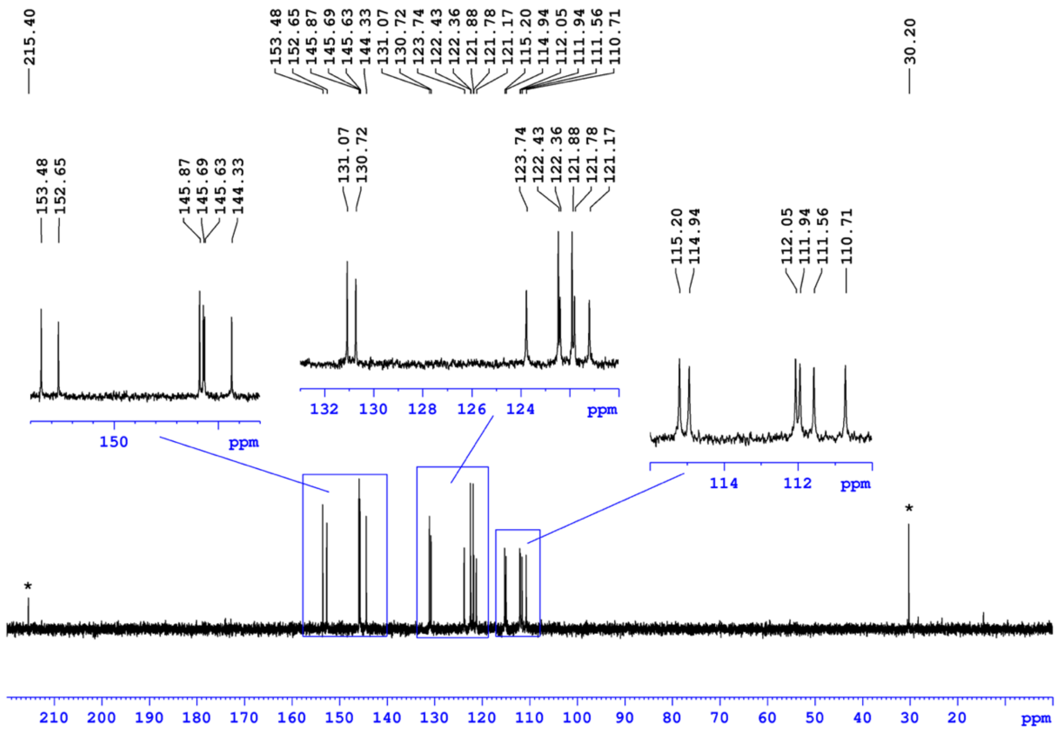

Figure S35. $^{13}C\{^{1}H\}$ NMR (125.6 MHz, D_2O, 298 K) spectrum of **IC2**. The symbol * denotes small residual signal for acetone from crystallization and isolation.

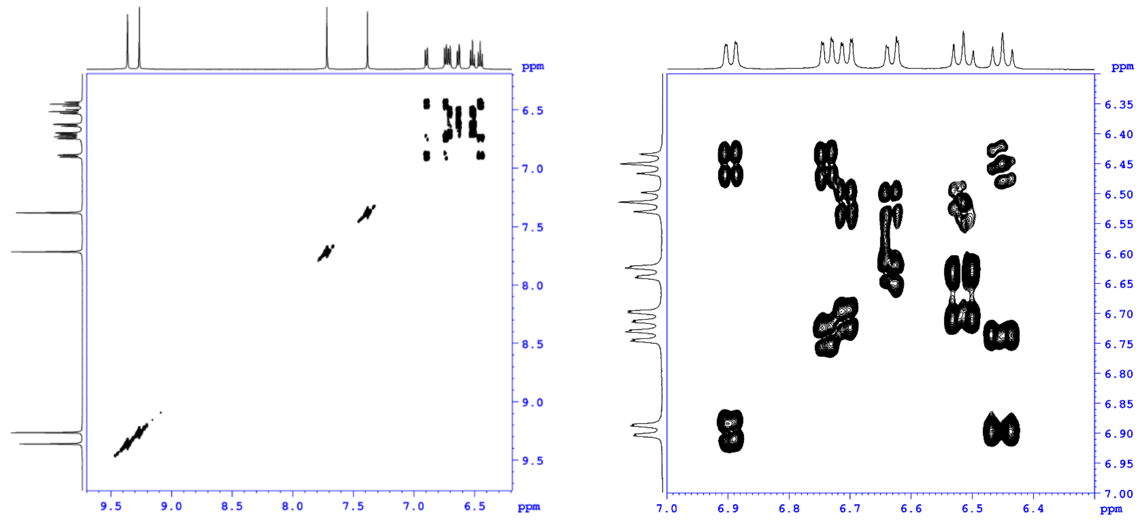

Figure S36. Selected sections for the $^{1}H,^{1}H$-COSY NMR (500 MHz, D_2O, 298 K) spectrum of **IC2**.

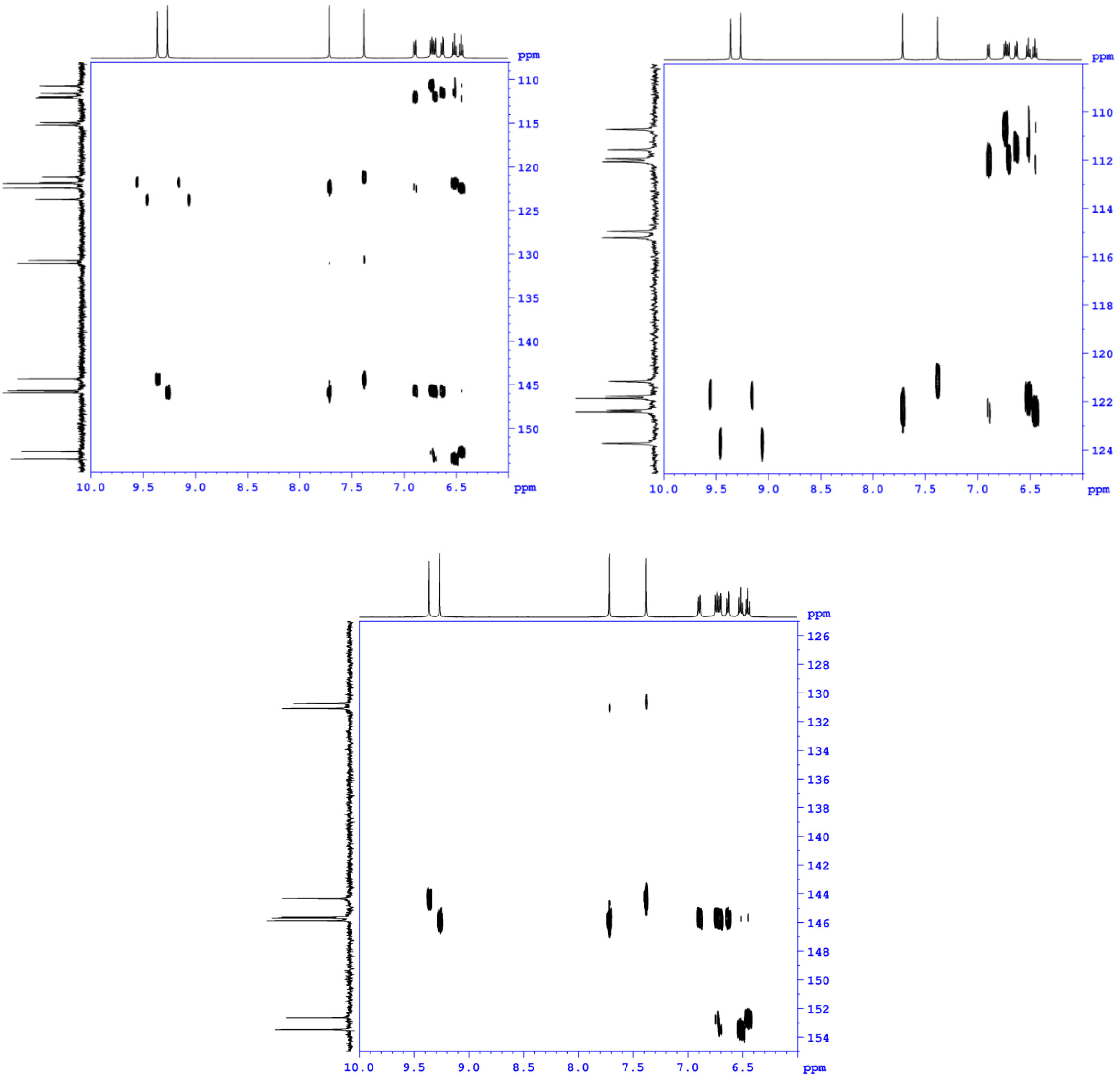

Figure S37. Sections for the $^{1}H,^{13}C$-HMBC (500 MHz, D_2O, 298 K) spectrum of **IC2**.

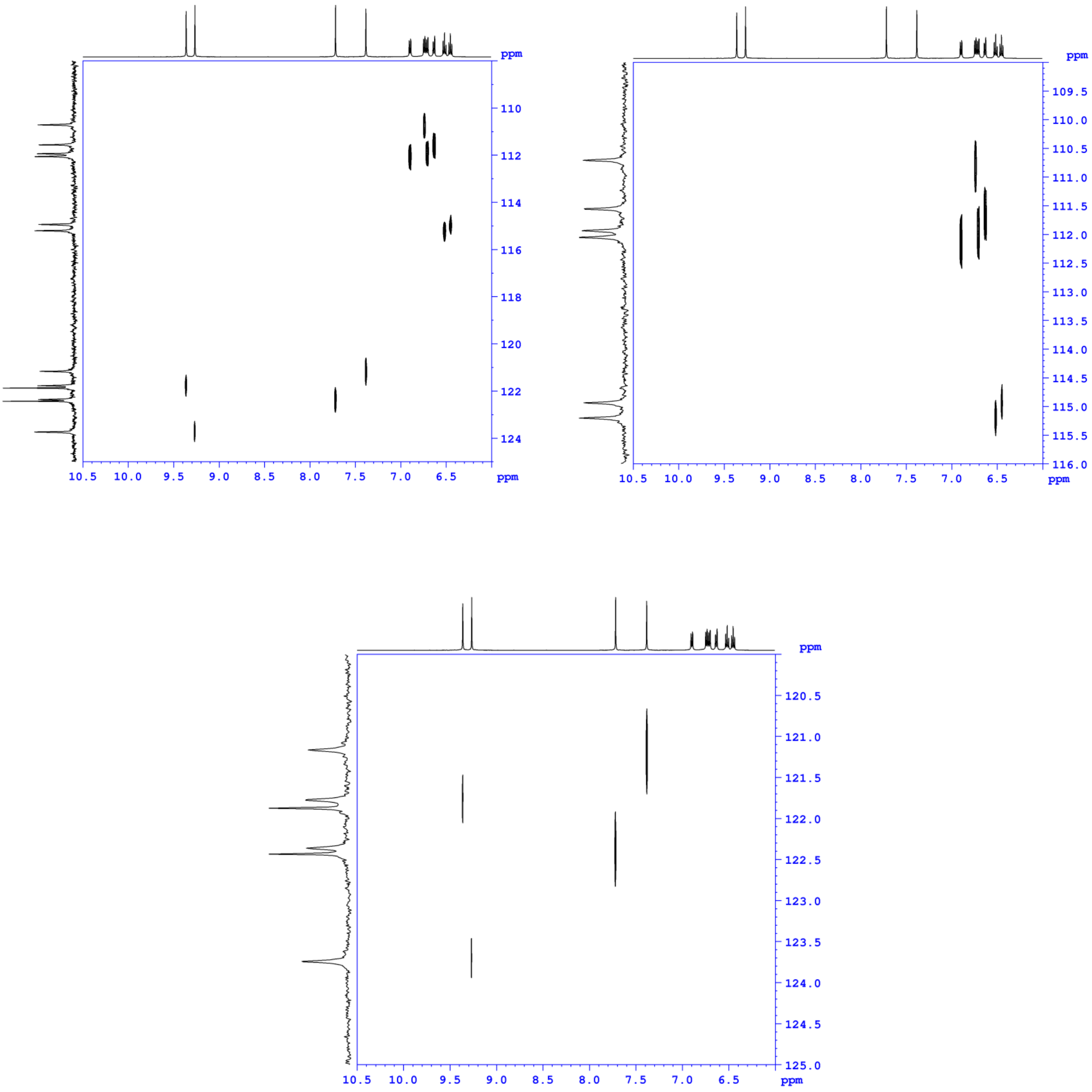

Figure S38. Sections for the $^{1}H,^{13}C$-HSQC (500 MHz, D_2O, 298 K) spectrum of **IC2**.

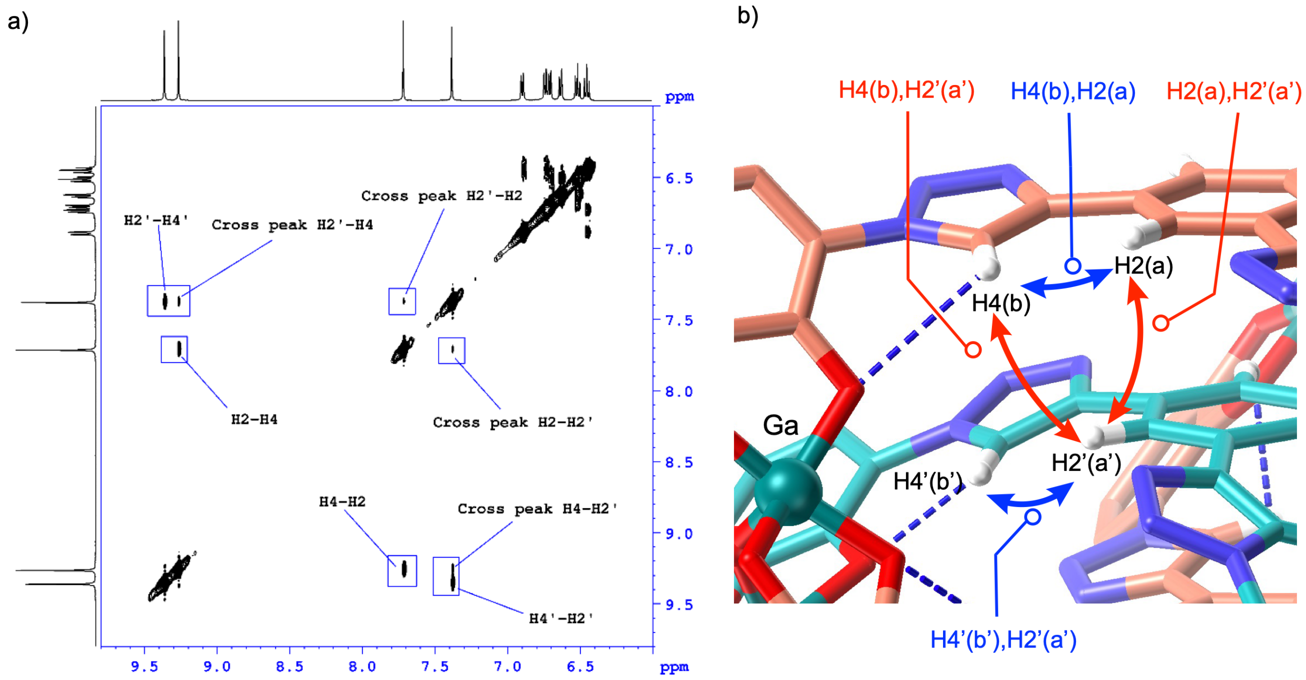

Figure S39. ^{1}H,^{1}H-NOESY spectrum (500 MHz, D_2O, 298 K) of **IC2**. NOE couplings are highlighted with a blue box. The highlighted cross peaks H4(b)-H2'(a') and H2(a)-H2'(a')/H2'(a')-H2(a) indicate ^{1}H atoms that are close between the inner and outer ligand decks (ie. they do not belong to the same ligand system) within the supramolecular molecular geometry of **IC2**. b) Illustration of NOE interactions in the crystalline structure of **IC2.**

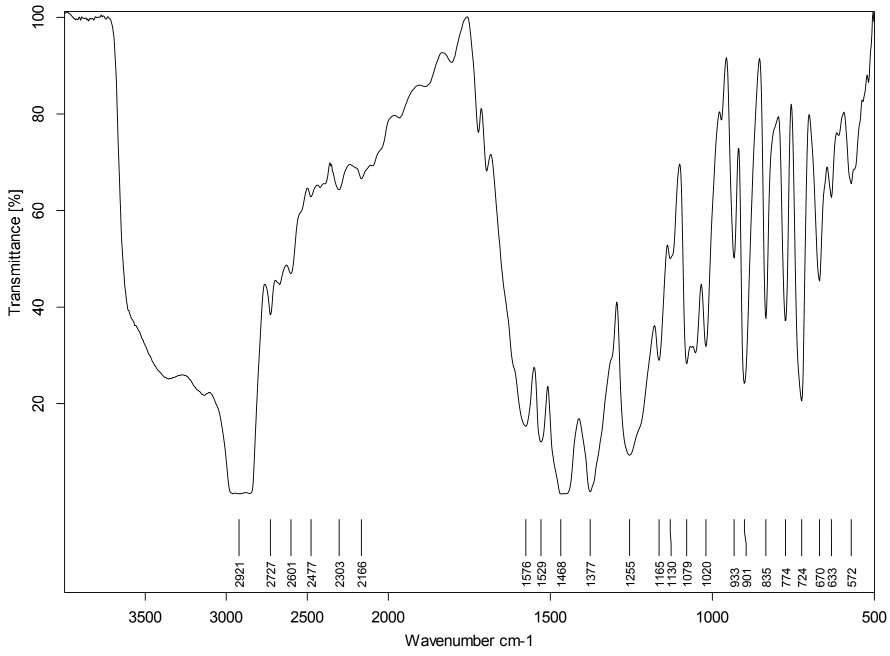

Figure S40. IR (KBr/Nujol, cm^{-1}) spectrum of **IC2**.

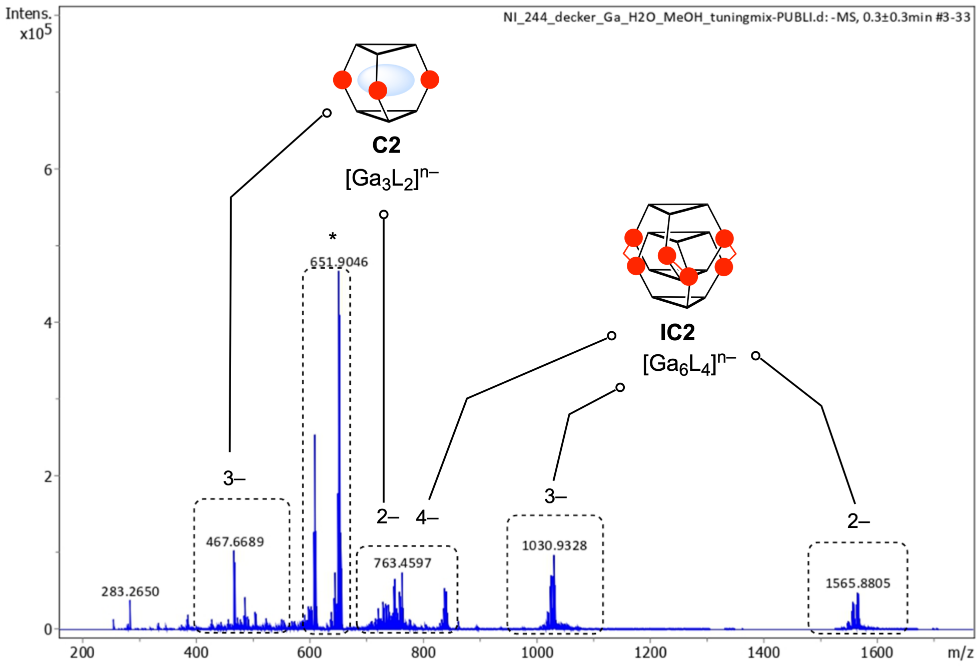

Figure S41. Full view for the HRMS-ESI (negative-ion detection mode) of **IC2**. Selected sections highlighted with a dashed box are those areas of interest that were fully studied in detail. (*) Several ions were detected in the range of m/z 600-700 for different aggregates resulting from fragmentation of the ligand $\mathbf{L}^{6-}$ with different ions under the mass spectrometry ionization conditions.

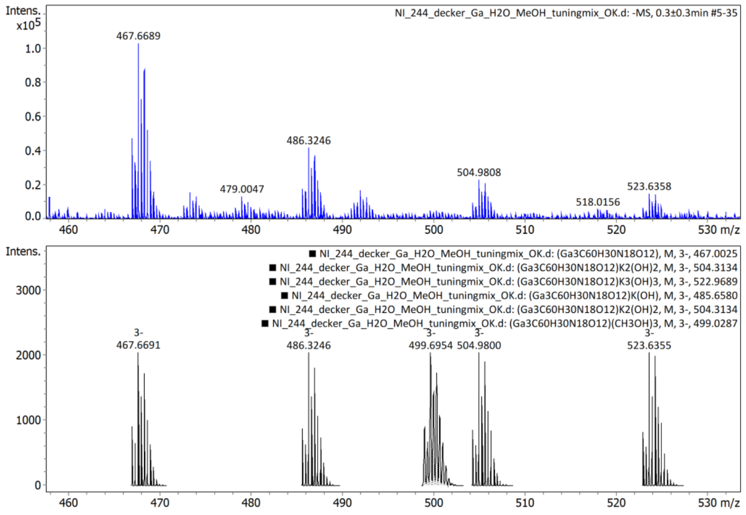

Figure S42. Section of the HRMS-ESI (negative-ion detection mode) for $\mathbf{[Ga_6L_6(OH)_3]K_9}$. Top: experimental; bottom: calculated.

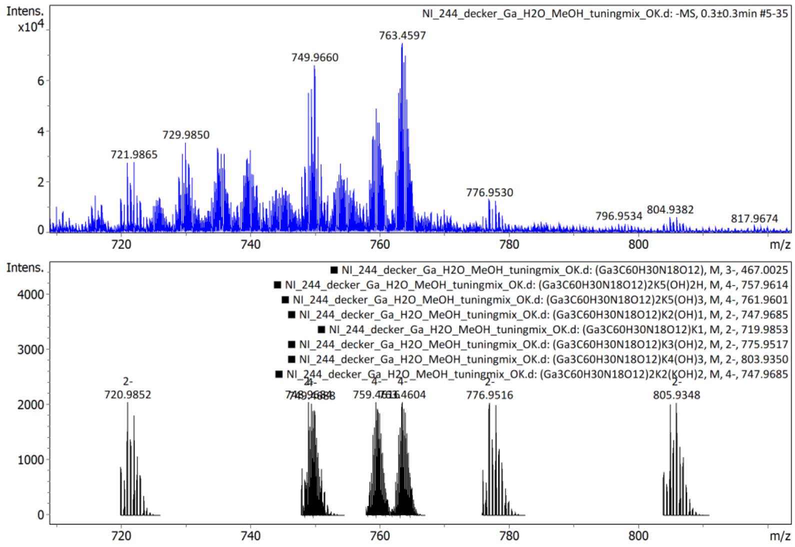

Figure S43. Section of the HRMS-ESI (negative-ion detection mode) for **$[Ga_6L_4(OH)_3]K_9$**. Top: experimental; bottom: calculated.

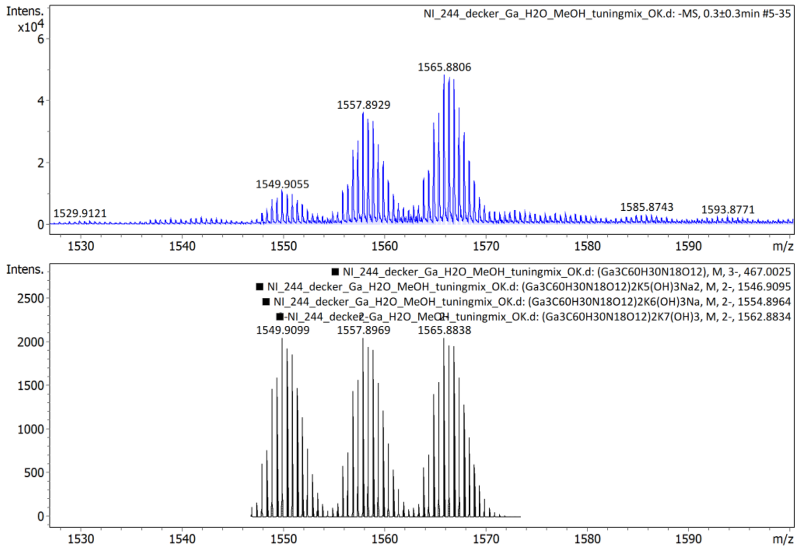

Figure S44. Section of the HRMS-ESI (negative-ion detection mode) for **$[Ga_6L_4(OH)_3]K_9$**. Top: experimental; bottom: calculated.

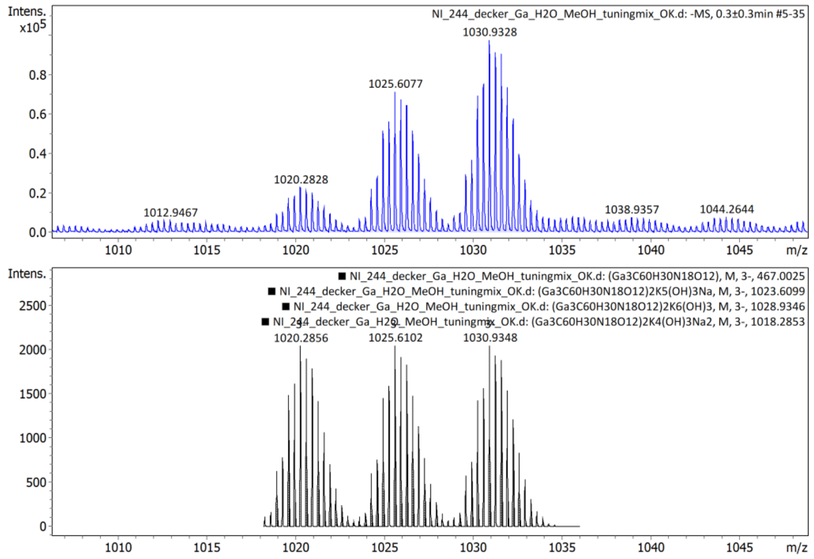

Figure S45. Section of the HRMS-ESI (negative-ion detection mode) for **[Ga$_6$L$_4$(OH)$_3$]K$_9$**. Top: experimental; bottom: calculated.

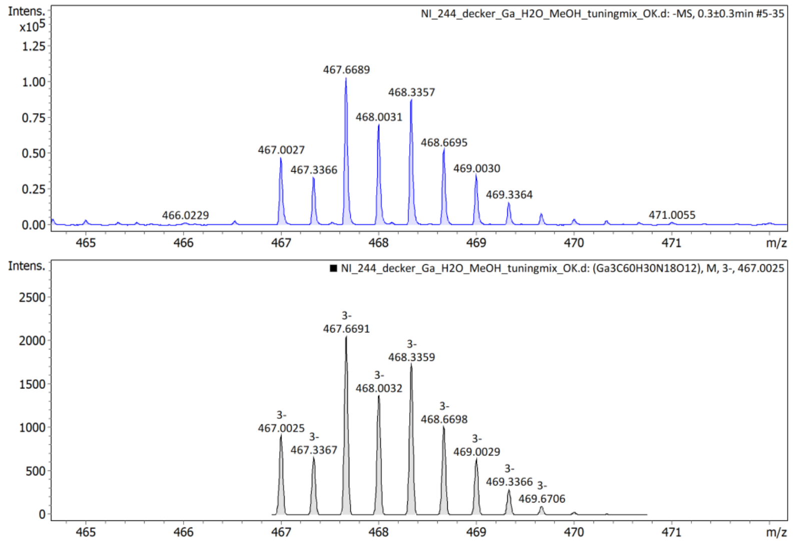

Figure S46. Section of the HRMS-ESI (negative-ion detection mode) corresponding to the species **[Ga$_3$L$_2$]$^{3–}$**. Top: experimental; bottom: calculated.

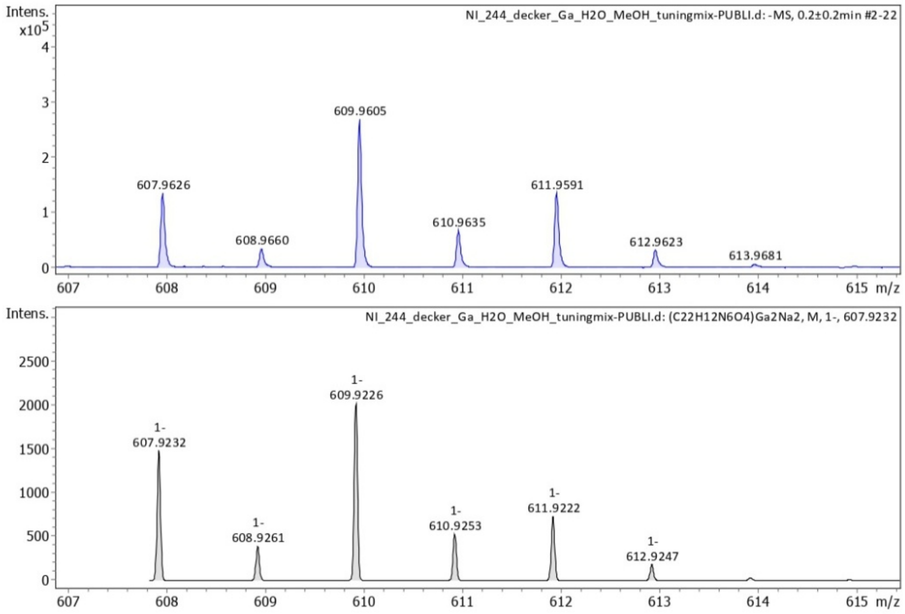

Figure S47. Section of the HRMS-ESI (negative-ion detection mode) corresponding to fragmentation of the ligand **L^{6-}** generating the species **[$C_{22}H_{12}N_6O_4Ga_2Na_2$]$^-$**. Top: experimental; bottom: calculated.

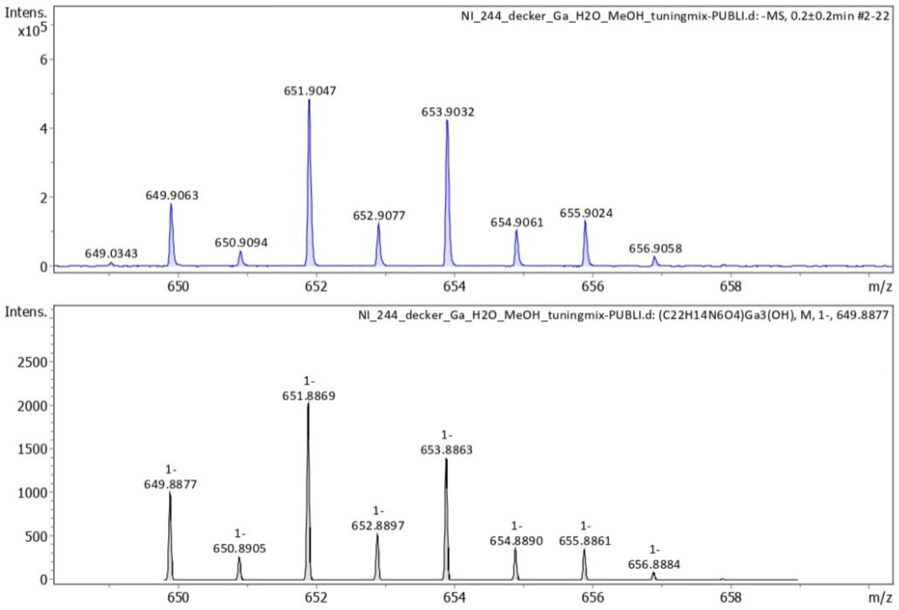

Figure S48. Section of the HRMS-ESI (negative-ion detection mode) corresponding to fragmentation of the ligand **L^{6-}** generating the species **[$C_{22}H_{14}N_6O_4Ga_3(OH)$]$^-$**. Top: experimental; bottom: calculated.

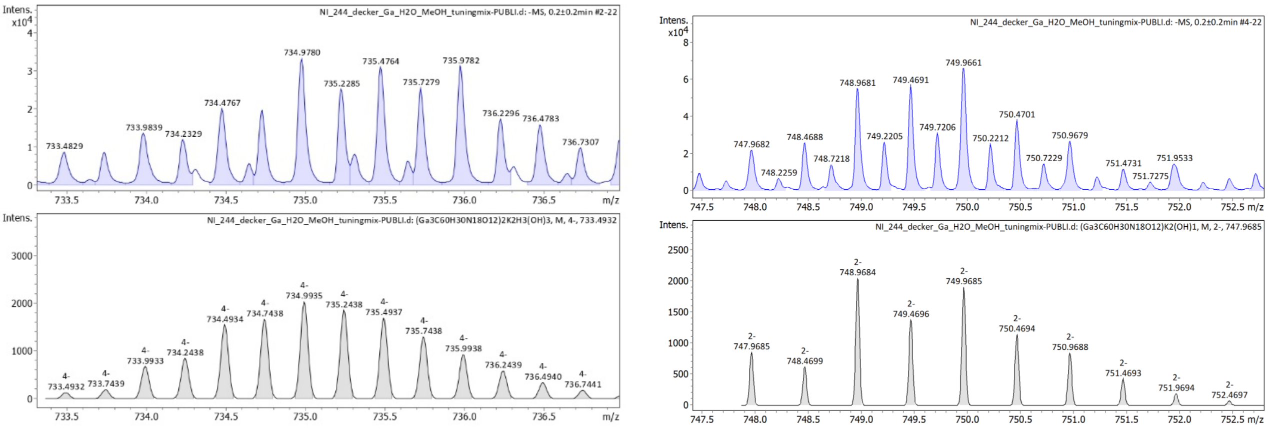

Figure S49. Sections of the high-resolution mass spectra (ESI) corresponding to the species **$[Ga_6L_4(OH)_3]^{9-}$ + $2K^+$ + $3H^+$** (left) and **$[Ga_3L_2(OH)_1]^{4-}$ + $2K^+$** (right). Top: experimental; bottom: calculated.

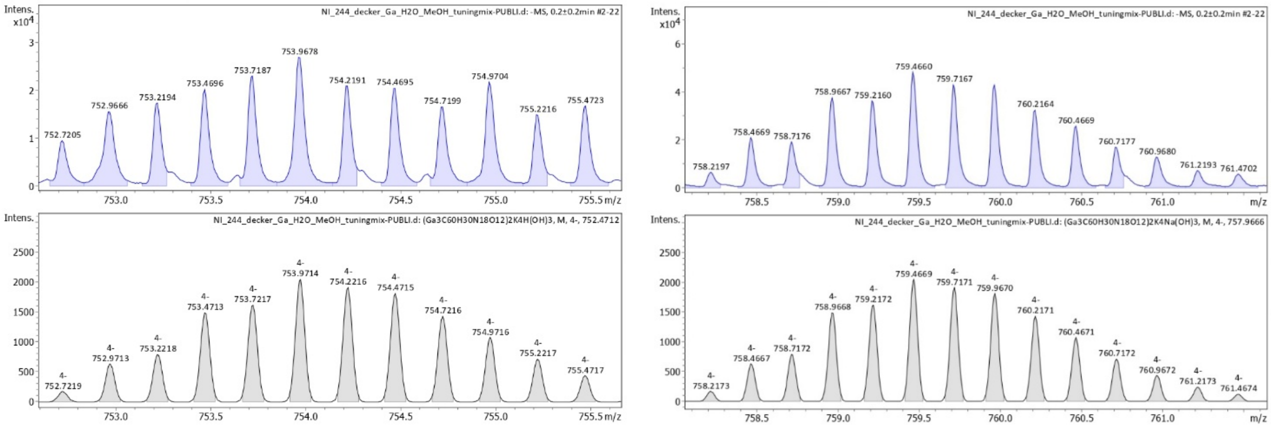

Figure S50. Sections of the high-resolution mass spectra (ESI) corresponding to the species **$[Ga_6L_4(OH)_3]^{9-}$ + $4K^+$ + H^+** (left) **$[Ga_6L_4(OH)_3]^{9-}$ + $4K^+$ + Na^+** (right). Top: experimental; bottom: calculated.

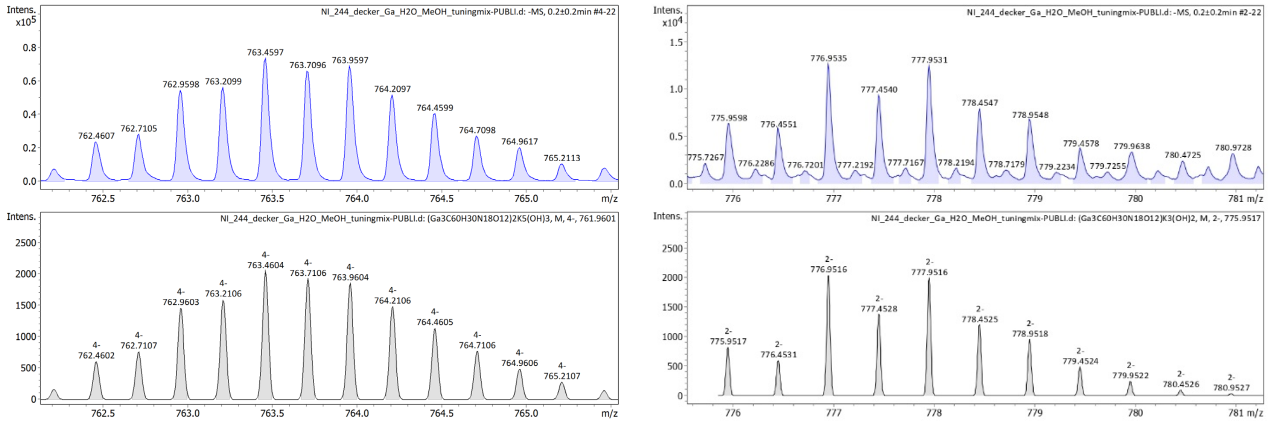

Figure S51. Sections of the high-resolution mass spectra (ESI) corresponding to the species **$[Ga_6L_4(OH)_3]^{9-}$ + 5 K^+** (left) and **$[Ga_3L_2(OH)_2]^{5-}$ + 3K^+** (right). Top: experimental; bottom: calculated.

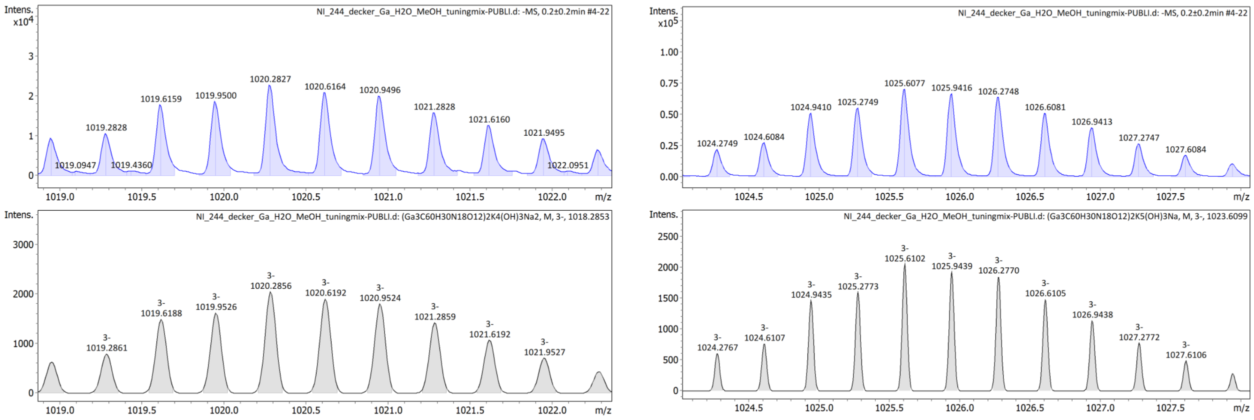

Figure S52. Sections of the high-resolution mass spectra (ESI) corresponding to the species **$[Ga_6L_4(OH)_3]^{9-}$ + 4 K^++ 2 Na^+** (left) and **$[Ga_6L_4(OH)_3]^{9-}$ + 5 K^++ Na^+** (right). Top: experimental; bottom: calculated.

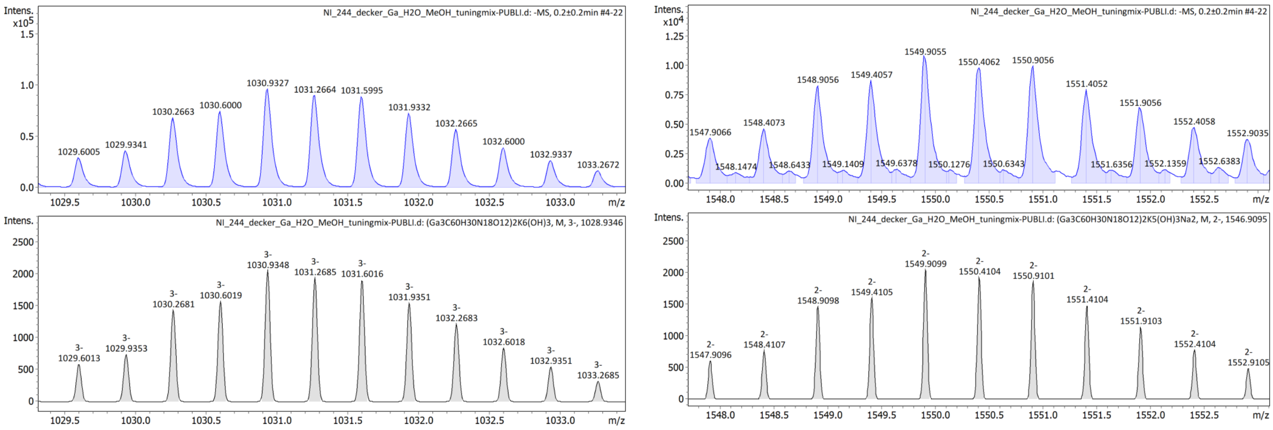

Figure S53. Sections of the high-resolution mass spectra (ESI) corresponding to the species **$[Ga_6L_4(OH)_3]^{9-}$ + 6 K^+**(left) and **$[Ga_6L_4(OH)_3]^{9-}$ + 5 K^+ + 2 Na^+**(right). Top: experimental; bottom: calculated.

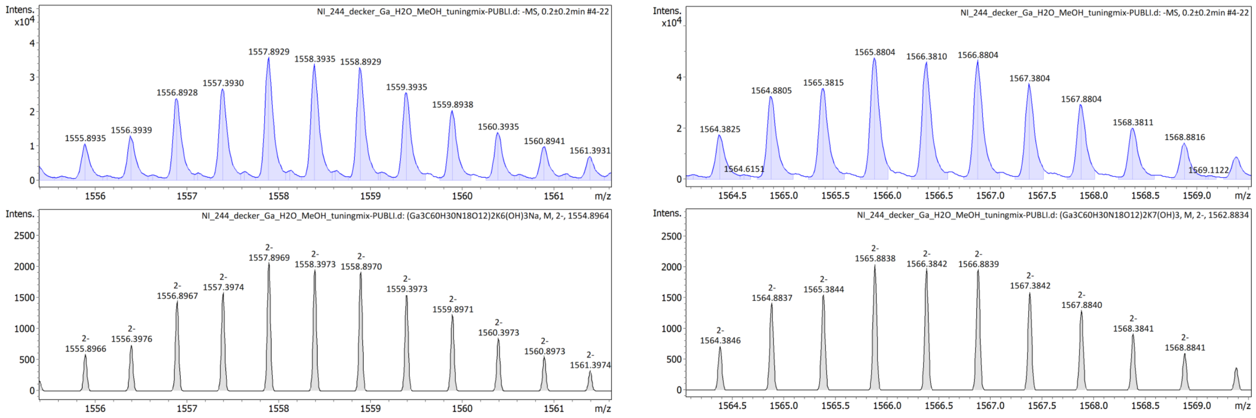

Figure S54. Sections of the high-resolution mass spectra (ESI) corresponding to the species **$[Ga_6L_4(OH)_3]^{9-}$ + 6K$^+$ + Na$^+$** (left) and **$[Ga_6L_4(OH)_3]^{9-}$ + 7 K$^+$** (right). Top: experimental; bottom: calculated.

S4 Variable Temperature (VT) NMR Spectroscopic Studies

S4.1 ^{1}H VT NMR Spectroscopic Study of IC1 in CD_3OD (238–318 K)

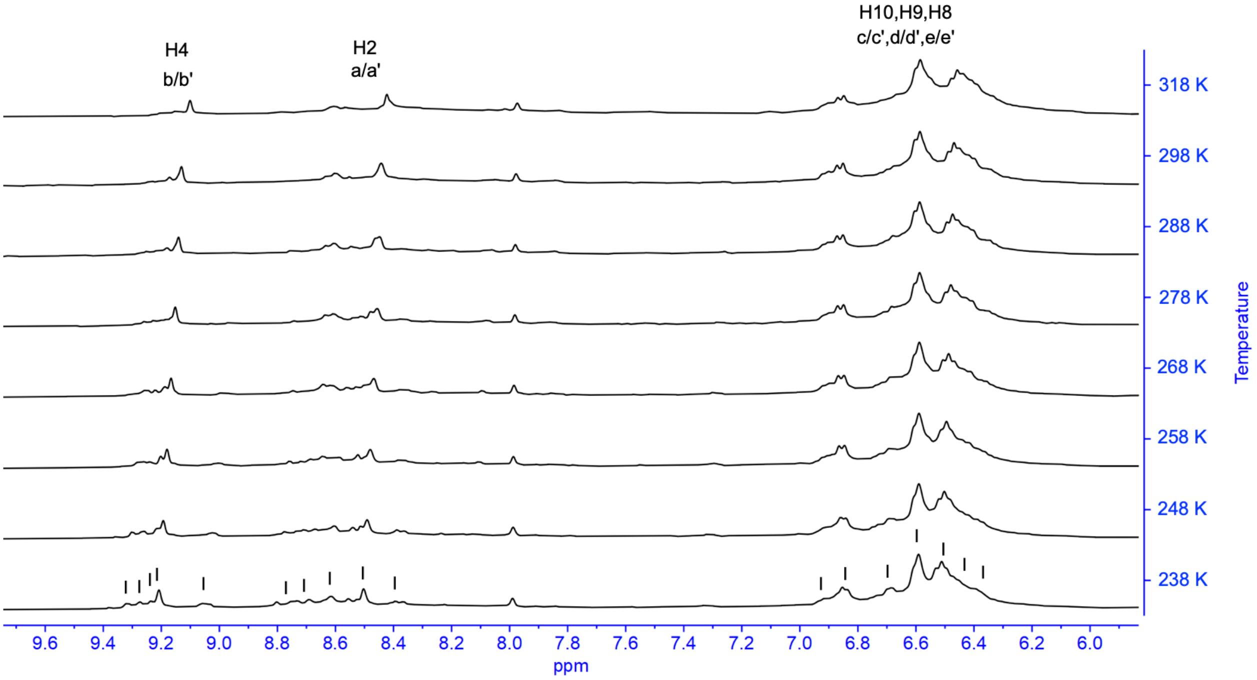

Figure S55. ^{1}H VT NMR spectroscopy analysis (400 MHz, CD_3OD, 238-318 K) of **IC1**.

S4.2 ^{1}H VT NMR Spectroscopic Study of IC2 in CD_3OD (238–318 K)

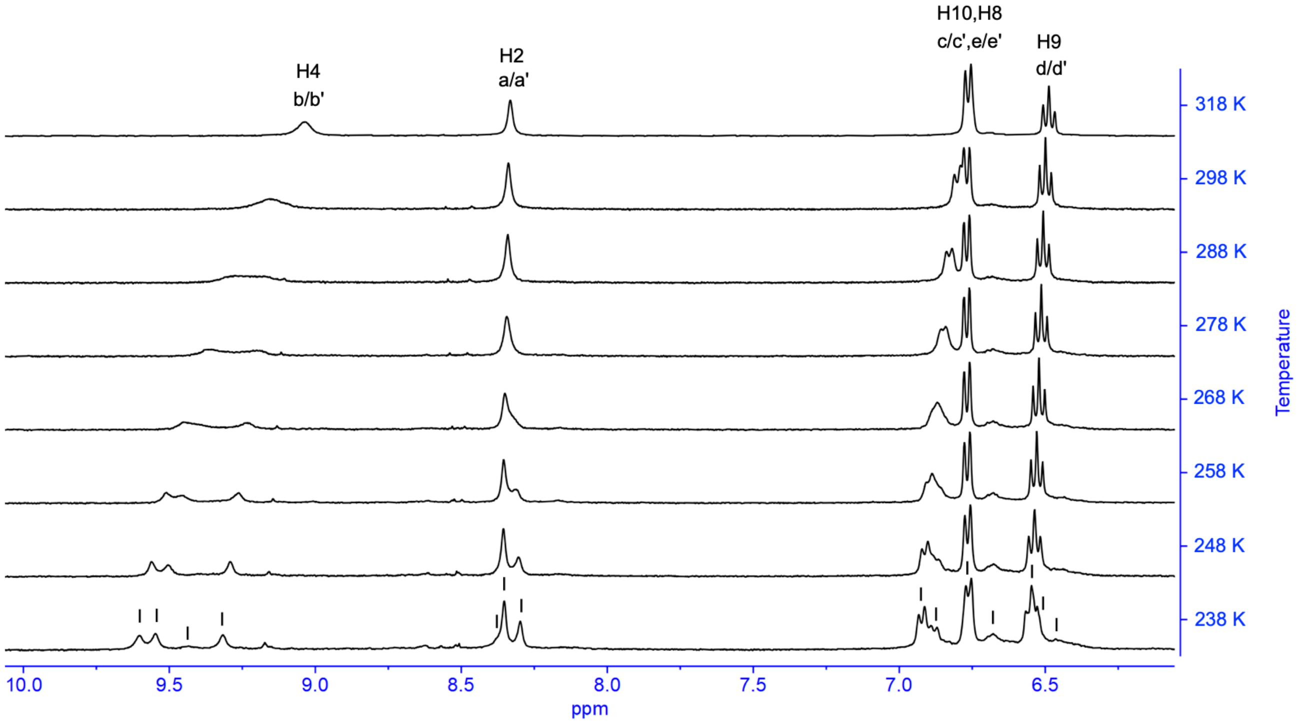

Figure S56. ^{1}H VT NMR spectroscopy analysis (400 MHz, CD_3OD, 238-318 K) of **IC2**.

S5 Diffusion Ordered NMR Spectroscopic (DOSY) studies

S5.1 DOSY NMR general information

The Diffusion-Ordered Spectroscopy (DOSY) NMR experiments were performed on either a Bruker AVIIIHD 9.4 tesla nanobay (400 MHz for 1H) or Bruker AVIII 11.7 tesla (500 MHz for 1H, equipped with a Prodigy cryoprobe) spectrometer at 298 K under TopSpin (version 4.1, Bruker Biospin, Karlsruhe). Both spectrometers were equipped with a BBFO-z-atm probe and an actively shielded z-gradient coil capable of delivering a maximum gradient strength of 54 G cm^{-1}. DOSY data were acquired using the Bruker pulse program *dstegp3s*, which employs a double stimulated echo with three spoiling gradients. Sine-shaped gradient pulses were used with a duration of 4 ms and a diffusion period of 100 ms. Gradient recovery delays of 200 μs followed each gradient pulse. Data were systematically accumulated by linearly varying the diffusion encoding gradients from 2% to 95% of maximum strength over 64 increments. The signal decay dimension of the pseudo-2D data was generated by Fourier transformation. DOSY plots were processed using the DOSY module within Topspin. Parameters were optimized empirically to achieve optimal data quality for presentation. Diffusion coefficients (D) were obtained by fitting signal intensities to the Stejskal-Tanner equation. An estimate of the hydrodynamic diameter (d_H) of the diffusing species was calculated using the Stokes-Einstein equation, r_H =

$K_BT/3\eta\pi D$, were D is the diffusion coefficient ($m^2 \cdot s^{-1}$), π is 3.1415926, K_B is Boltzmann's constant (1.380649×10^{-23} $J \cdot K^{-1}$), T is the absolute temperature (298 K), and η is the solvent viscosity at 298 K (e.g., 1.25×10^{-3} Pa·s for D_2O, and 0.796×10^{-3} Pa·s for DMF-d_7).[3] According to this equation the hydrodynamic radius (r_H) of the diffusing species in solution is defined as $r_H = K_BT/6\eta\pi D$ (m). Calibration curves validated this methodology by plotting *log*(D) versus *log*(r_H), demonstrating excellent agreement between crystallographic and solution-derived radii values.

S5.2 Study of solvent and aggregation effects

The free ligands **Me_6L** and **H_6L** were found to be insoluble in D_2O, with **H_6L** soluble DMSO-d_6 and **Me_6L** in $CDCl_3$. DMF-d_7 was therefore chosen for ligand DOSY because it dissolves both compounds, being a strong hydrogen-bond acceptor, mimics the H-bonding capability of D_2O. Conversely, the supramolecular interlocked cages **IC1** and **IC2** are soluble and stable in D_2O, not stable in DMF-d_7, precluding a solvent-matched experiment. Importantly, since viscosity (η) is explicitly accounted for by the Stokes-Einstein equation, the observed radii reflect intrinsic aggregation and structural effects rather than solvent viscosity differences. The measured hydrodynamic radii of **Me_6L** (9 Å) and **H_6L** (11 Å) in DMF-d_7 substantially exceeded their estimated crystallographic radius (6 Å) clearly indicating significant ligand–ligand aggregation via π–π stacking and hydrogen bonding interactions, effects also prominently observed in the crystal structure of **Me_6L**. Such aggregation-driven pre-organization provides a plausible structural basis for efficient ligand self-assembly into the quadruple-decker-type supramolecular interlocked cages **IC1** and **IC2**.

In CD_3OD, **IC1** and **IC2** show coalesced, higher-symmetry ^1H NMR patterns relative to D_2O. We ascribe this to faster interconversion of inner and outer ligand decks and/or reduced inner/outer Δδ in CD_3OD, where hydrophobic stacking is weaker and protic solvation lowers exchange barriers. On cooling in CD_3OD, the broad averaged ligand signals at 298 K progressively resolve and desymmetrize as exchange slows. At 238 K, Ha/Ha', Hb/Hb′, and also the catechol Hc/Hc', Hd/Hd' and He/He', split into multiple resonances, which restores to the average state upon warming up. These low-temperature spectra are consistent with freezing of the interlocked topologies, in which the four ligands are non-equivalent, akin the non-equivalent ligand environment observed in the crystal structure of IC2 (none of the protons are equivalent). In D_2O, stronger hydrophobic/solvophobic stabilization slows this exchange and yields the resolved duplication used for assignment.

DOSY experiments were employed to monitor the formation of supramolecular cages **IC1** and **IC2**. The significantly lower diffusion coefficients observed for **IC1** (1.37×10^{-10} m^2/s) and **IC2** (1.58×10^{-10} m^2/s) in D_2O compared to their ligand precursors **Me_6L** (3.10×10^{-10} m^2/s) and **H_6L** (2.6×10^{-10} m^2/s) in DMF-d_7 clearly confirmed the successful self-assembly into larger supramolecular species. The resulting hydrodynamic radii from DOSY (r_H^{DOSY} of 13 Å and 11 Å for **IC1** and **IC2**, respectively) closely matched the compact, crystallographically determined cage dimensions (13 Å), demonstrating structural

integrity and stability of these cages in aqueous solution. Consistent with this, the resulting r_H^{DOSY} values (**IC1** of 13 Å; **IC2** of 12 Å in CD_3OD) match the compact crystallographic dimensions, supporting intact cage architectures in aqueous solution.

S5.3 DOSY NMR studies of ligands

S5.3.1 ^{1}H-DOSY NMR study of **Me₆L**

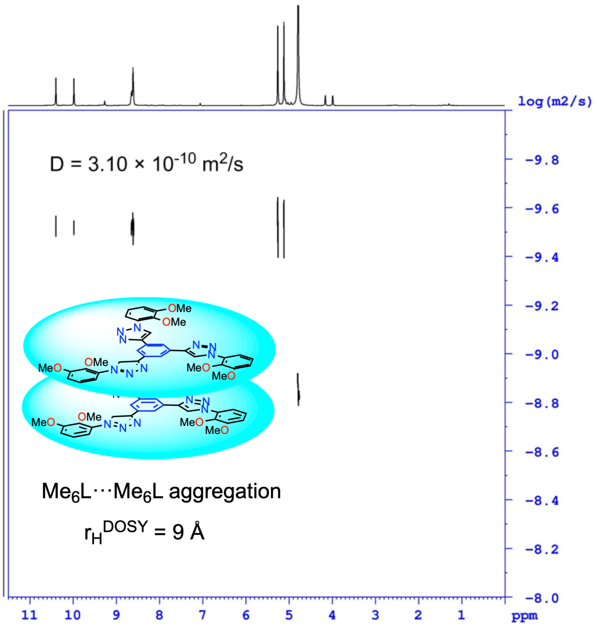

Figure S57. ^{1}H DOSY NMR (400 MHz) of **Me_6L** in DMF-d_7 at 298 K.

Table S1. D (m^2s^{-1}) and r_H^{DOSY} for species found in a solution of **Me_6L** in DMF-d_7 at 298 K.

Species	D (m^2s^{-1})	r_H^{DOSY} (Å), from DOSY	$r_H^{X\text{-}ray}$ (Å), estimate from crystal structure
**Me_6L**	3.10×10^{-10}	9	6

Comments on the ^{1}H DOSY NMR spectrum of Me_6L in DMF-d_7 solution: The DOSY NMR spectrum of **Me_6L** in DMF-d_7 reveals a single diffusing species, with a measured diffusion coefficient of 3.10×10^{-10} m^2/s. The resulting hydrodynamic radius (r_H^{DOSY} = 9 Å), obtained using the Stokes-Einstein equation, is significantly larger than the crystallographically estimated radius (6 Å). The crystallographic radius was calculated by approximating the molecule as a triangular prism (triangle sides 12, 14, and 18 Å, thickness 5 Å), then converting its volume into an equivalent spherical radius. The marked difference between DOSY-derived and crystallographic radii predominantly arises from ligand–ligand aggregation, driven by π–π stacking and hydrogen bonding interactions. Such aggregation is directly supported by prominent intermolecular contacts observed in the crystal structure of **Me_6L**, indicating a pronounced intrinsic tendency of this ligand to self-associate in solution.

S5.3.2 ^{1}H-DOSY NMR study of H_6L

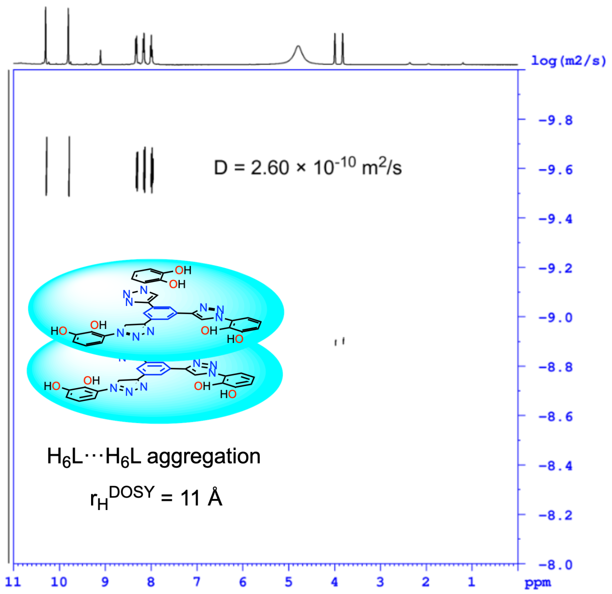

Figure S58. ^{1}H DOSY NMR (400 MHz) of H_6L in DMF-d_7 at 298 K.

Table S2. D (m^2s^{-1}) and r_H^{DOSY} for species found in a solution of H_6L in DMF-d_7 at 298 K.

Species	D (m^2s^{-1})	r_H^{DOSY} (Å), from DOSY	$r_H^{X\text{-}ray}$ (Å), estimate from crystal structure
**H_6L**	2.60×10^{-10}	11	--

Comments on the ^{1}H DOSY NMR spectrum of H_6L in DMF-d_7 solution: The DOSY NMR spectrum of **H_6L** in DMF-d_7 shows a single diffusing species with a diffusion coefficient of 2.60×10^{-10} $m^2 \cdot s^{-1}$. The calculated hydrodynamic radius (r_H^{DOSY} = 11 Å) is significantly larger compared to the structurally analogous ligand **Me_6L** (r_H^{DOSY} = 9 Å). The increased hydrodynamic radius observed for **H_6L** primarily indicates enhanced ligand-ligand aggregation, driven by strong intermolecular hydrogen bonding facilitated by its hydroxyl (OH) substituents, as well as potential π–π stacking interactions analogous to those clearly observed in **H_6L**. Such pronounced aggregation effects suggest that **H_6L** exhibits even greater intrinsic pre-organization in solution than **Me_6L**.

S5.4 DOSY NMR studies of coordination cages

S5.4.1 ^{1}H-DOSY NMR study of **IC1** in D_2O

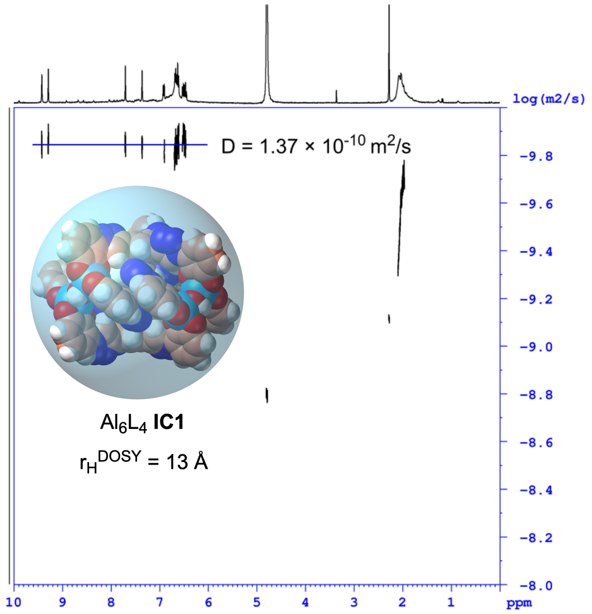

Figure S59. ^{1}H DOSY NMR (400 MHz) of **IC1** in D_2O at 298 K.

Table S3. D (m^2s^{-1}) and r_H^{DOSY} for species found in a solution of **IC1** in D_2O at 298 K.

Species	D (m^2s^{-1})	r_H^{DOSY} (Å), from DOSY	$r_H^{X\text{-}ray}$ (Å) estimate from crystal structure
**IC1**	1.37 × 10^{-10}	13	-

Comments on the ^{1}H DOSY NMR spectrum of IC1 in D_2O solution: The DOSY spectrum of **IC1** in D_2O reveals a single diffusing species with a diffusion coefficient of 1.37 × 10^{-10} m^2/s, corresponding to a hydrodynamic radius of 13 Å. This radius closely matches the crystallographically determined radius (13 Å) from the structurally analogous gallium derivative **IC2** strongly supporting retention of the cage-like supramolecular structure **IC1** in aqueous solution. The significant decrease in diffusion coefficients, from ligand precursors **Me_6L** (3.10 × 10^{-10} m^2/s) and **H_6L** (2.60 × 10^{-10} m^2/s) measured in DMF-d_7 to the assembled interlocked **IC1** cage (1.37 × 10^{-10} m^2/s) provides clear evidence of supramolecular assembly into a substantially larger and more compact species. These DOSY results confirm successful formation and stability of the interlocked **IC1** supramolecular structure under aqueous conditions.

S5.4.2 ^{1}H-DOSY NMR study of **IC1** in CD_3OD

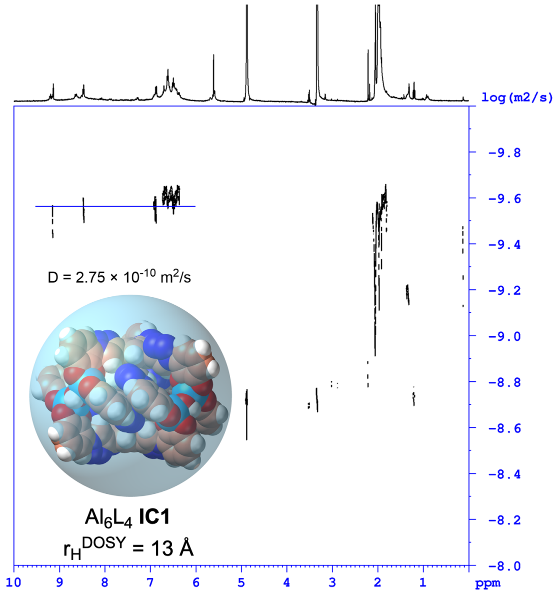

Figure S60. ^{1}H DOSY NMR (400 MHz) of **IC1** in CD_3OD at 298 K.

Table S4. D (m^2s^{-1}) and r_H^{DOSY} for species found in a solution of **IC1** in CD_3OD at 298 K.

Species	D (m^2s^{-1})	r_H^{DOSY} (Å), from DOSY	$r_H^{X\text{-}ray}$ (Å) estimate from crystal structure
**IC1**	2.75×10^{-10}	13	-

Comments on the ^{1}H DOSY NMR spectrum of IC1 in CD_3OD solution: The DOSY spectrum of **IC1** in CD_3OD reveals a single diffusing species with a diffusion coefficient of 2.75×10^{-10} m^2/s, corresponding to a hydrodynamic radius of 13 Å. This matches the same hydrodynamic radius obtained in D_2O and also the crystallographically determined radius (13 Å) from the structurally analogous gallium derivative **IC2**, strongly supporting retention of the cage-like supramolecular structure **IC1** in aqueous solution. The significant decrease in diffusion coefficients, from ligand precursors **Me_6L** (3.10×10^{-10} m^2/s) and **H_6L** (2.60×10^{-10} m^2/s) measured in DMF-d_7 to the assembled interlocked **IC1** cage (1.37×10^{-10} m^2/s) provides clear evidence of supramolecular assembly into a substantially larger and more compact species. These DOSY results confirm successful formation and stability of the interlocked **IC1** supramolecular structure under CD_3OD conditions.

S5.4.3 ^{1}H-DOSY NMR study of **IC2** in D_2O

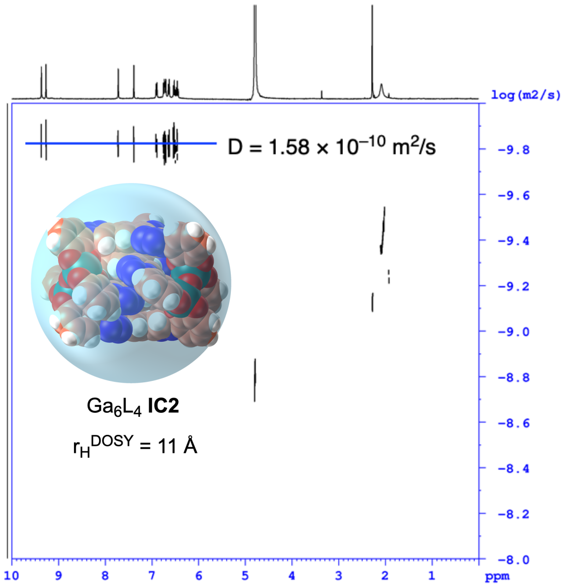

Figure S61. ^{1}H DOSY NMR (400 MHz) of **IC2** in D_2O at 298 K.

Table S5. D (m^2s^{-1}) and r_H^{DOSY} for species found in a solution of **IC2** in D_2O at 298 K.

Species	D (m^2s^{-1})	r_H^{DOSY}, from DOSY(Å)	$R_{X\text{-}ray}$ (Å), estimate from crystal structure of **IC2**
**IC2**	1.58 × 10^{-10}	11	13

Comments on the ^{1}H DOSY NMR spectrum of $[Ga_6L_4(OH)_3]K_9$ in D_2O solution: The DOSY spectrum of **IC2** in D_2O shows a single diffusing species with a diffusion coefficient of 1.58 × 10^{-10} m^2/s. This experimentally determined hydrodynamic radius closely matches the crystallographically established radius (13 Å), strongly indicating retention of the cage-like supramolecular structure in aqueous solution. The notable decrease in diffusion coefficient from ligand precursors **Me$_6$L** (3.1 × 10^{-10} m^2/s) and **H$_6$L** (2.6 × 10^{-10} m^2/s) in DMF-d_7 to the assembled interlocked **IC2** cage in D_2O (1.58 × 10^{-10} m^2/s) clearly demonstrates successful aggregation into a substantially larger and compact supramolecular species. These DOSY results robustly confirm the integrity and stability of the **IC2** supramolecular structure in aqueous solution.

S5.4.4 ^{1}H-DOSY NMR study of **IC2** in CD_3OD

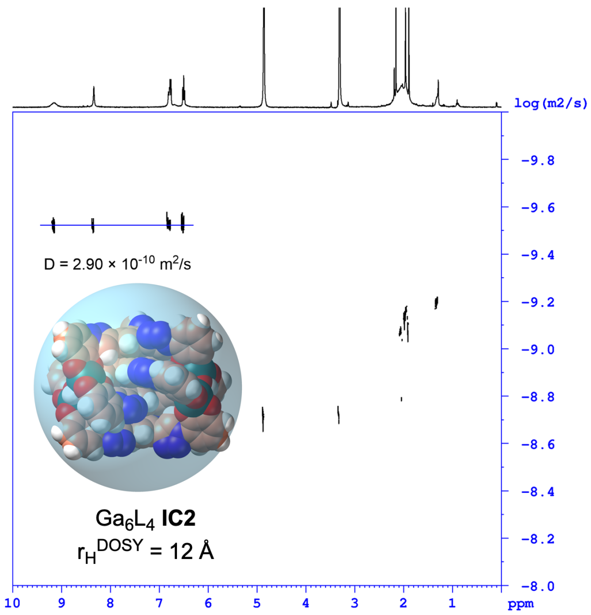

Figure S62. ^{1}H DOSY NMR (400 MHz) of **IC2** in CD_3OD at 298 K.

Table S6. D (m^2s^{-1}) and r_H^{DOSY} for species found in a solution of **IC2** in CD_3OD at 298 K.

Species	D (m^2s^{-1})	r_H^{DOSY}, from DOSY(Å)	$R_{X\text{-}ray}$ (Å), estimate from crystal structure of **IC2**
**IC2**	2.90×10^{-10}	12	13

Comments on the ^{1}H DOSY NMR spectrum of [$Ga_6L_4(OH)_3$]K_9 in CD_3OD solution: The DOSY spectrum of **IC2** in CD_3OD shows a single diffusing species with a diffusion coefficient of 2.90×10^{-10} m^2/s. This experimentally determined hydrodynamic radius of 12 Å closely matches the crystallographically established radius (13 Å), strongly indicating retention of the cage-like supramolecular structure in aqueous solution. The notable decrease in diffusion coefficient from ligand precursors **Me$_6$L** (3.1×10^{-10} m^2/s) and **H$_6$L** (2.6×10^{-10} m^2/s) in DMF-d_7 to the assembled interlocked **IC2** cage in CD_3OD (2.90×10^{-10} m^2/s) clearly demonstrates successful aggregation into a substantially larger and compact supramolecular species. These DOSY results robustly confirm the integrity and stability of the **IC2** supramolecular structure in CD_3OD solution.

S5.4.5 Details of cage hydrodynamic raii study

Estimations of the hydrodynamic radii (r_H) of the interlocked assemblies **IC1** and **IC2** were calculated using geometrically centered spherical probes of the given radii (Å) within space-filling models, utilizing van der Waals radii for all atoms as reported by S. Álvarez.[4] The spherical probe was fitted to the smallest dimension, representing the hydrodynamic diameter typically observed in DOSY NMR experiments for spheric-like molecules. This method provides a practical approximation of the hydrodynamic diameter in solution, correlating well with the experimentally observed diffusion behavior.

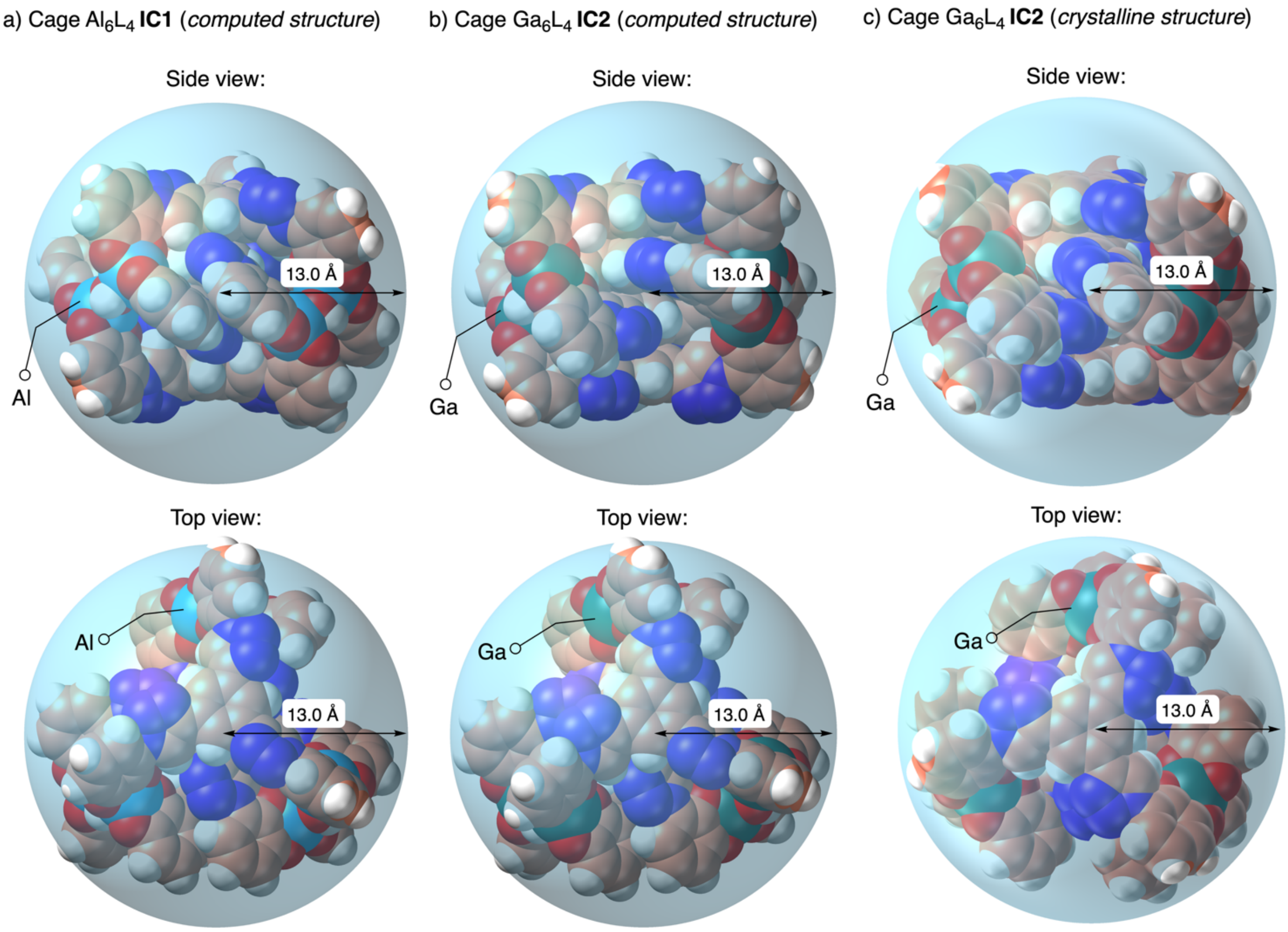

Figure S63. Estimated molecular radii (r_H, Å) for the interlocked cage Al_6L_4 **IC1** and Ga_6L_4 **IC2**: a) cage Al_6L_4 **IC1** (13 Å) obtained from the electronic computed structure (see Section 9), b) cage Ga_6L_4 **IC2** (13 Å) obtained from the electronic computed structure (see Section 9), c) cage Ga_6L_4 **IC2** (13 Å) obtained from the crystalline structural data.

S6 NMR studies of the disassembly–reassembly of IC1 and IC2

S6.1 In-situ NMR titration monitoring of disassembly–reassembly of IC1 with DCl–NaOD

General procedure: A solution of **IC1** (6.1 mg, 2.06 μmol) was prepared in D_2O (0.5 mL) in a 5 mm NMR tube. The initial ^1H NMR spectrum, serving as a baseline, was recorded at time zero (t = 0) (see **Figure S64**). To monitor the self-assembly process, sequential additions of deuterium chloride (DCl) from a stock solution (126.7 mM in D_2O) were performed. Specifically, aliquots of 16 μL (corresponding to 2.06 μmol) were added up to four times (1–4 equiv), with thorough mixing followed by immediate ^1H NMR measurement after each addition, maintaining a consistent 2-minute interval between the additions. Observations showed a progressive disappearance of the signals for **IC1** with each DCl addition, culminating in a complete signal loss upon reaching 3 equivalents of DCl (see **Figure S64**). No further changes were observed after adding a fourth equivalent of DCl. This resulted in the emergence of broad signals corresponding to a dissociated aggregation, identified as $[Al_2(H_xL)_2]$, comprising partially protonated linkers (H_xL^{n-}, n < 6), Al^{3+}, and K^+ counterions, resulting from complete dissociation of **IC1**.

Subsequently, to assess the reversibility of this self-assembly process, sodium deuteroxide (NaOD) was incrementally added to the system, following the same procedure (16 μL aliquots from a 126.7 mM stock solution in D_2O, corresponding to 2.06 μmol each addition, up to four equivalents). With each addition of NaOD, the broad signals attributed to the disassembled $[Al_2(H_xL)_2]$ aggregation state gradually disappeared, while the distinct, sharp signals characteristic of the reassembled **IC1** complex re-emerged, confirming the reversible nature of the self-assembly process.

The formation of NaCl and KCl, consequential to the process, did not impact the self-assembly dynamics. This indicates that the presence of these salts does not interfere with the self-assembly process, a detail that underscores the robustness of the **IC1** system in maintaining its structural integrity even in the presence of potential ionic perturbations. These self-assembly experiments were performed several times from independent synthesis of **IC1** giving reproducible results each time.

These conditions were fully reproducible under the concentration range 1–5 mM, due to the solubility limitations of **IC1**, showing two identical ^1H NMR set splitting across the range, with no resonances attributable to monomeric Al_3L_2 species. Thus, the interlocked topologies **IC1** persist well in the concentration range 1–5 mM. Experiments at 4 mM concentration are shown.

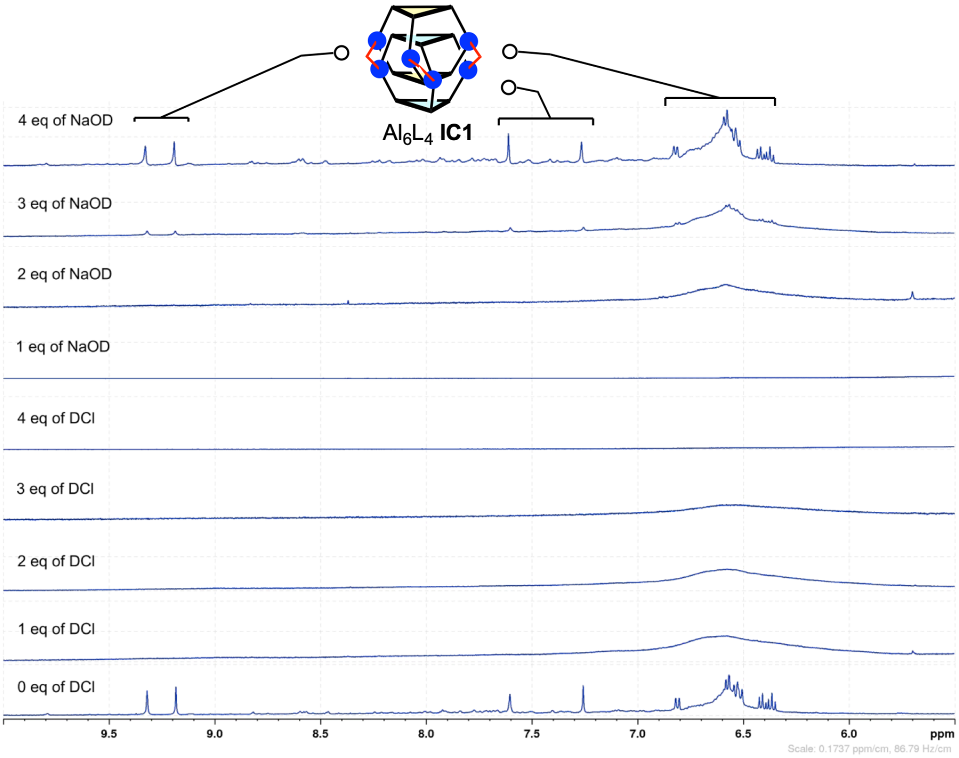

Figure S64. Stacked ^{1}H NMR (500 MHz, D_2O, 298 K, 4 mM) spectra illustrating the reversible self-assembly of **IC1**. The spectra reveal the disassembly with sequential additions of DCl (1–4 equiv), followed by its stepwise reassembly upon subsequent additions of NaOD (1–4 equiv). Spectra were recorded 2 minutes after each addition, showcasing the swift temporal dynamics of the disassembly–reassembly process.

S6.2 In-situ NMR titration monitoring of disassembly–reassembly of IC2 with DCl–NaOD

General procedure: A solution of **IC2** (5.7 mg, 1.78 μmol) was prepared in D_2O (0.5 mL) in a 5 mm NMR tube. The initial ^{1}H NMR spectrum, serving as a baseline, was recorded at time zero (t = 0) (see **Figure S65**). To monitor the self-assembly process, sequential additions of deuterium chloride (DCl) from a stock solution (126.7 mM in D_2O) were performed. Specifically, aliquots of 14 μL (corresponding to 1.78 μmol) were added up to four times (1–4 equiv), with thorough mixing followed by immediate ^{1}H NMR measurement after each addition, maintaining a consistent 2-minute interval between the additions. Observations showed a progressive disappearance of the signals for **IC2** with each DCl addition, culminating in a complete signal loss upon reaching 3 equivalents of DCl (see **Figure S65**). No further changes were observed after adding a fourth equivalent of DCl. This resulted in the emergence of broad signals corresponding to a dissociated aggregation, identified as $[Ga_2(H_xL)_2]$, comprising partially protonated linkers (H_xL^{n-}, n < 6), Ga^{3+}, and K^+ counterions, resulting from complete dissociation of **IC2**.

Subsequently, to assess the reversibility of this self-assembly process, sodium deuteroxide (NaOD) was incrementally added to the system, following the same procedure (14 µL aliquots from a 126.7 mM stock solution in D_2O, corresponding to 1.78 µmol each addition, up to four equivalents). With each addition of NaOD, the broad signals attributed to the disassembled $[Ga_2(H_xL)_2]$ aggregation state gradually disappeared, while the distinct, sharp signals characteristic of the reassembled **IC2** complex re-emerged, confirming the reversible nature of the self-assembly process.

The formation of NaCl and KCl, consequential to the process, did not impact the self-assembly dynamics. This indicates that the presence of these salts does not interfere with the self-assembly process, a detail that underscores the robustness of the **IC2** system in maintaining its structural integrity even in the presence of potential ionic perturbations. These self-assembly experiments were performed several times from independent synthesis of **IC2** giving reproducible results each time.

These conditions were fully reproducible under the concentration range 1–5 mM showing two identical 1H NMR set splitting across the range, with no resonances attributable to monomeric Ga_3L_2 species. Thus, the interlocked topologies **IC2** persist well in the concentration range 1–5 mM. Experiments at 4 mM concentration are shown.

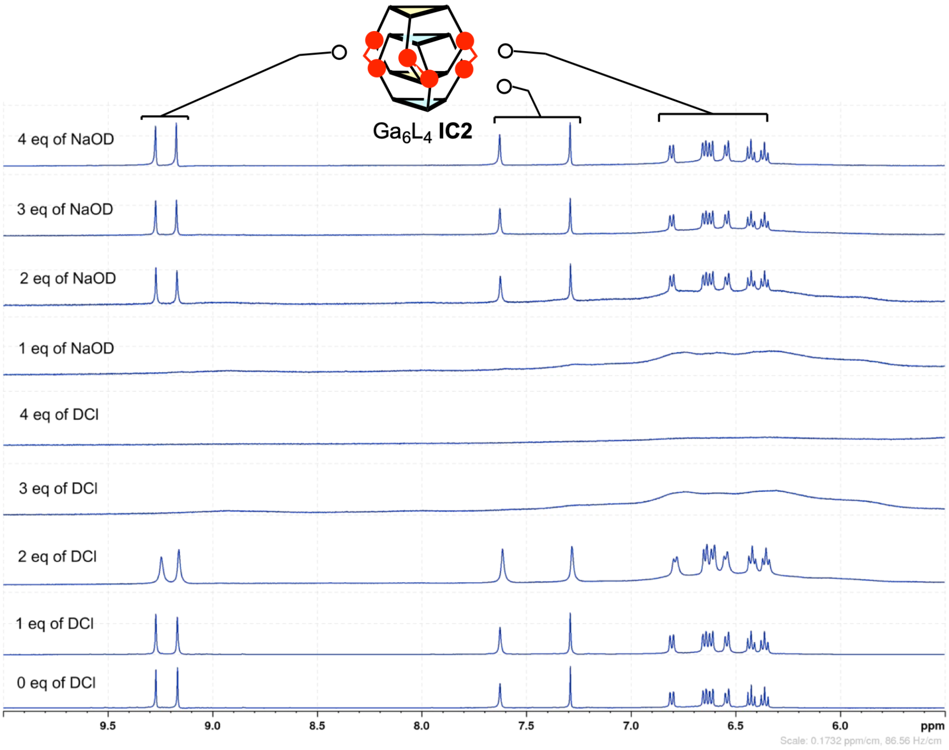

Figure S65. Stacked ^1H NMR (500 MHz, D_2O, 298 K, 4 mM) spectra illustrating the reversible self-assembly of **IC2**. The spectra reveal the disassembly with sequential additions of DCl (1–4 equiv), followed by its stepwise reassembly upon subsequent additions of NaOD (1–4 equiv). Spectra were recorded 2 minutes after each addition, showcasing the swift temporal dynamics of the assembly-disassembly process.

S6.3 In-situ NMR monitoring of assembly of IC1 via titration with $AlCl_3$

General Procedure: H_6L (5.4 mg, 8.95 μmol) in D_2O (0.5 mL) in a 5 mm NMR tube. To deprotonate **H_6L**, NaOD (212μL from a 0.2534 M stock solution in D_2O, 53.7 μmol) were added to the suspension, followed by immediate and thorough mixing resulting with a solution. Then, a ^1H NMR spectrum was measured to show complete deprotonation of **H_6L** to give **Na_6L**, serving as a baseline (t = 0). To monitor the self-assembly process, 2 equiv of $AlCl_3$ from a stock solution (0.2919 M in D_2O) were added. Specifically, an aliquot of 31.0 μL (corresponding to 8.95 μmol) was added (2 equiv), with thorough mixing followed by immediate ^1H NMR measurement after this addition, maintaining a consistent 2-minute interval after this addition (see **Figure S66**). Observations showed a re-appearance of the signals for **IC1** with each $AlCl_3$ addition. In contrast, to the same process with $GaCl_3$ to give **IC2** some broadness in the NMR was observed for **IC1** after the addition of $AlCl_3$. These self-assembly experiments were performed several times, giving reproducible results each time. These conditions were fully reproducible under the concentration range 1–5 mM, due to the solubility limitations of **IC1**, showing two identical. Experiments at 4 mM concentration are shown.

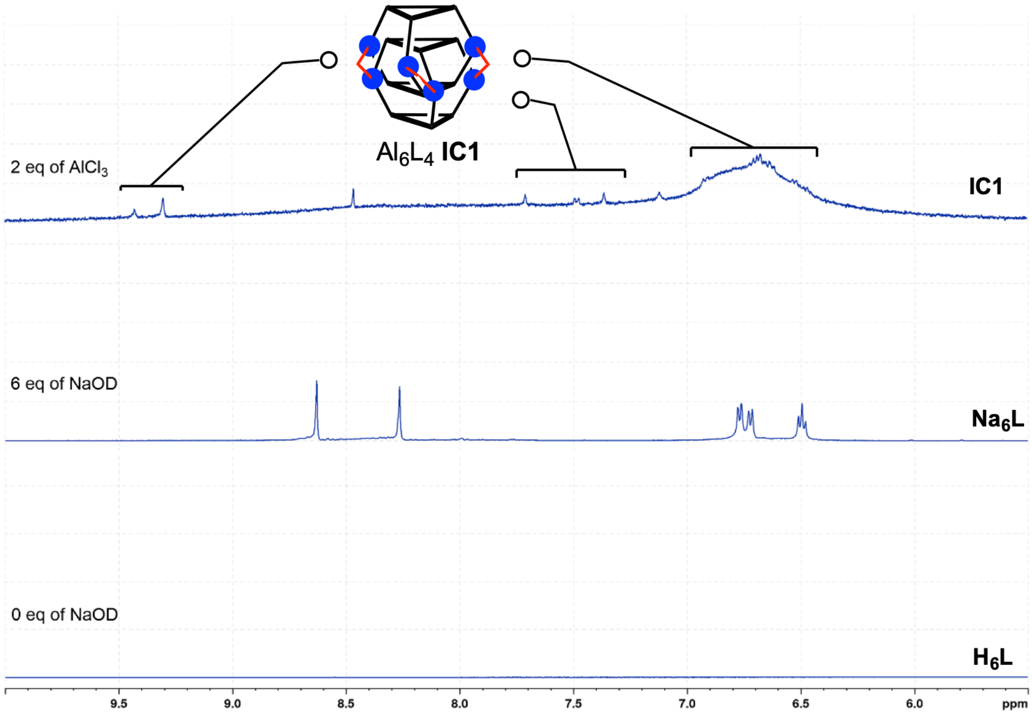

Figure S66. Stacked ^1H NMR (500 MHz, D_2O, 298 K, 4 mM) spectra illustrating the self-assembly of **IC1** via titration with $AlCl_3$ (2 equiv) of **L^{6-}**. Spectra were recorded 2 minutes after each addition, showcasing the swift temporal dynamics of the assembly process.

S6.4 In-situ NMR monitoring of assembly of IC2 via titration with $GaCl_3$

General Procedure: **H_6L** (5.1 mg, 8.45 μmol) in D_2O (0.5 mL) in a 5 mm NMR tube. To deprotonate **H_6L**, NaOD (396 μL from a 0.1267 M stock solution in D_2O, 50.70 μmol) were added to the suspension, followed by immediate and thorough mixing resulting with a solution. Then, a ^{1}H NMR spectrum was measured to show complete deprotonation of **H_6L** to give **Na_6L**, serving as a baseline (t = 0). To monitor the self-assembly process, 2 equiv of $GaCl_3$ from a stock solution (0.2542 M in D_2O) were added. Specifically, an aliquot of 33 μL (corresponding to 8.45 μmol) was added (2 equiv), with thorough mixing followed by immediate ^{1}H NMR measurement after this addition, maintaining a consistent 2-minute interval after this addition (see **Figure S67**). Observations showed a progressive re-appearance of the signals for **IC2** after the addition of $GaCl_3$ addition. These self-assembly experiments were performed several times, giving reproducible results each time. These conditions were fully reproducible under the concentration range 1–5 mM, due to the solubility limitations of **IC2**, showing two identical. Experiments at 4 mM concentration are shown

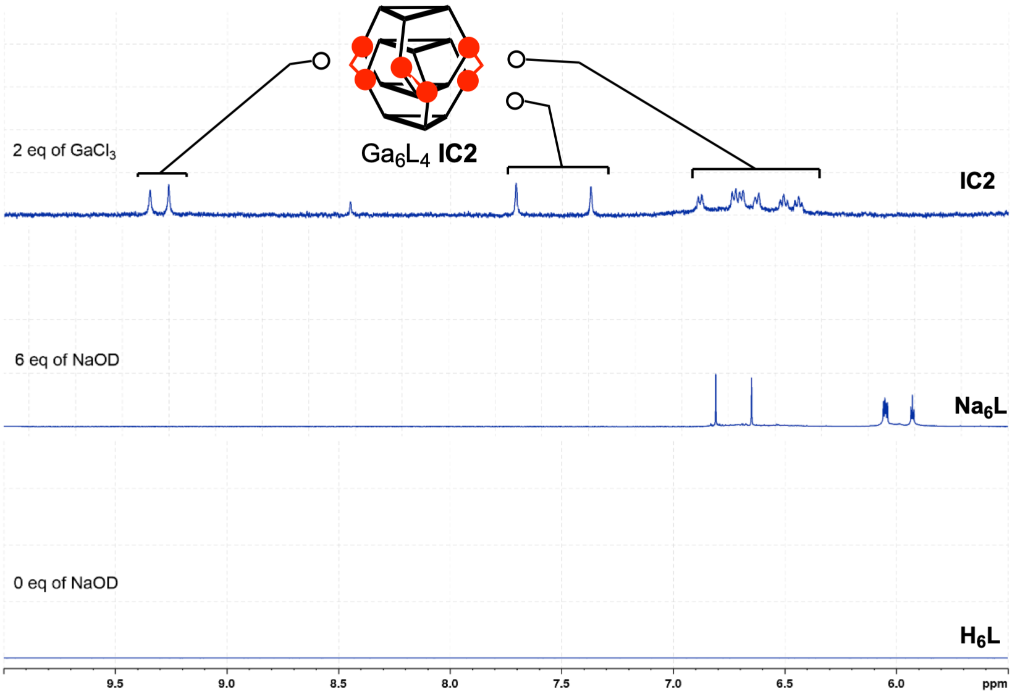

Figure S67. Stacked ^{1}H NMR (500 MHz, D_2O, 298 K, 4 mM) spectra illustrating the self-assembly of **IC2** via titration with $GaCl_3$ (2 equiv) of **L**$^{6-}$. Spectra were recorded 2 minutes after each addition, showcasing the swift temporal dynamics of the assembly process.

S6.5 In-situ NMR monitoring of the deprotonation–reprotonation of H_6L with NaOD–DCl

S6.5.1 In-situ NMR monitoring of the deprotonation of $\mathbf{H_6L}$ with NaOD

General procedure: $\mathbf{H_6L}$ (4.6 mg, 7.62 μmol) was suspended in D_2O (0.5 mL) in a 5 mm NMR tube. To achieve complete deprotonation of $\mathbf{H_6L}$, 6 equiv of NaOD (360 μL from a 0.1267 M stock solution in D_2O) were added, and the mixture was immediately shaken to ensure homogeneity. The reaction mixture was then subjected to ^{1}H, ^{13}C{^{1}H} and DOSY NMR analyses to monitor the deprotonation process, as illustrated in **Figure S68** (^{1}H NMR), **Figure S69** (^{13}C{^{1}H} NMR) and **Figure S70** (DOSY NMR). These *in-situ* NMR experiments reveal the transformation of $\mathbf{H_6L}$ to $\mathbf{Na_6L}$ following the addition of 6 equivalents of NaOD. The disappearance of broad OH resonances associated with $\mathbf{H_6L}$, as shown in **Figure S8**, confirms the deprotonation to $\mathbf{Na_6L}$. Furthermore, the DOSY spectrum provides critical insights into the diffusion coefficients of post-deprotonation species, shedding light on the size and aggregation state of the $\mathbf{L^{6-}}$ molecules in the presence of Na^+ cations. Notably, the diffusion coefficient decreases from 2.60×10^{-10} m^2/s for $\mathbf{H_6L}$ in DMF-d_8 ($\eta = 0.796 \times 10^{-3}$ pa·s at 298 K),[3] to 2.08×10^{-10} m^2/s for $\mathbf{Na_6L}$ in D_2O ($\eta = 1.25 \times 10^{-3}$ pa·s at 298 K).[3] This change underscores the deprotonation effect from $\mathbf{H_6L}$ to $\mathbf{Na_6L}$ and the critical interaction dynamics between $\mathbf{L^{6-}}$ molecules and Na^+ cations, which are pivotal for understanding the mechanisms underlying the supramolecular assembly process.

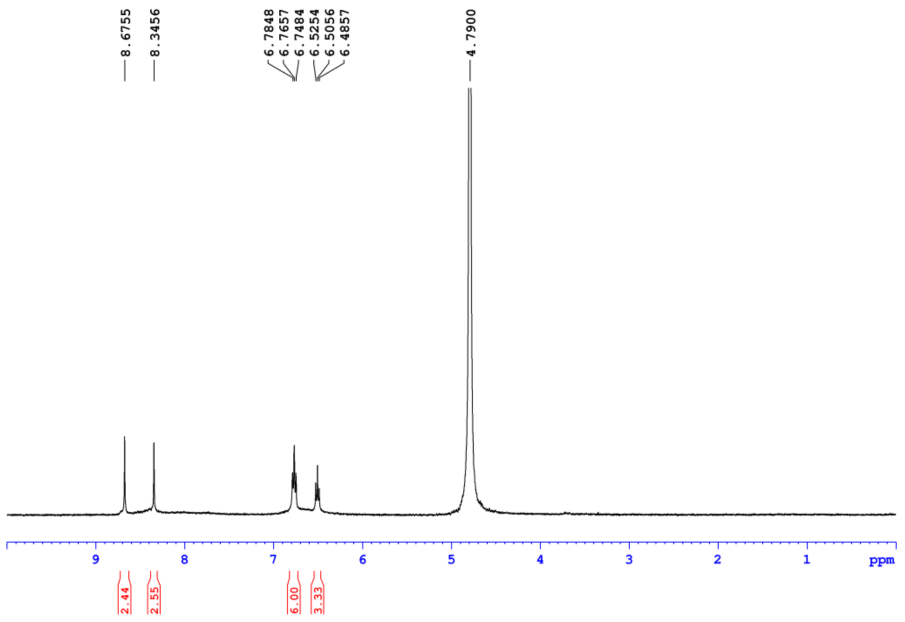

Figure S68. ^{1}H NMR (400 MHz, D_2O, 298 K) spectrum for the *in-situ* deprotonation of $\mathbf{H_6L}$ with NaOD to give $\mathbf{Na_6L}$.

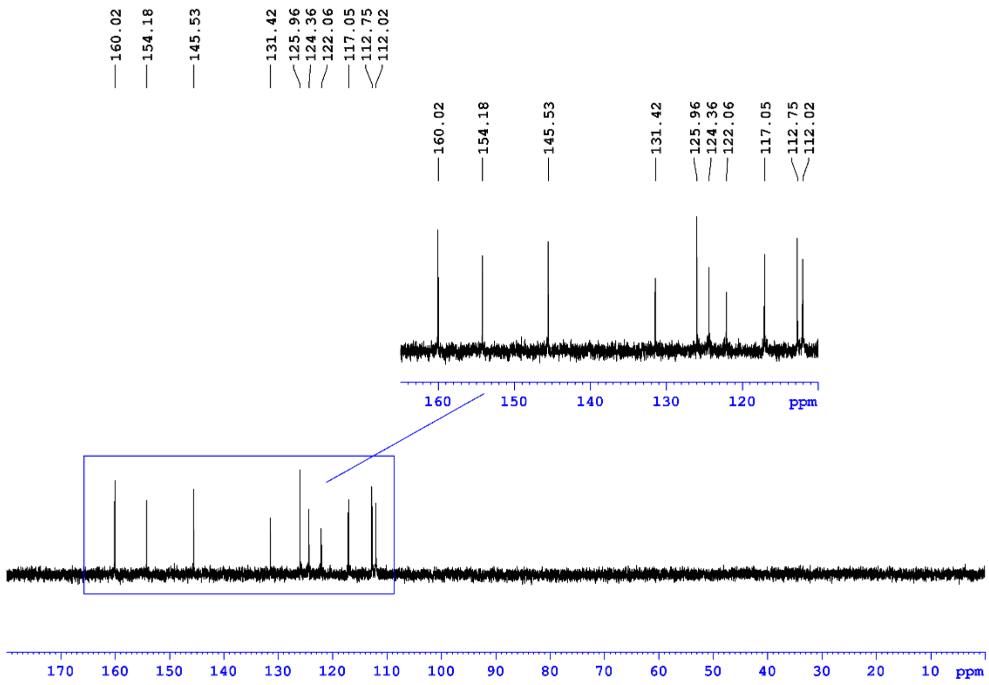

Figure S69. $^{13}C\{^{1}H\}$ NMR (100.6 MHz, D_2O, 298 K) spectrum for the *in-situ* deprotonation of $\mathbf{H_6L}$ with NaOD to give $\mathbf{Na_6L}$.

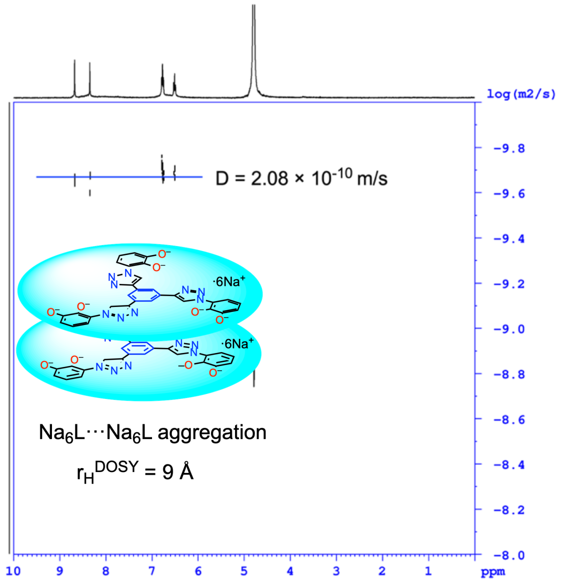

Figure S70. ^{1}H DOSY NMR (400 MHz) following the *in-situ* deprotonation $\mathbf{H_6L}$ with NaOD to give $\mathbf{Na_6L}$.

Table S7. D (m^2s^{-1}) and r_H^{DOSY} for species found following the *in-situ* deprotonation of **H_6L** with NaOD to give **Na_6L** in D_2O at 298 K.

Species	D (m^2s^{-1})	r_H^{DOSY} (Å)
**Na_6L**	2.08×10^{-10}	9

Comments on the ^{1}H DOSY NMR spectrum following the *in-situ* deprotonation of H_6L with NaOD to give Na_6L in D_2O solution: Only one diffusing species is observed in D_2O solution. The observed diffusion coefficient agrees with a hydrodynamic radius (r_H^{DOSY}) of 9 Å suggesting similar aggregation process as seen for **H_6L** and **Me_6L** (aforementioned). In addition, the decrease in the diffusion coefficient from 2.60×10^{-10} m^2/s for **H_6L**, measured in DMF-d_8 ($\eta = 0.796 \times 10^{-3}$ pa·s at 298 K),[3] to 2.08×10^{-10} m^2/s for **Na_6L** in D_2O ($\eta = 1.25 \times 10^{-3}$ pa·s at 298 K),[3] also implies that a slightly larger-sized supramolecular aggregate **Na_6L** has been formed.

S6.5.2 In-situ NMR monitoring of the reprotonation of L^{6-} with DCl

General procedure: Na_6L was prepared by suspending **H_6L** (2.5 mg, 4.14 μmol) in D_2O (0.5 mL) within a 5 mm NMR tube. To initiate deprotonation, sequential addition of aliquots of NaOD (33 μL each, 4.14 μmol, from a 0.1267 M stock solution in D_2O) were added until a total of 10 equiv (330 μL in total, 41.10 μmol), followed by immediate mixing, to ensure complete deprotonation. This transformation was monitored by NMR by measuring a 1H NMR spectrum after each addition (see **Figure S71**). followed by immediate mixing. Subsequent re-protonation was achieved by sequential additions of aliquots of DCl (33 μL each, 4.14 μmol, from a 0.1267 M stock solution in D_2O) to a total of 10 equiv (330 μL, 41.40 μmol), followed by immediate mixing, to ensure complete reprotonation. The deprotonation and reprotonation processes with DCl and NaOD were monitored using *in-situ* ^{1}H and DOSY NMR experiments, capturing the dynamic transition of **Na_6L** back to **H_6L**, as depicted in **Figure S68** and **Figure S71**.

This sequential treatment with NaOD and DCl serves to elucidate the reversible nature of the **H_6L** ↔ **Na_6L** transformation under aqueous conditions. The NMR spectra document the molecular changes occurring during each step: the initial deprotonation by NaOD, leading to the formation of **Na_6L**, followed by the re-protonation by DCl, signifying the recovery of the original **H_6L** species. The ^{1}H NMR spectra in **Figure S68** and **Figure S71**) highlight the chemical shift changes correlating with these transformations, whereas the DOSY NMR spectra (**Figure S70**) provide insight into the molecular size and aggregation changes between the protonated and deprotonated states. This reversible process is fundamental to understanding the dynamic equilibrium of the system and the potential for controlled assembly-disassembly process of **IC1** and **IC2**.

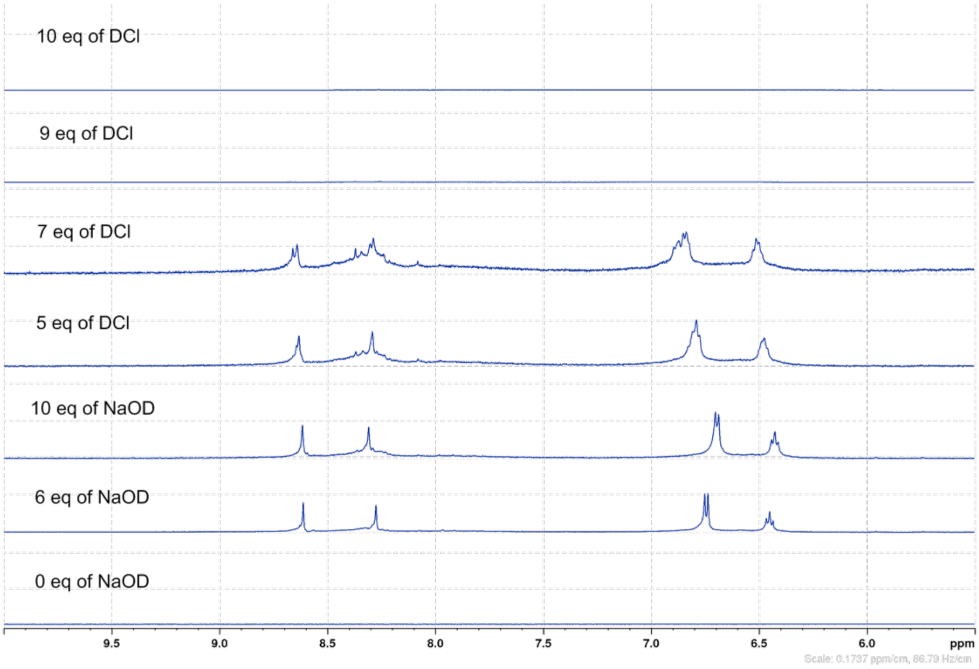

Figure S71. Stacked ^{1}H NMR (500 MHz, D_2O, 298 K) spectra illustrating the deprotonation and reprotonation process $\mathbf{H_6L} \leftrightarrow \mathbf{Na_6L}$ via sequential titration with NaOD (0–10 equiv) and DCl (0–10 equiv). Spectra were recorded 2 minutes after each addition.

S7 Calorimetric studies of the disassembly–reassembly of IC1 and IC2

S7.1 ITC general procedures

Isothermal Titration Calorimetry (ITC) experiments were performed on Nano ITC (TA Instruments) calorimeter equipped with a cylindrical inert sample cell of standard volume (1 mL). Prior to analysis, solutions underwent thorough degassing by stirring under vacuum using a dedicated degassing station.

The sample cell was charged with the corresponding solution of the compound under study, specifically assembled or disassembles aggregates for both **IC1** or **IC2**, while a 250 µL autopipette was loaded with a standardized solution of DCl or NaOD solution. An extensive range of titrations was conducted for both **IC1** and **IC2**, comprising a sequence of 25 injections varying from 0 to 4 equivalents of DCl, followed by NaOD. This approach aimed to delineate the heat changes correlating with the reversible self-assembly process, akin to what was observed in the NMR titrations (see Sections 5.1 and 5.2). The calorimetric data for the full range heat titrations revealed multiple heat changes indicative of various pre-equilibrium states within the assembly and disassembly processes for the supramolecular coordination systems, encompassing DCl, NaOD, and Na^+/K^+ ion pairing exchanges, depicting a complex multi-process supramolecular landscape. Subsequent targeted titrations focused on specific heat changes of interest, identified from the broad analysis, related to the effective steps of disassembly and assembly for **IC1** and **IC2**. These steps corresponded closely with observations from ^{1}H NMR

spectroscopic titrations (see Sections 5.1 and 5.2), where distinct changes were noted for **IC1** between 0-1.5 equivalents of DCl for disassembly, and 2-4 equivalents of NaOD for assembly. In the case of **IC2**, spectral shifts occurred between 2-4 equivalents of DCl for disassembly and 0-1.5 equivalents of NaOD for assembly. We found that these ranges were particularly clean in the ITC experiments with sensible data fittings.

All calorimetric measurements were performed at 1 mM in D_2O using DCl/NaOD for consistency with the NMR titrations. Conducting ITC experiments at 1 mM allowed clear, reproducible thermodynamic analysis and ensured homogeneous solution conditions throughout the entire titration procedure, thereby enhancing data reliability and interpretability. Blank injections of DCl into D_2O or NaOD solutions were subtracted, so the reported ΔH and ΔS values represent only cage processes. The small solvent-isotope shift (~0.4 pK units and < 2 kJ mol^{-1} in neutralization enthalpy) is negligible compared with the 16 kJ mol^{-1} **IC1** vs **IC2** stability gap, identical switching behavior was verified in H_2O.

The intervals between ITC injections were meticulously evaluated between 1000 and 2400 seconds between injections, with a 1200-second interval determined as optimal. Furthermore, the volume for each injection was fine-tuned for precision in the calorimetric readings for each specific heat titration. Detailed parameters for each calorimetric experiment are delineated in the subsequent sections below. Data analysis was conducted using the NanoAnalyze software package. Water-into-water validation runs performed to correct any heat changes arising from injections and to establish an accurate baseline, with ambient room temperature being strictly controlled at 298 K throughout the experiments, thereby minimizing errors in the calorimetric measurements. Data fitting using either independent or multi-site fitting models provided comparable results.

High-precision weighing of compounds was achieved using a Mettler Toledo microbalance (accurate to 1 µg), and solutions were prepared with Eppendorf micropipettes. Standardized commercially available solutions of deuterium chloride DCl (7.6 N in D_2O) and sodium deuteroxide NaOD (40% weight in D_2O), purchased from Eurisotop, were used to ensure consistency and comparison with the NMR titrations.

S7.2 ITC study of the disassembly–reassembly of IC1

Disassembly of IC1: The disassembly was investigated using a 1 mM solution of **IC1** (5.93 mg, 2.0 µmol in 2 mL of degassed D_2O). For this titration, a 22.8 mM DCl solution was prepared from a 7.6 M commercial standardized solution. For the titration, 950 µL of the 1 mM **IC1** solution was loaded in the sample cell. The titration sequence involved 25 incremental additions of 2.86 µL of the 22.8 mM DCl solution, spaced by an optimized interval of 1200 seconds for equilibration. The volume of DCl added ranged from 0 to 1.7 equivalents in the subsequent 25 injections. The latter was identified as the effective heat window for observing assembly, as seen in the NMR titrations. To ensure the accuracy

and consistency of results, this titration was also performed multiple times with consistent and reproducible results.

Reassembly of IC1: For the assembly phase, a 1 mM solution of **IC1** in D_2O (2 mL) was initially disassembled using 3 equiv of DCl (39 µL, 6 µmol from a 0.152 M solution). The resulting disassembled solution (950 µL) was then titrated with a 22.8 mM NaOD solution in D_2O. The titration sequence involved two additions of 20 µL, followed by 30 incremental additions of 4 µL each of the 22.8 mM NaOD solution, spaced by an optimized interval of 1200 seconds for equilibration. The volume of NaOD added ranged from 0 to 1 equiv in the first two injections, extending from 1 to 4.2 equivalents in the subsequent 30 injections. The latter was identified as the effective heat window for observing disassembly, as seen in the NMR titrations. To ensure the accuracy and consistency of results, this titration was also performed multiple times with consistent and reproducible results.

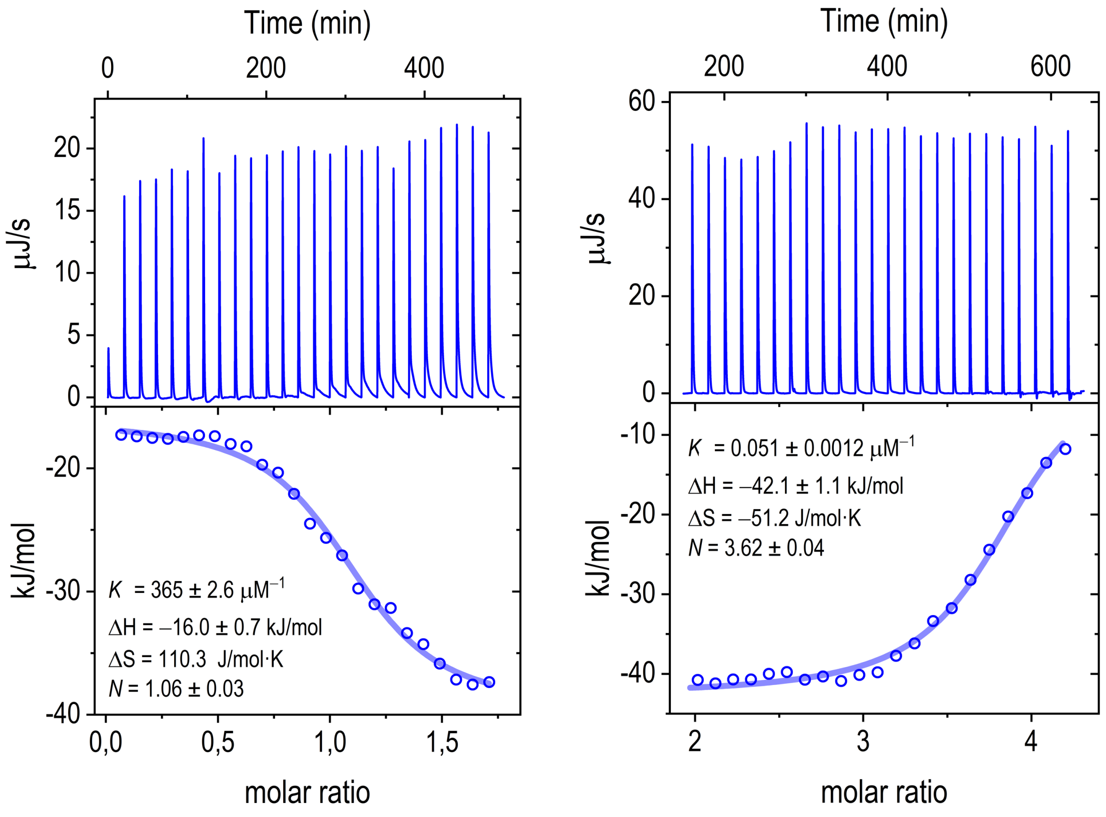

Figure S72. ITC titration plot and fitted curves (25ºC, D_2O) obtained through titrations of **IC1** with sequential additions of DCl (left: disassembly occurred between 0–1.7 equiv of DCl) and NaOD (right: reassembly occurred between 2–4 equiv of NaOD).

S7.3 ITC study of the dissasembly–reassembly of IC2

Disassembly of IC2: The disassembly was investigated using a 1 mM solution of **IC2** (6.43 mg, 2.0 µmol in 2 mL of degassed D_2O). For this titration, a 22.8 mM DCl solution was prepared from a 7.6 M commercial standardized solution. For the titration, 950 µL of the 1 mM **IC2** solution was loaded in the

sample cell. The titration sequence involved three additions of 20 µL, followed by 30 incremental additions of 2.86 µL each of the 22.8 mM DCl solution, spaced by an optimized interval of 1200 seconds for equilibration. The volume of DCl added ranged from 0 to 1.5 equivalents in the first three injections, extending from 1.5 to 3.8 equivalents in the subsequent 30 injections. The latter was identified as the effective heat window for observing disassembly, as seen in the NMR titrations. To ensure the accuracy and consistency of results, this titration was also performed multiple times with consistent and reproducible results.

Reassembly of IC2: For the assembly phase, a 1 mM solution of **IC2** in D_2O (2 mL) was initially disassembled using 3 equiv of DCl (39 µL, 6 µmol from a 0.152 M solution). The resulting disassembled solution (950 µL) was then titrated with a 11.4 mM NaOD solution in D_2O. The titration sequence comprised 41 additions of 2.86 µL of the NaOD solution, with an optimized 1200-second interval between injections to allow reaching an equilibrium situation between additions. The cumulative addition of NaOD throughout these injections spanning from 0 to 1.5 equivalents, was identified as the effective heat window for observing assembly, as seen in the NMR titrations. To ensure the accuracy and consistency of results, this titration was also performed multiple times with consistent and reproducible results.

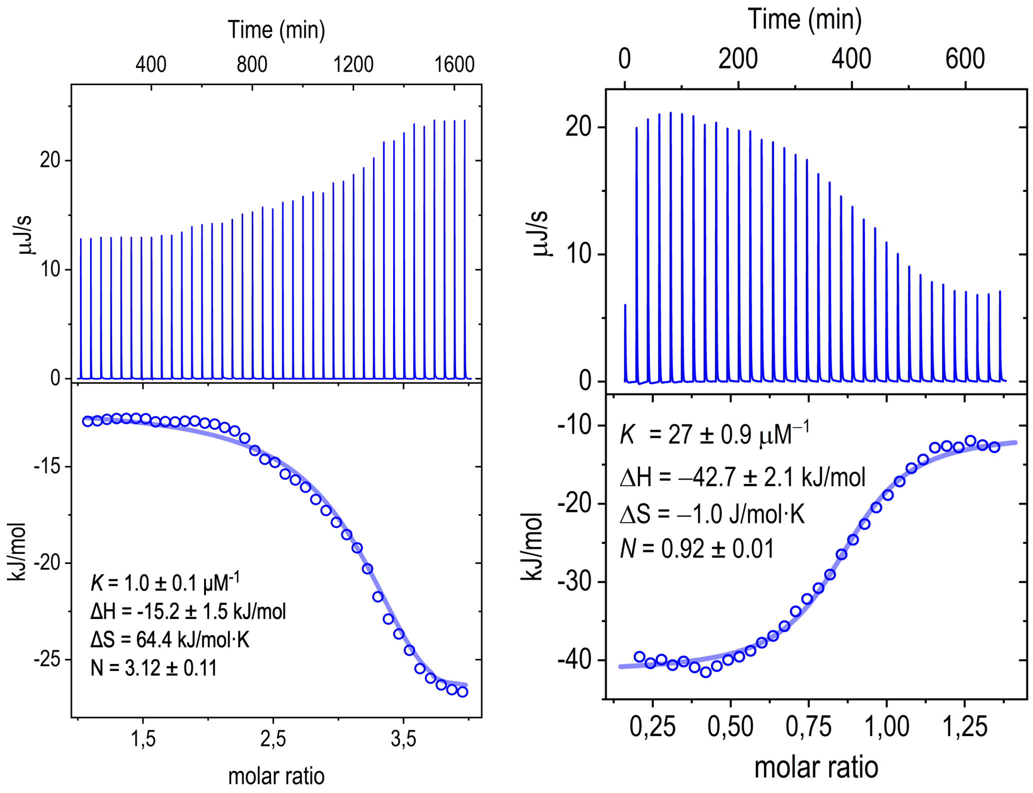

Figure S73. ITC titration plot and fitted curves (25ºC, D_2O) obtained through titrations of **IC2** with sequential additions of DCl (left: disassembly occurred between 1–4 equiv of DCl) and NaOD (right: reassembly occurred between 0–1.5 equiv of NaOD).

S8 Lyophilization studies of the disassembly of IC1 and IC2

S8.1 Lyophilization and characterization of disassembled species for IC1

Lyophilization techniques were used for isolating and characterizing species involved in self-assembly processes as it allows for the removal of solvent while minimizing the disruption of the sample.

General procedure: A solution of **IC1** (38.3 mg, 13.0 μmol) was prepared in D_2O (2 mL). The initial ^1H NMR spectrum of an aliquot, serving as a baseline, was recorded at time zero (t = 0). To induce disassembly, an addition of an aliquot of 4 equiv of DCl (340 μL, 52 μmol) from a stock solution (152 mM in D_2O) were added, and a ^1H NMR spectrum of an aliquot was measured showing complete disassembly of the system. At this point, following disassembly, the aqueous suspension was rapidly frozen using liquid nitrogen and subsequently placed under high vacuum (< 10^{-2} mbar) overnight for lyophilization at room temperature. This process successfully removed all D_2O, yielding a brown solid. The lyophilized product was directly used for characterization. Due to the complexity of the reaction mixtures obtained upon disassembly of **IC1**, reliable NMR characterization in solution gave a complex reaction mixture, therefore, characterization was performed exclusively by HRMS spectrometry and IR spectroscopy. _Note:_ The strongly acidified lyophilized disassembly mixture shows dominant signals for partially protonated species $[Al_2(H_xL)_2]^{n-}$ and a minor peak at m/z = 425.0586 (Figure S66), assignable to $Al_3L_2^{3-}$ **C1**. We attribute this trace to in-source fragmentation of residual amounts of **IC1**, **C1** is not observed in solution under neutral or basic conditions during assembly/disassembly experiments.

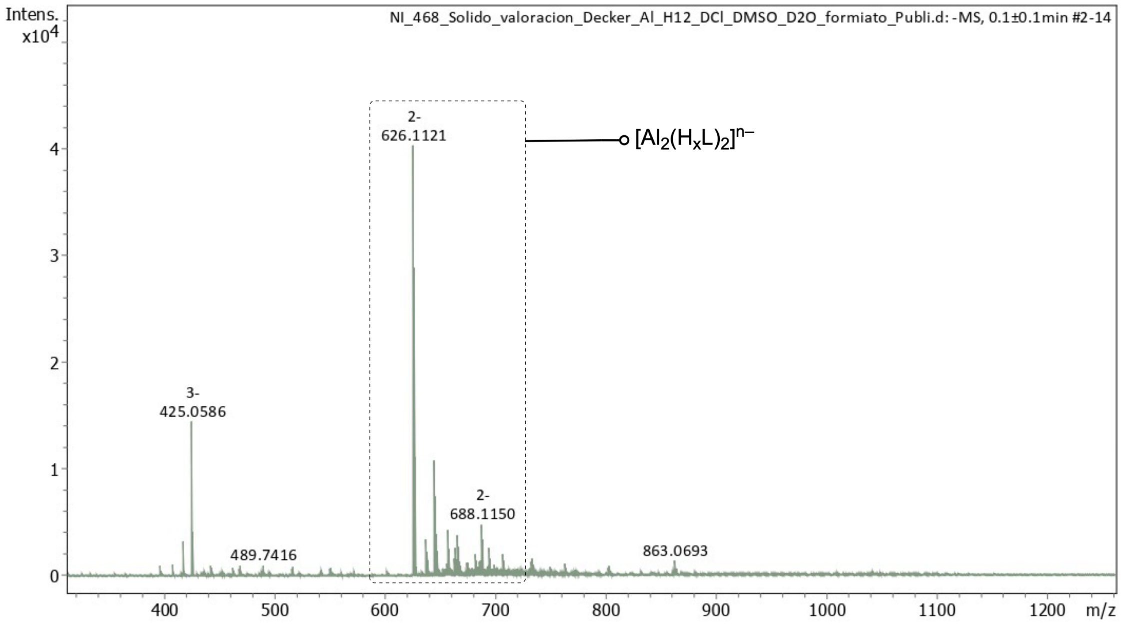

Figure S74. Full view for the high-resolution mass spectra (ESI) corresponding to lyophilized disassembled species from **IC1**. Selected sections highlighted with a dashed box are those areas of interest that were fully studied in detail.

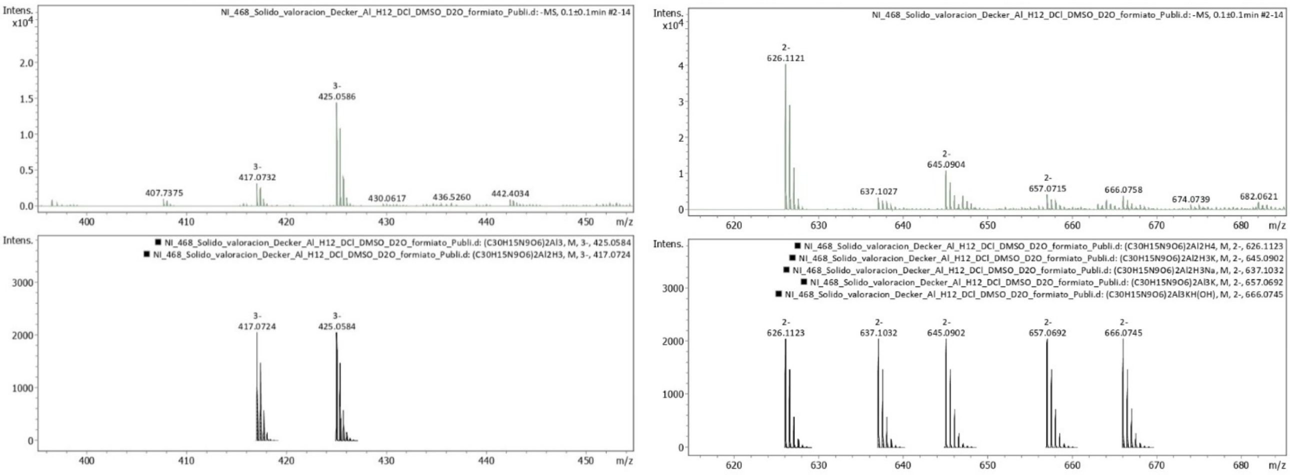

Figure S75. Section of the high-resolution mass spectra (ESI) corresponding to lyophilized disassembled species from **IC1** corresponding to [**$Al_2(HL)_2H$**]$^{3-}$ and **[Al_3L_2]**$^{3-}$ (left) and **[$Al_2(H_2L)_2$]**$^{2-}$, **[$Al_2(HL)_2H$ + Na]**$^{2-}$, **[$Al_2(HL)_2H$ + K]**$^{2-}$, **Al_3L_2 + K]**$^{2-}$ and **[$Al_3L_2H(OH)$ + K]**$^{2-}$ (right). Top: experimental, bottom: calculated.

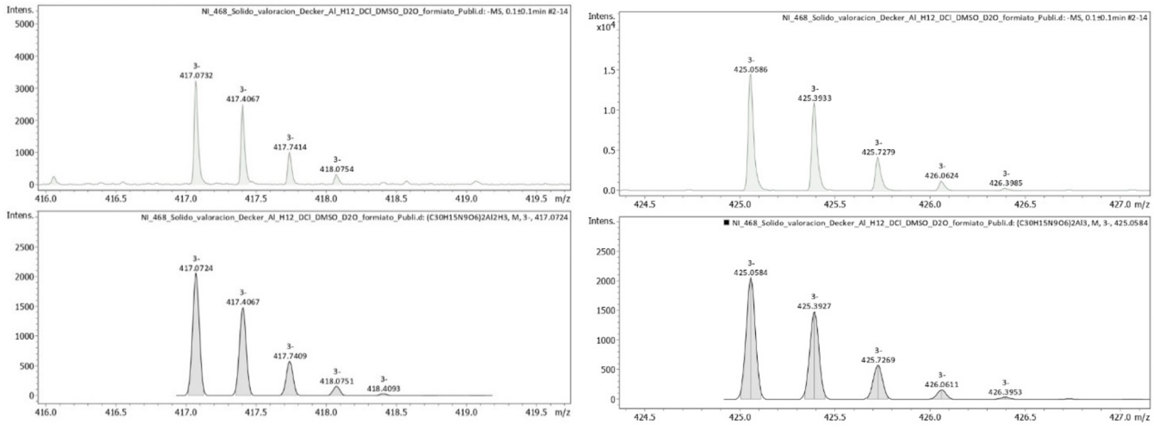

Figure S76. Section of the high-resolution mass spectra (ESI) corresponding to lyophilized disassembled species from **IC1** corresponding to [**$Al_2(HL)_2H$]**$^{3-}$ (left) and **[Al_3L_2]**$^{3-}$ (right). Top: experimental, bottom: calculated.

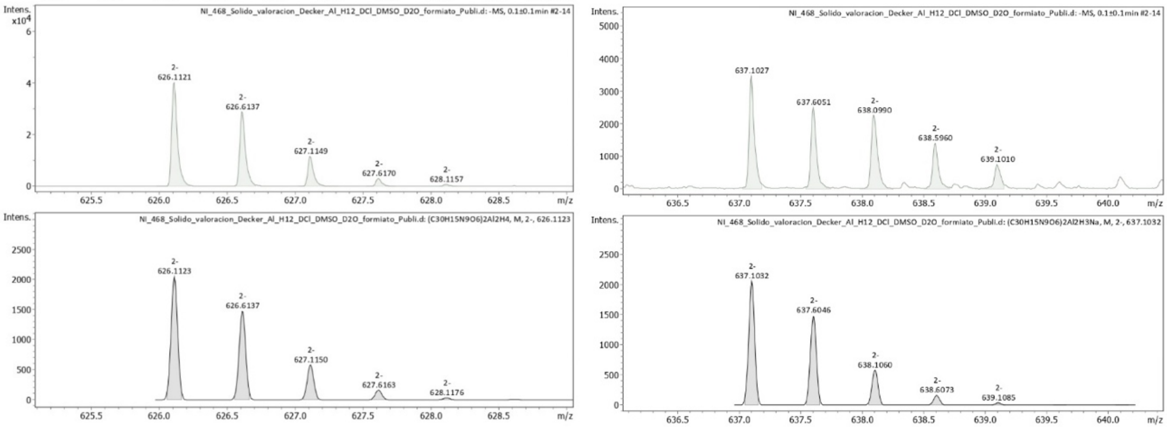

Figure S77. Section of the high-resolution mass spectra (ESI) corresponding to lyophilized disassembled species from **IC1** corresponding to **$[Al_2(H_2L)_2]^{2-}$** (left) and **$[Al_2(HL)_2H + Na]^{2-}$** (right). Top: experimental, bottom: calculated.

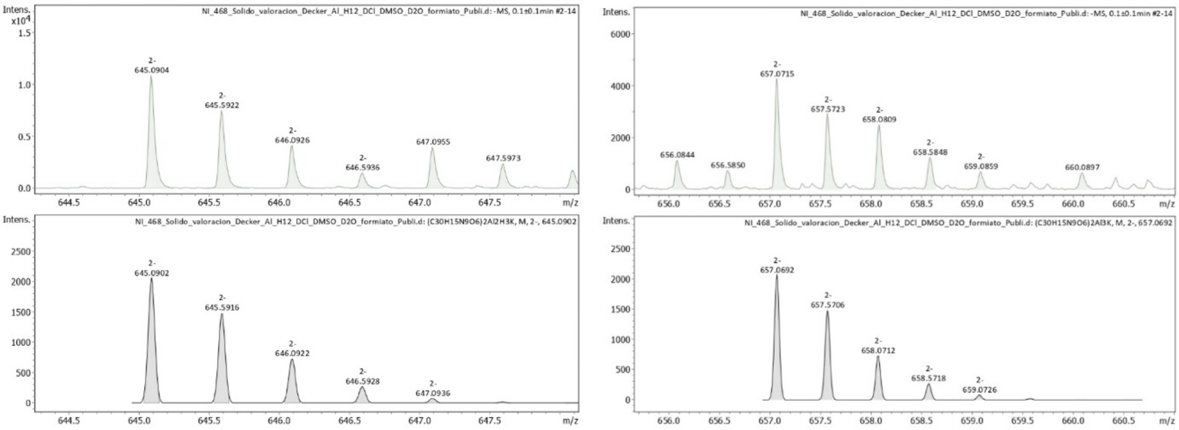

Figure S78. Section of the high-resolution mass spectra (ESI) corresponding to lyophilized disassembled species from **IC1** corresponding to **$[Al_2(HL)_2H + K]^{2-}$** (left) and **$[Al_3L_2 + K]^{2-}$** (right). Top: experimental, bottom: calculated.

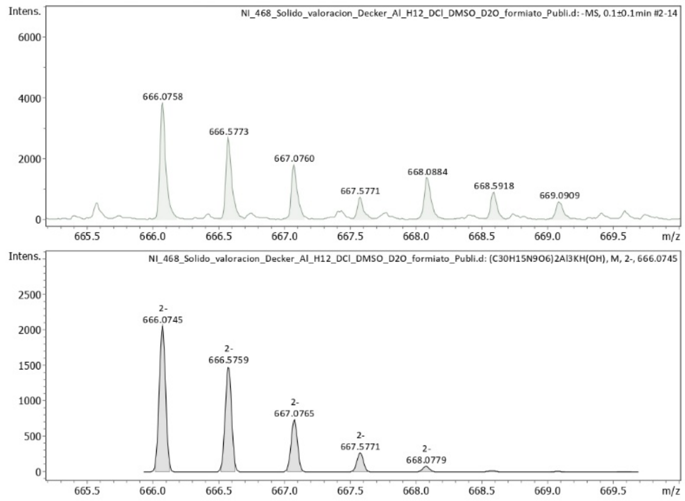

Figure S79. Section of the high-resolution mass spectra (ESI) corresponding to lyophilized disassembled species from **IC1** corresponding to **$[Al_3L_2H(OH) + K]^{2-}$.** Top: experimental, bottom: calculated.

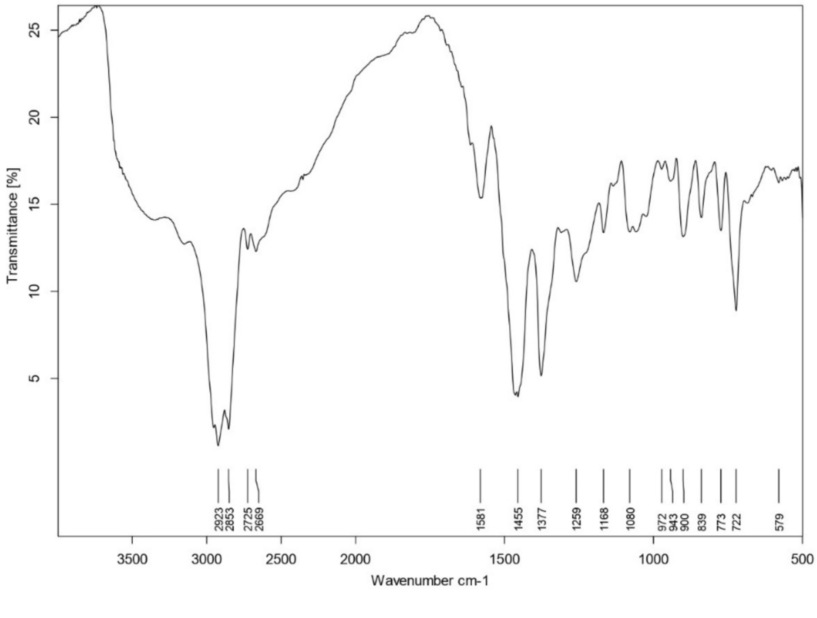

Figure S80. IR (KBr/Nujol, cm^{-1}) spectrum of lyophilized disassembled species from **IC1**.

S8.2 Lyophilization and characterization of disassembled species for IC2

General procedure: A solution of **IC2** (33.5 mg, 11.7 μmol) was prepared in D_2O (2 mL). The initial ^{1}H NMR spectrum of an aliquot, serving as a baseline, was recorded at time zero (t = 0). To induce disassembly, addition of an aliquot of 4 equiv of DCl (368 μL, 46.8 μmol) from a stock solution (126.7 mM in D_2O) were added, and a ^{1}H NMR spectrum of an aliquot was measured showing complete disassembly of the system. At this point, following disassembly, the suspension was rapidly frozen using liquid nitrogen and subsequently placed under high vacuum (< 10^{-2} mbar) overnight for lyophilization at room temperature. This process successfully removed all D_2O, yielding a brown solid. The lyophilized product was directly used for characterization by NMR, HRMS and IR.

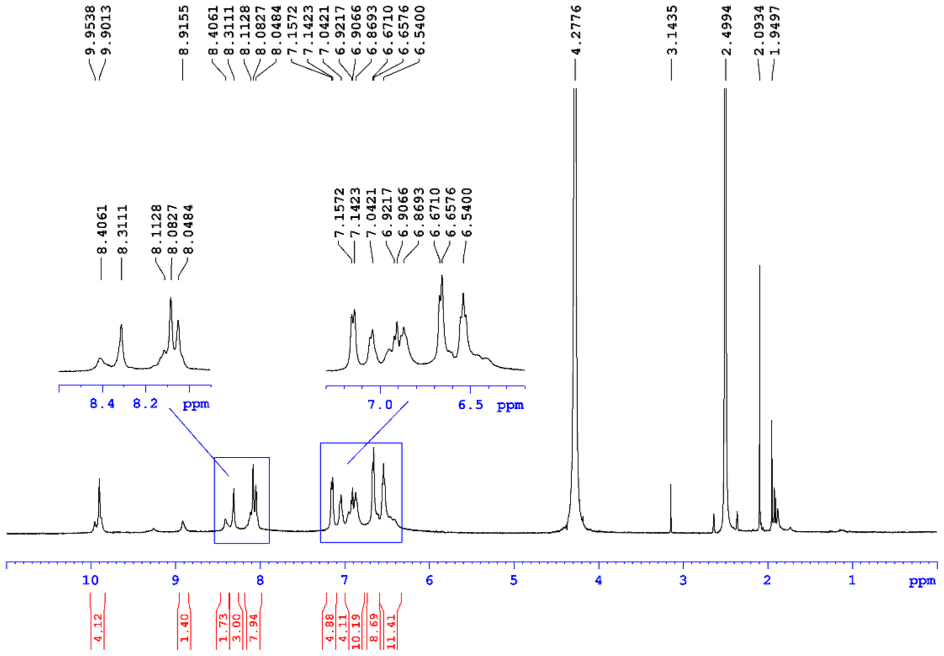

Figure S81. ^{1}H NMR (500 MHz, DMSO-d_6:D_2O 3:2, 298 K) spectrum of lyophilized disassembled species from **IC2**.

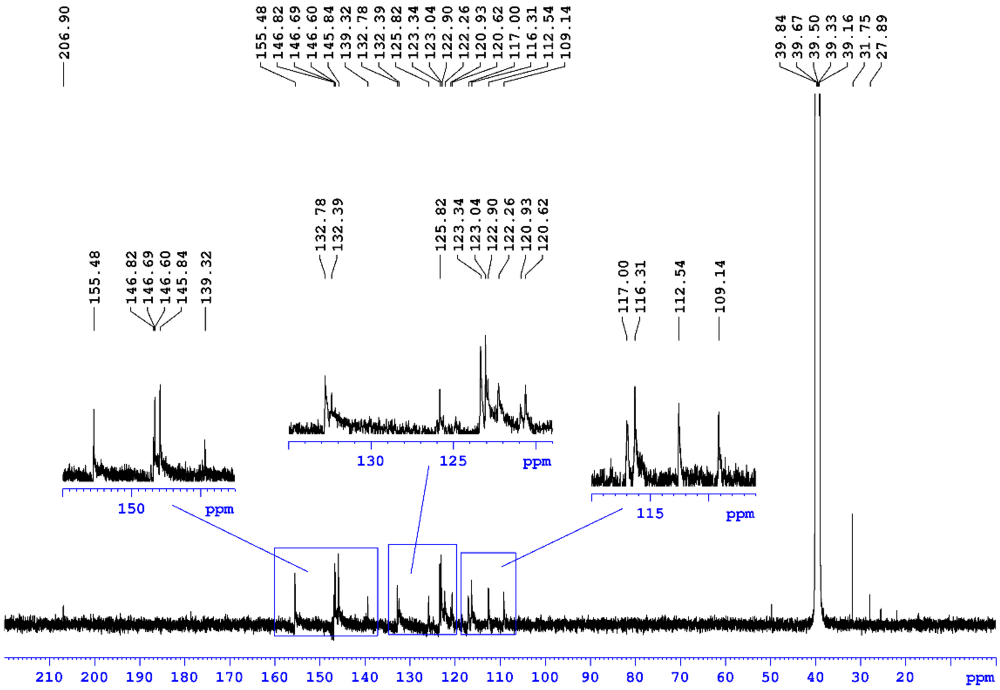

Figure S82. $^{13}C\{^{1}H\}$ NMR (125.6 MHz, DMSO-d_6:D_2O 3:2, 298 K) spectrum of lyophilized disassembled species from **IC2**.

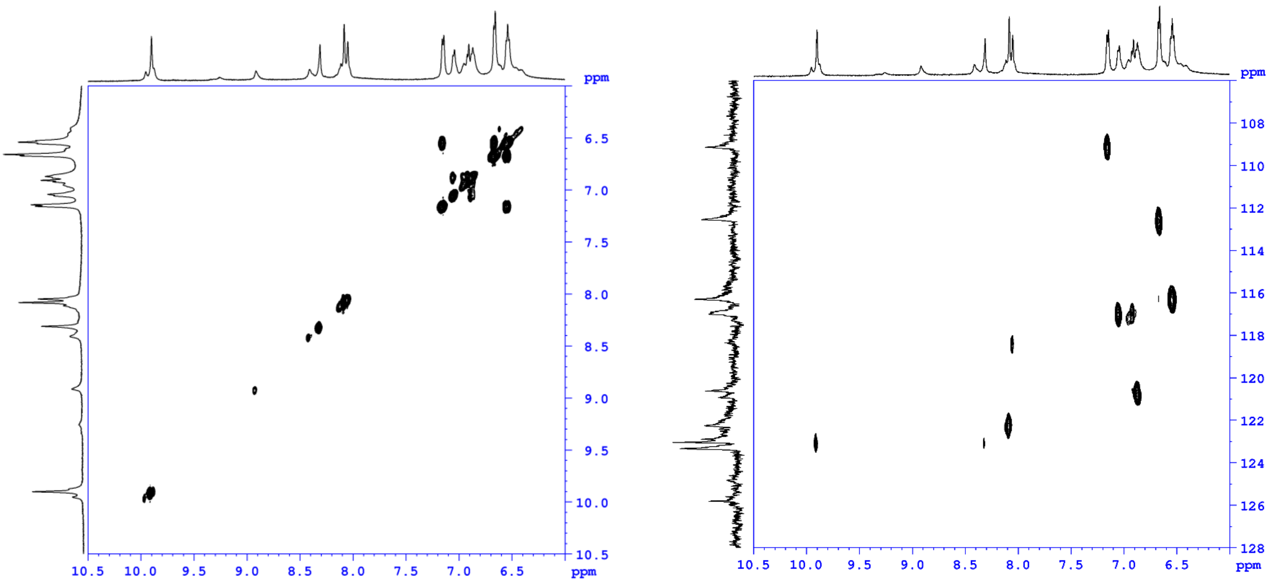

Figure S83. $^{1}H,^{1}H$-COSY (left) and $^{1}H,^{13}C$-HSQC (right) NMR (500 MHz, DMSO-d_6:D_2O 3:2, 298 K) spectrum of lyophilized disassembled species from **IC2**.

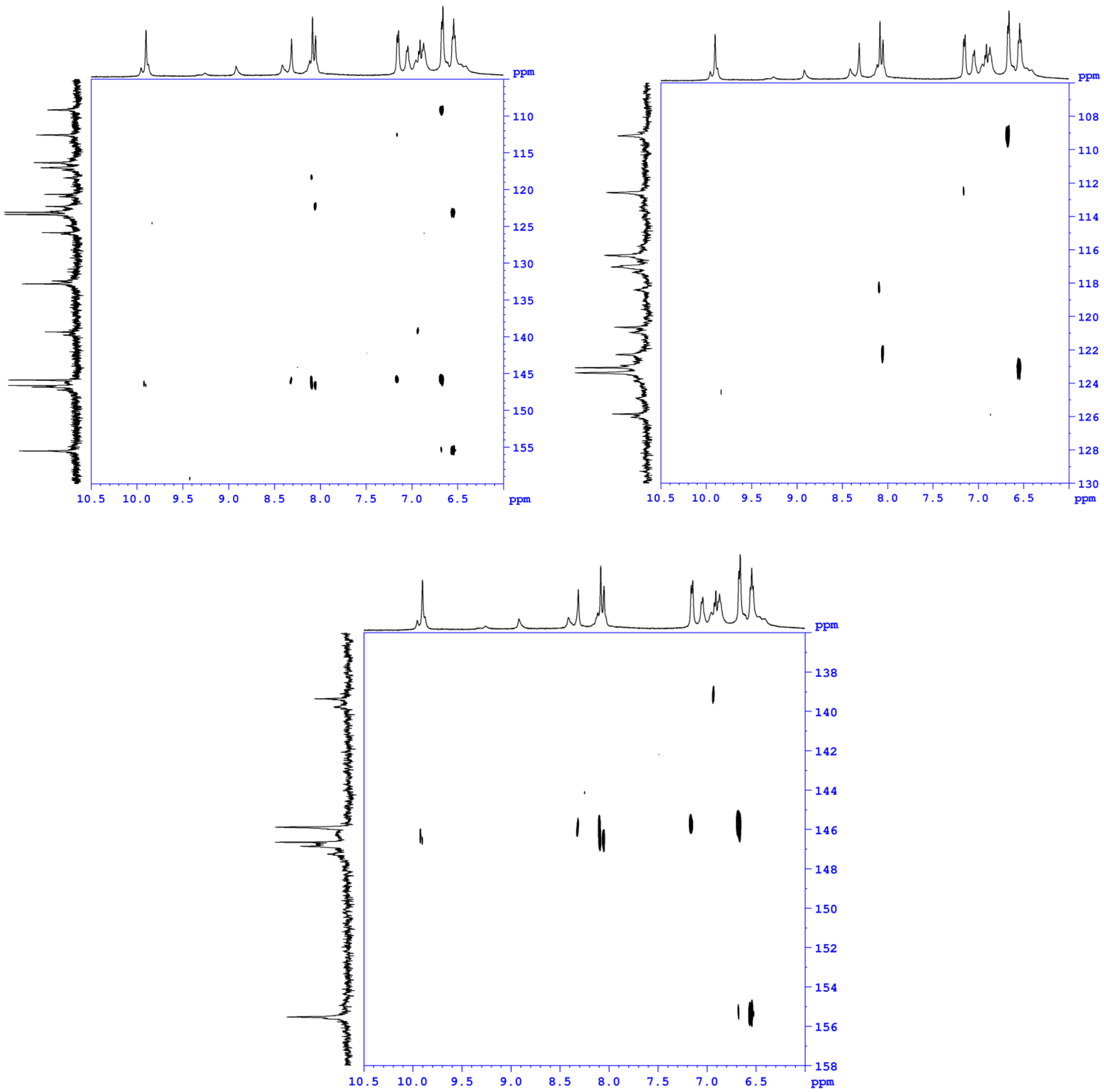

Figure S84. Selected sections of the ^{1}H,^{13}C-HMBC NMR (500 MHz, DMSO-d_6:D_2O 3:2, 298 K) spectrum of lyophilized disassembled species from **IC2**.

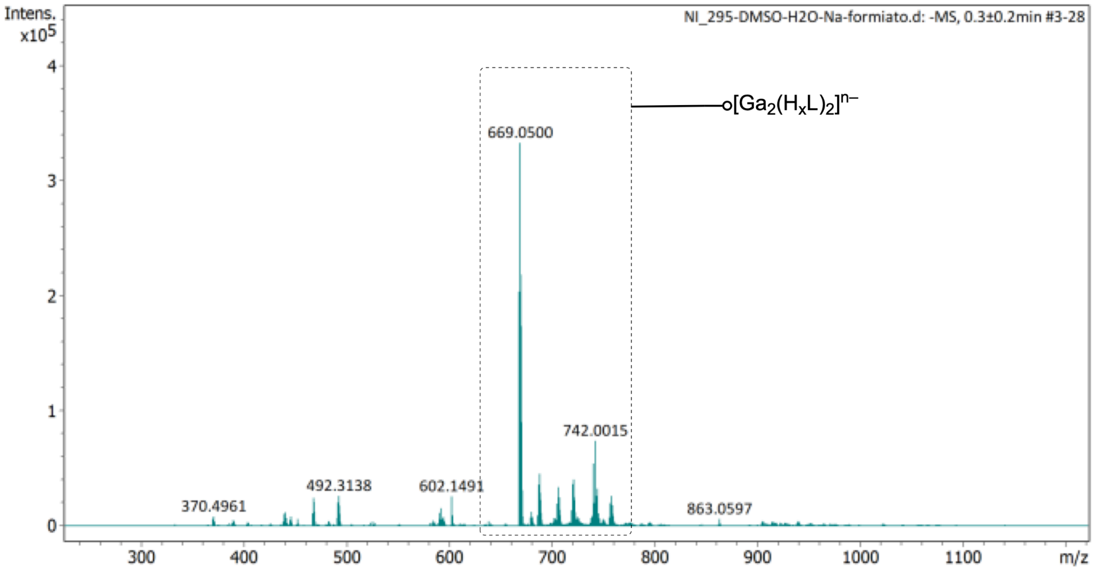

Figure S85. Full view for the high-resolution mass spectra (ESI) corresponding to lyophilized disassembled species from **IC2**. Selected sections highlighted with a dashed box are those areas of interest that were fully studied in detail. Section of the high-resolution mass spectra (ESI) corresponding to lyophilized disassembled species from **IC2** corresponding to **$[Ga_2(H_2L)_2]^{2-}$, $[Ga_2(HL)_2H + K]^{2-}$** and **$[Ga_2(HL)_2 +2 K]^{2-}$**. Top: experimental, bottom: calculated.

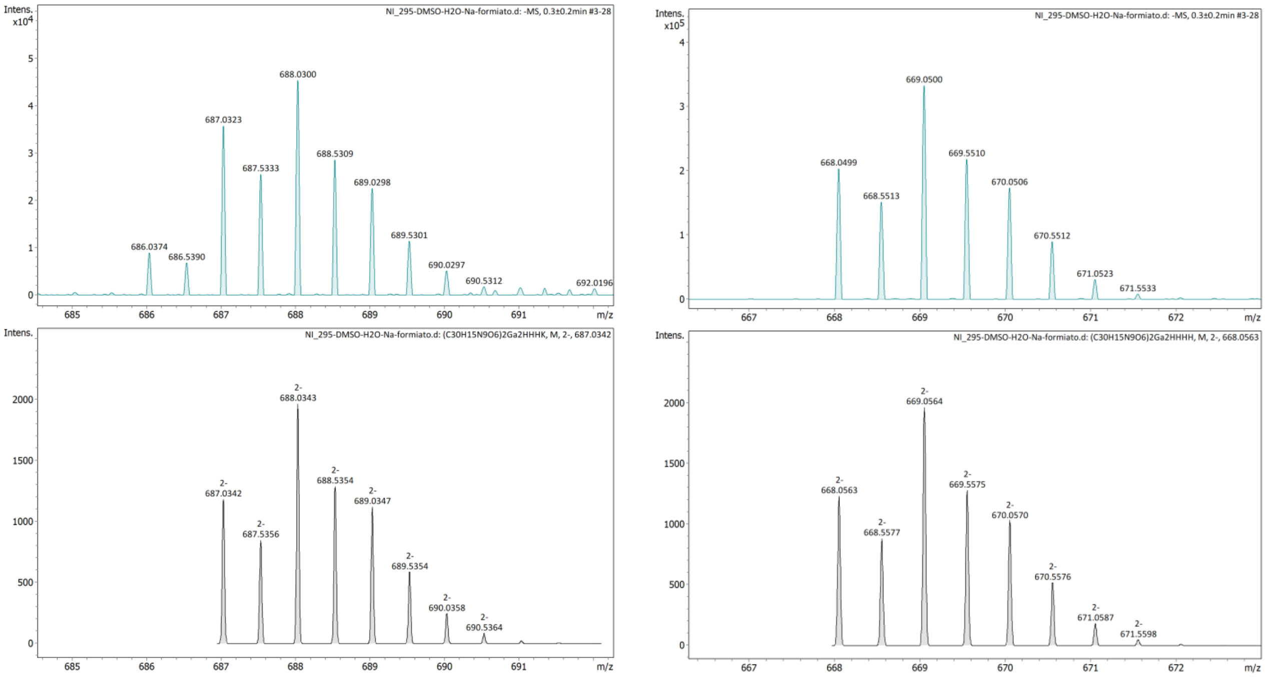

Figure S86. Section of the high-resolution mass spectra (ESI) corresponding to lyophilized disassembled species from **IC2** corresponding to **$[Ga_2(HL)_2H + K]^{2-}$** (left) and **$[Ga_2(H_2L)_2]^{2-}$** (right). Top: experimental, bottom: calculated.

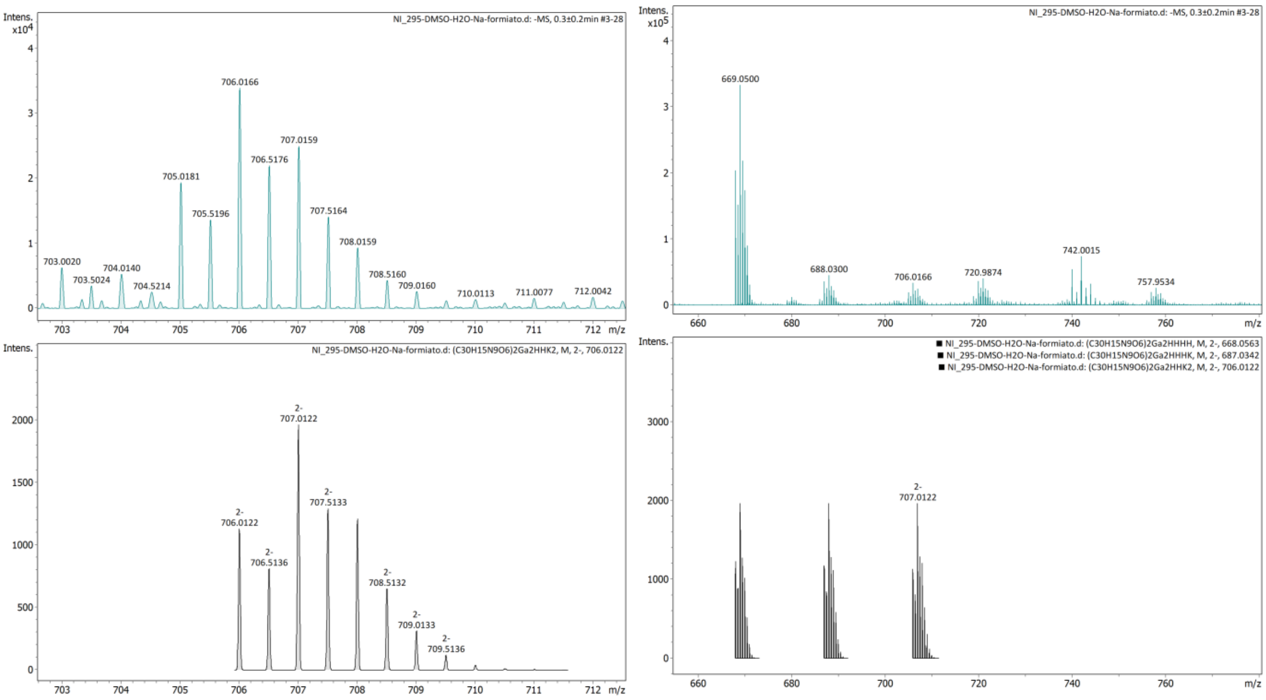

Figure S87. Section of the high-resolution mass spectra (ESI) corresponding to lyophilized disassembled species from **IC2** corresponding to **[$Ga_2(HL)_2$ + 2K]$^{2-}$** (left) and **[$Ga_2(H_2L)_2$]$^{2-}$, [$Ga_2(HL)_2$H + K]$^{2-}$** and **[$Ga_2(HL)_2$ + 2K]$^{2-}$** (right). Top: experimental, bottom: calculated.

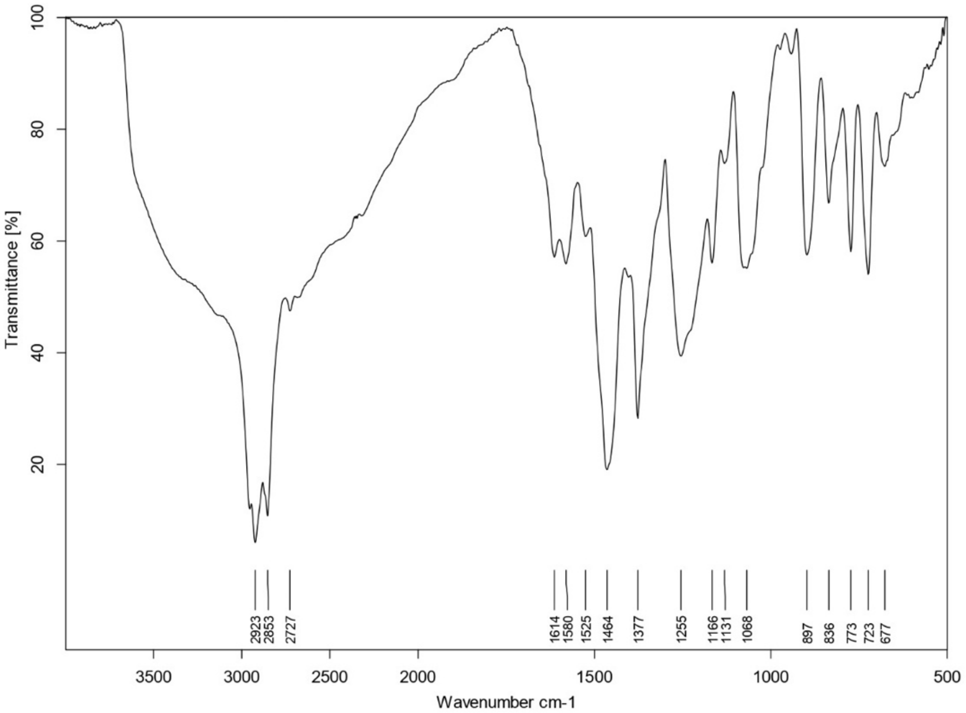

Figure S88. IR (KBr/Nujol, cm^{-1}) spectrum of lyophilized disassembled species from **IC2**.

S9 X-ray crystallography

S9.1 Single-crystal X-ray structural determination details

Single-crystal X-ray diffraction data was collected under a stream of nitrogen gas using an Oxford Cryostream 1000 unit at the given temperature with a Bruker D8 Quest Eco diffractometer (micro-focus sealed X-ray tube, Mo-Kα, λ = 0.71073 Å) equipped with a Photon II 7. Raw frame data were reduced using APEX3. The structures were solved using SHELXT[5] and refined using full-matrix least squares refinement on all F^2 data using the SHELXL.[6] Unless otherwise stated, all non-hydrogen atoms were refined anisotropically and hydrogen atoms were geometrically placed and allowed to ride on their parent atoms. Disorder was treated by introducing a split site model and restraining geometries and displacement parameters. For **IC2**, a good model for outer-sphere solvent molecules was achieved by modelling several molecules of methanol, acetone and water molecules from crystallization. However, because some severe disorder affecting unmodelled solvent molecules and the presence of a solvent accessible voids in the structure, to obtain a good refinement and model, PLATON/SQUEEZE was applied to treat this diffuse contribution, identifying a volume of 515 Å^3 per unit cell (5.4% of the cell volume) containing 41 electrons (i.e. 2.05 H_2O per formula unit, Z = 2). The empirical formula and all derived quantities were updated accordingly. Distances and angles were calculated using the full covariance matrix. Selected crystallographic data is summarized in the text. Full details are given in the supplementary deposited CIF files (CCDC 2467878 for **Me$_6$L** and 2467879 for **IC2**). These data can be obtained free of charge from the Cambridge Crystallographic Data Centre via http://www.ccdc.cam.ac.uk/data_request/cif.

S9.2 Selected crystallographic and refinement data

Table S8. Crystal data and structure refinement for **Me_6L** and **IC2**.

**Compound**	**Me_6L**	**IC2**
**CCDC number**	**2467878**	**2467879**
Formula	$C_{36}H_{33}N_9O_6$	$C_{120}H_{63}Ga_6K_9N_{36}O_{29}$, $(C_3H_6O)_{7.75}$, $(CH_4O)_{1.5}$, $(H_2O)_{19.8}$
Fw	687.71	4098.16
Crystal system	Monoclinic	Triclinic
Space group	P21/n	P–1
a/Å	10.3821(2)	21.3348(4)
b/Å	27.7393(5)	21.5438(4)
c/Å	11.5669(2)	21.7803(4)
α/°	90	78.6820(10)
β/°	102.0500(10)	78.7120(10)
γ/°	90	81.9500(10)
Volume/$Å^3$	3257.78(10)	9572.5(3)
Z	4	2
λ(Mo-K_{α})/Å	0.71073	0.71073
Temperature/*K*	100.0	150.0
ρ(calcd, mg m^{-3})	1.402	1.422
μ(mm^{-1})	0.099	1.116
F(000)	1440	4194
Crystal size (mm^3)	0.26 × 0.24 × 0.22	0.18 × 0.14 × 0.14
Crystal color	light brown	brown
Crystal shape	block	block
Refls. collect.	198296	631004
Index ranges	-14<=h<=14, -39<=k<=39, -16<=l<=16	-28<=h<=28, -28<=k<=28, -28<=l<=28
Independent refls.	9960	45718
Θ range for data collection	2.323 to 30.528°	0.968 to 27.916
Completeness to 25.242º	99.8%	99.8%
R_{int}	0.0422	0.0323
Goodness of fit	1.127	1.040
$R[F^2>2\sigma]$, *F*	0.0593	0.0489
R_w (all data), F^2	0.1533	0.1536
Restrains/parameters	0/466	801 / 2545
Residual electron density (e $Å^{-3}$)	0.567/–0.233	1.452 /–1.284

Additional details for IC2 (2467879): Crystals of **IC2** were measured at 100, 150, and 260 K and consistently showed partial loss of crystallinity upon removal from the mother liquors, leading to diffuse

residual electron density in the outer-sphere region. Extensive modeling of disordered outer-sphere K^+ ions and solvent molecules (H_2O, methanol and acetone) was carried out with complementary occupancies, mild ADP restraints (SIMU/ISOR/DELU as needed), and inclusion of water H atoms in idealized positions (terminal H_2O as rotating riders, bridging H_2O as restrained non-rotating riders). Several highly disordered water sites in the solvent belt (e.g., O23, O24, O38A, O40, O41) were retained as oxygen-only, as no reliable H-atom peaks were visible and inclusion of idealized H atoms destabilized refinement. Minor disorder at K3/K5 remained but was stable, with reasonable ADPs and only small residuals confined to the highly disordered solvent region. A small solvent-accessible void persisted, and PLATON/SQUEEZE[7] was applied to treat this diffuse outer-sphere solvent contribution, identifying a volume of 515 $Å^3$ per unit cell (5.4% of the cell volume) containing 41 electrons (2.05 H_2O per formula unit, Z = 2). The empirical formula and all derived quantities were updated accordingly. Application of SQUEEZE improved refinement statistics (R1 [$I > 2\sigma(I)$] from 0.0517 to 0.0489; wR2 [all data] from 0.1664 to 0.1536). The anionic $[Ga_6L_4(OH)_3]^{9-}$ cage is fully ordered in **IC2** and unaffected by the solvent mask. All details of the masking procedure are transparently documented in the deposited CIF file CCDC 2467879.

S9.3 Supplementary X-ray figures and metrics

S9.3.1 Crystal structure of $\mathbf{M_6L}$

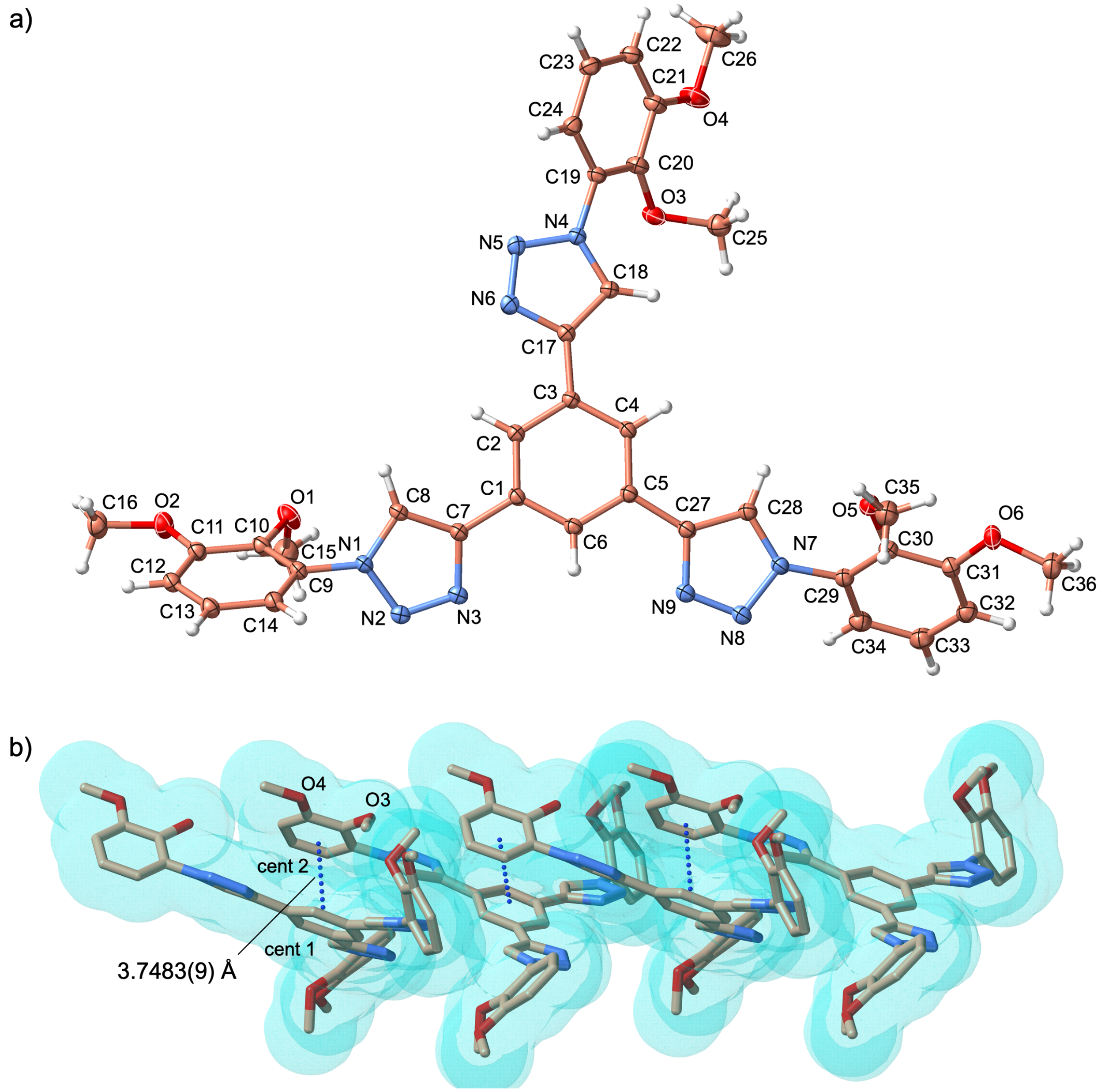

Figure S89. Crystal structure of the ligand $\mathbf{Me_6}$. a) Ellipsoid plot at 50% with atom labelling. B) Stick view with a solvent-accessible surface (probe 1.20 Å) highlighting the packing diagram through $\pi\cdots\pi$ contacts (cent1$\cdots$cent2 = 3.7483(9)), giving rise to $\pi\cdots\pi$ chains. Hydrogen atoms omitted in b) for clarity. Selected bond lenghts (Å) and angles (°): O(1)-C(10) 1.3716(17), O(1)-C(15) 1.441(2), O(2)-C(11) 1.3598(17), O(2)-C(16) 1.4390(19), O(3)-C(20) 1.3681(17), O(3)-C(25) 1.4335(19), O(4)-C(21) 1.3599(19), O(4)-C(26) 1.428(2), O(5)-C(30) 1.3698(17), O(5)-C(35) 1.4400(19), O(6)-C(31) 1.3645(17), O(6)-C(36) 1.4368(18), N(1)-N(2) 1.3501(16), N(1)-C(8) 1.3515(17), N(1)-C(9)

1.4286(16), N(2)-N(3) 1.3106(17), N(3)-C(7) 1.3668(17), N(4)-N(5) 1.3510(16), N(4)-C(18) 1.3570(17), N(4)-C(19) 1.4246(17), N(5)-N(6) 1.3048(18), N(6)-C(17) 1.3707(18), N(7)-N(8) 1.3615(16), N(7)-C(28) 1.3542(17), N(7)-C(29) 1.4292(17), N(8)-N(9) 1.3054(17), N(9)-C(27) 1.3725(18), C(7)-C(8) 1.3766(18), C(17)-C(18) 1.3722(18), C(27)-C(28) 1.3807(18), N(2)-N(1)-C(8) 110.79(11), N(2)-N(1)-C(9) 120.53(11), C(8)-N(1)-C(9) 128.68(12), N(3)-N(2)-N(1) 107.19(11), N(2)-N(3)-C(7) 109.33(12), N(5)-N(4)-C(18) 110.73(11), N(5)-N(4)-C(19) 117.98(11), C(18)-N(4)-C(19) 131.26(11), N(6)-N(5)-N(4) 107.01(11), N(5)-N(6)-C(17) 109.79(12), N(8)-N(7)-C(29) 117.89(11), C(28)-N(7)-N(8) 110.82(11), C(28)-N(7)-C(29) 131.20(12), N(9)-N(8)-N(7) 107.07(11), N(8)-N(9)-C(27) 109.55(11), N(3)-C(7)-C(1) 121.95(12), N(3)-C(7)-C(8) 107.84(11), N(1)-C(8)-C(7) 104.83(12), C(10)-C(9)-N(1) 118.40(12), C(14)-C(9)-N(1) 119.37(12), N(6)-C(17)-C(3) 120.02(12), N(6)-C(17)-C(18) 107.57(12), N(4)-C(18)-C(17) 104.91(12), C(20)-C(19)-N(4) 119.74(12), C(24)-C(19)-N(4) 118.37(12), N(9)-C(27)-C(5) 120.34(12), N(9)-C(27)-C(28) 107.91(12), N(7)-C(28)-C(27) 104.64(12), C(30)-C(29)-N(7) 119.97(12), C(34)-C(29)-N(7) 117.88(12).

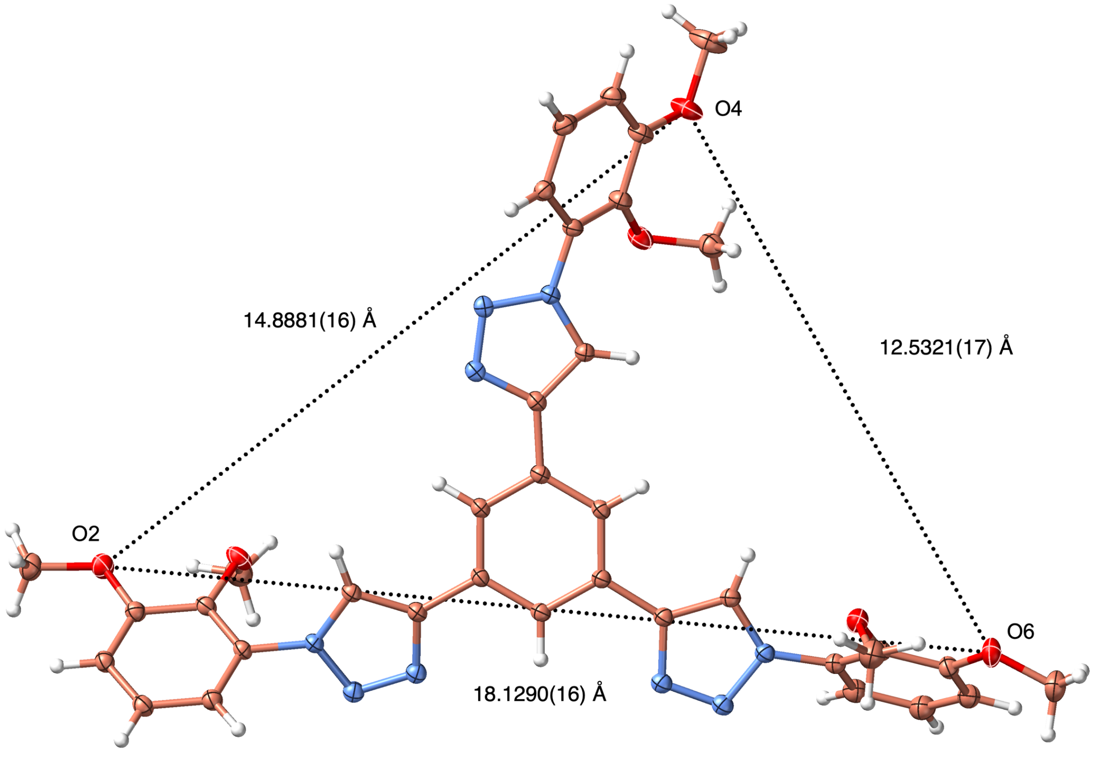

Figure S90. Crystal structure of the ligand **Me$_6$L** showing the interatomic O···O separations between external oxygen atoms from the $C_6H_4(OMe)_2$ groups: O2···O4 14.8881(16) Å, O4···O6 12.5321(17) Å and O2···O6 18.1290(16). Displacement ellipsoids at 50% probability.

S9.3.2 Crystal structure of IC2

a)

b)

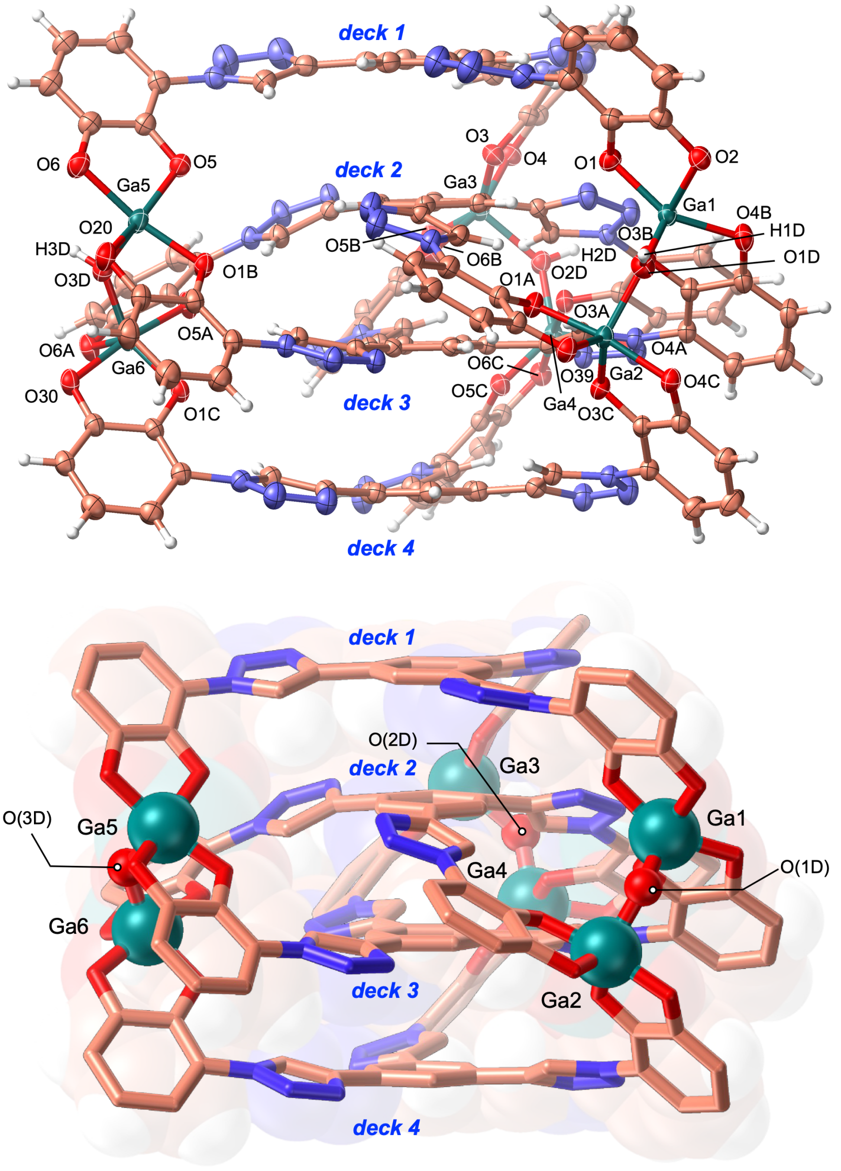

Figure S91. Crystal structure of the triply interlocked anionic cage **IC2** $[Ga_6L_4(OH)_3]^{9-}$. a) Ellipsoid plot (50% probability) highlighting Ga atoms (teal), and μ-OH bridges and catecholate–oxygen atoms (red). b) Ball-and-stick view with Van-der-Walls surface highlighting the quadruple-decker arrangements (decks 1–4) with key μ-OH bridges (O(1D), O(2D), O(3D)) and Ga atoms (Ga1–Ga6) shown as red and teal balls, respectively. Disordered outer-sphere K^+ counterions and olvent molecules (H_2O, methanol and acetone) from crystallisation are omitted for clarity. The core cage is fully ordered and unaffected by the solvent disorder. Selected bond lenghts (Å) and angles (°): Ga(1)-O(1) 1.910(2), Ga(1)-O(1D) 1.890(2), Ga(1)-O(2) 1.933(2), Ga(1)-O(3B) 1.9010(19), Ga(1)-O(4B) 1.924(2), Ga(2)-K(1) 3.6317(7), Ga(2)-O(1A) 1.9343(19), Ga(2)-O(1D) 1.901(2), Ga(2)-O(3C) 1.9010(19), Ga(2)-O(4C) 1.9544(19), Ga(2)-O(39) 1.8956(19), Ga(3)-O(2D) 1.8998(19), Ga(3)-O(3) 1.9087(19), Ga(3)-O(4) 1.9375(19), Ga(3)-O(5B) 1.9028(18), Ga(3)-O(6B) 1.9201(19), Ga(4)-O(2D)

1.9081(19), Ga(4)-O(3A) 1.9360(18), Ga(4)-O(4A) 1.8904(18), Ga(4)-O(5C) 1.8926(18), Ga(4)-O(6C) 1.9479(19), Ga(5)-O(1B) 1.898(2), Ga(5)-O(3D) 1.928(2), Ga(5)-O(5) 1.918(2), Ga(5)-O(6) 1.936(2), Ga(5)-O(20) 1.931(2), Ga(6)-O(1C) 1.8931(19), Ga(6)-O(3D) 1.906(2), Ga(6)-O(5A) 1.9370(19), Ga(6)-O(6A) 1.897(2), Ga(6)-O(30) 1.9481(19), O(1)-Ga(1)-O(2) 85.52(9), O(1)-Ga(1)-O(4B) 144.66(10), O(1D)-Ga(1)-O(1) 110.69(9), O(1D)-Ga(1)-O(2) 98.31(9), O(1D)-Ga(1)-O(3B) 98.41(9), O(1D)-Ga(1)-O(4B) 104.56(10), O(3B)-Ga(1)-O(1) 91.30(8), O(3B)-Ga(1)-O(2) 163.02(9), O(3B)-Ga(1)-O(4B) 86.07(8), O(4B)-Ga(1)-O(2) 86.93(9), O(1A)-Ga(2)-O(4C) 171.45(9), O(1D)-Ga(2)-O(1A) 95.00(9), O(1D)-Ga(2)-O(4C) 93.39(9), O(3C)-Ga(2)-O(1A) 88.03(8), O(3C)-Ga(2)-O(1D) 128.68(9), O(3C)-Ga(2)-O(4C) 85.45(8), O(39)-Ga(2)-O(1A) 86.00(8), O(39)-Ga(2)-O(1D) 107.90(9), O(39)-Ga(2)-O(3C) 123.42(9), O(39)-Ga(2)-O(4C) 92.98(8), O(2D)-Ga(3)-O(3) 110.62(9), O(2D)-Ga(3)-O(4) 97.30(8), O(2D)-Ga(3)-O(5B) 98.98(8), O(2D)-Ga(3)-O(6B) 101.52(9), O(3)-Ga(3)-O(4) 85.31(8), O(3)-Ga(3)-O(6B) 147.70(9), O(5B)-Ga(3)-O(3) 91.27(8), O(5B)-Ga(3)-O(4) 163.53(9), O(5B)-Ga(3)-O(6B) 86.74(8), O(6B)-Ga(3)-O(4) 87.60(8), O(2D)-Ga(4)-O(3A) 94.65(8), O(2D)-Ga(4)-O(6C) 91.71(9), O(3A)-Ga(4)-O(6C) 173.46(8), O(4A)-Ga(4)-O(2D) 105.39(8), O(4A)-Ga(4)-O(3A) 86.12(8), O(4A)-Ga(4)-O(5C) 125.10(8), O(4A)-Ga(4)-O(6C) 93.66(8), O(5C)-Ga(4)-O(2D) 129.51(8), O(5C)-Ga(4)-O(3A) 89.34(8), O(5C)-Ga(4)-O(6C) 85.44(8), O(1B)-Ga(5)-O(3D) 98.33(9), O(1B)-Ga(5)-O(5) 92.71(9), O(1B)-Ga(5)-O(6) 165.38(10), O(1B)-Ga(5)-O(20) 86.89(9), O(3D)-Ga(5)-O(6) 96.15(10), O(3D)-Ga(5)-O(20) 97.30(9), O(5)-Ga(5)-O(3D) 105.86(9), O(5)-Ga(5)-O(6) 85.24(9), O(5)-Ga(5)-O(20) 156.64(10), O(20)-Ga(5)-O(6) 89.29(9), O(1C)-Ga(6)-O(3D) 126.79(9), O(1C)-Ga(6)-O(5A) 89.28(8), O(1C)-Ga(6)-O(6A) 124.73(9), O(1C)-Ga(6)-O(30) 85.77(8), O(3D)-Ga(6)-O(5A) 95.40(9), O(3D)-Ga(6)-O(30) 92.60(9), O(5A)-Ga(6)-O(30) 172.00(9), O(6A)-Ga(6)-O(3D) 108.48(9), O(6A)-Ga(6)-O(5A) 85.70(8).

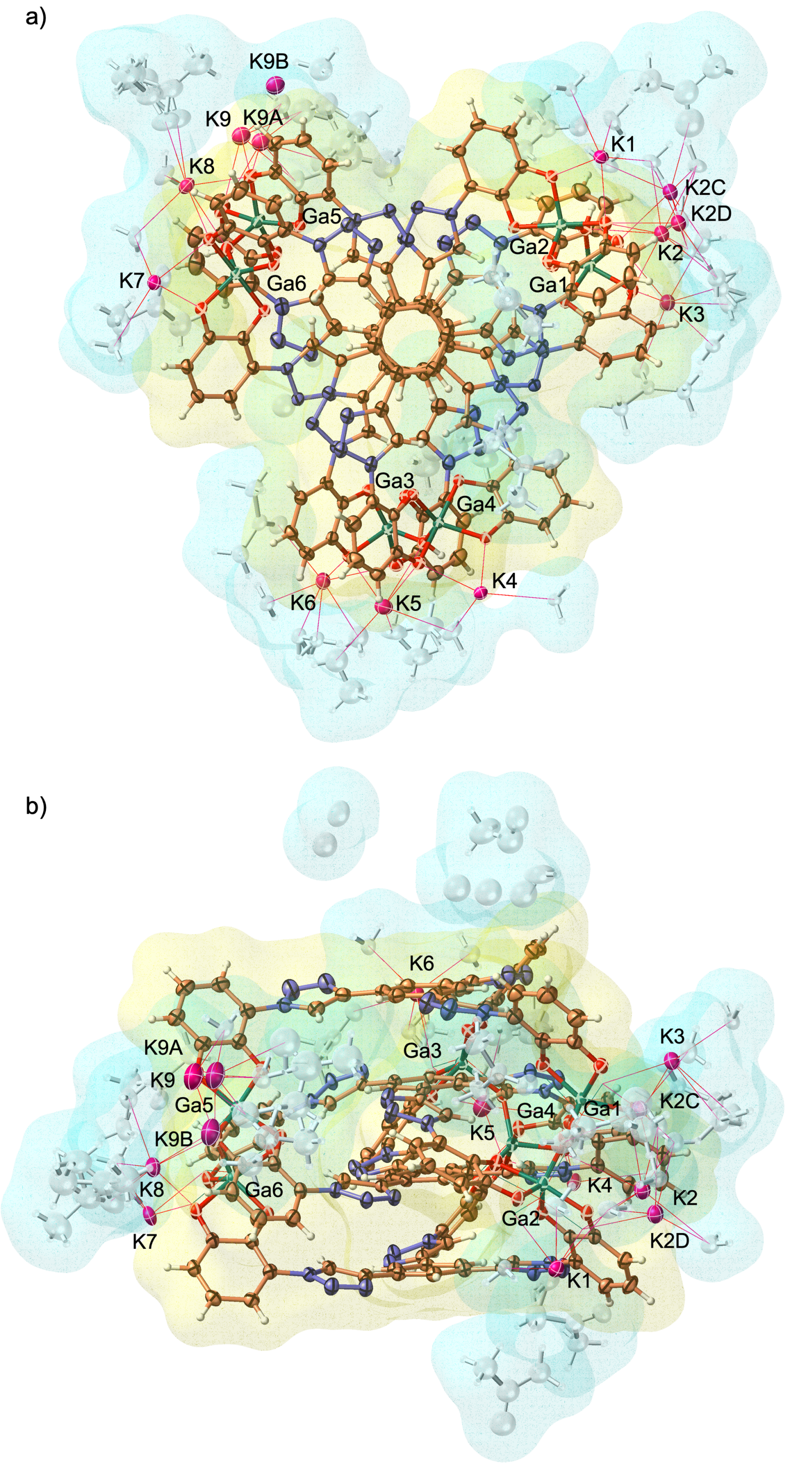

Figure S92. a) Top and b) side views of the outer-sphere disorder in **IC2**. Displacement-ellipsoid plot (50% probability). The ordered anionic cage $[Ga_6L_4(OH)_3]^{9-}$ is highlighted by a solvent-accessible surface (probe 1.2 Å) and the outer-spere region containing disordered K^+ counterions (split positions: K1, K2/K2C/K2D, K3, K4, K5, K6, K7, K8, K9/K9A/K9B) and disordered crystallization solvent (H_2O, MeOH and acetone) that occupies the channels is visualized by a cyan solvent-accessible surface (probe 1.20 Å). Only Ga and K atoms are labelled for clarity; full site labels, refined occupancies, and restraints are given in the CIF file.

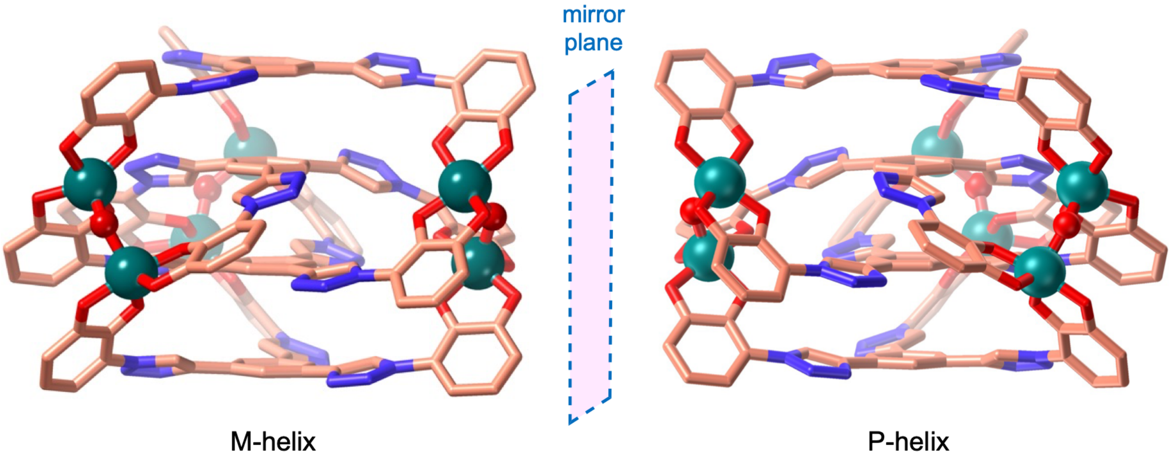

Figure S93. Ball-and-stick representation of the M- (left) and P-helical (right) isomers within the crystalline structure of **IC2** transformed through and inversion of the geometry. H atoms, K^+ ions, and solvent are omitted, C beige, N blue, O red, Ga teal.

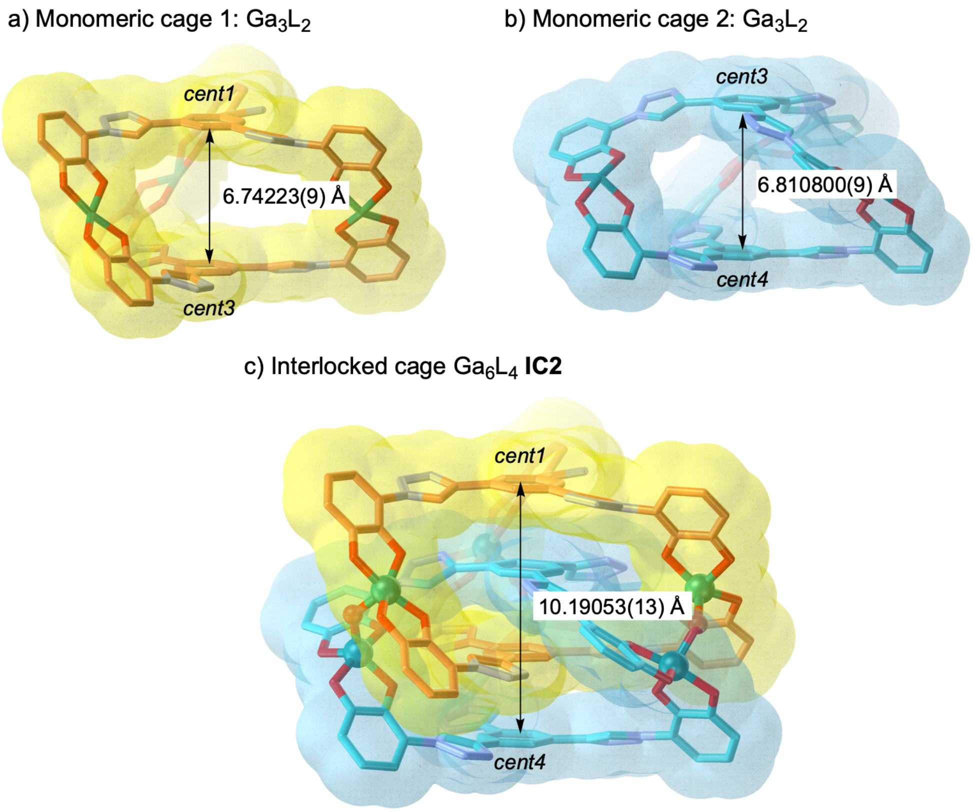

Figure S94. Illustration showing the monomeric cages Ga_3L_2 a) Cage 1 and b) Cage 2 with solvent-accessible surface (probe 1.20 Å) withing the P-helical crystalline structure of c) the interlocked cage **IC2**.

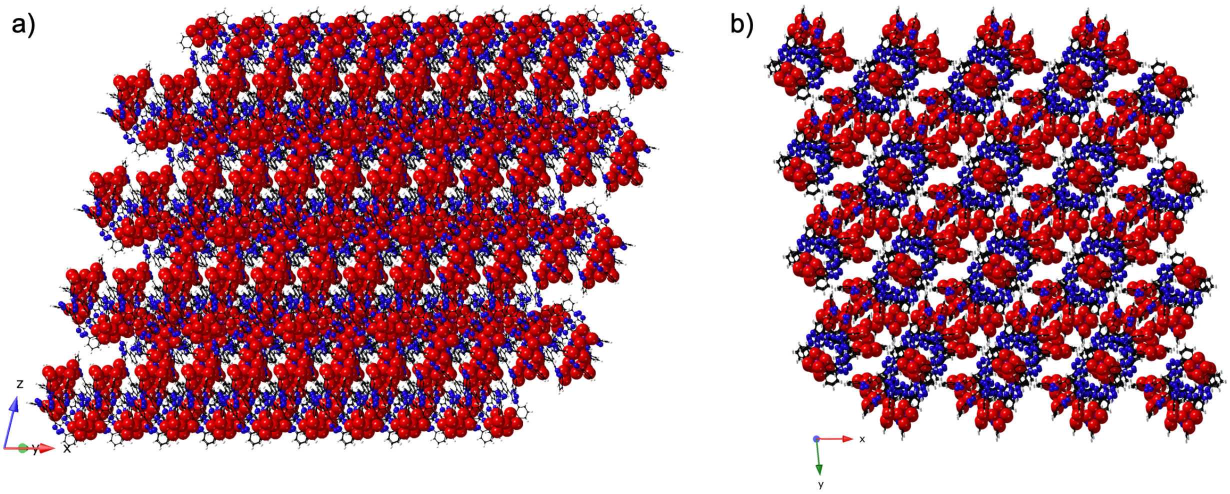

Figure S95. Crystal packing of Ga_6L_4 **IC2**. O atoms shown in red to highlight the catecholate belt and lattice solvent molecules water, methanol and acetone from crystallization. a) View down the crystallographic c-axis and b) view along b. The array of O-rich belts delineates continuous solvent channels where the K^+ atoms are bridging solvent molecules between the triply interlocked cages. Methanol/H_2O/acetone molecules occupy the channels and bridge outer-sphere K^+ ions, the latter being disordered over several positions and omitted for clarity.

S9.4 Cavity-volume and ESP calculations for IC2

The crystal structures of **IC2** (CCDC 2467879) was analysed with CageCavityCalc (C3).[8] A grid size of 0.70 Å and the EEM charge model were used; the probe radius was set to 1.0 Å (default). The largest contiguous internal voids were 83 Å^3 (Cage I) and 107 Å^3 (Cage II). ESP maps were generated directly from the same grid and are contoured in terms of the residual electron density.

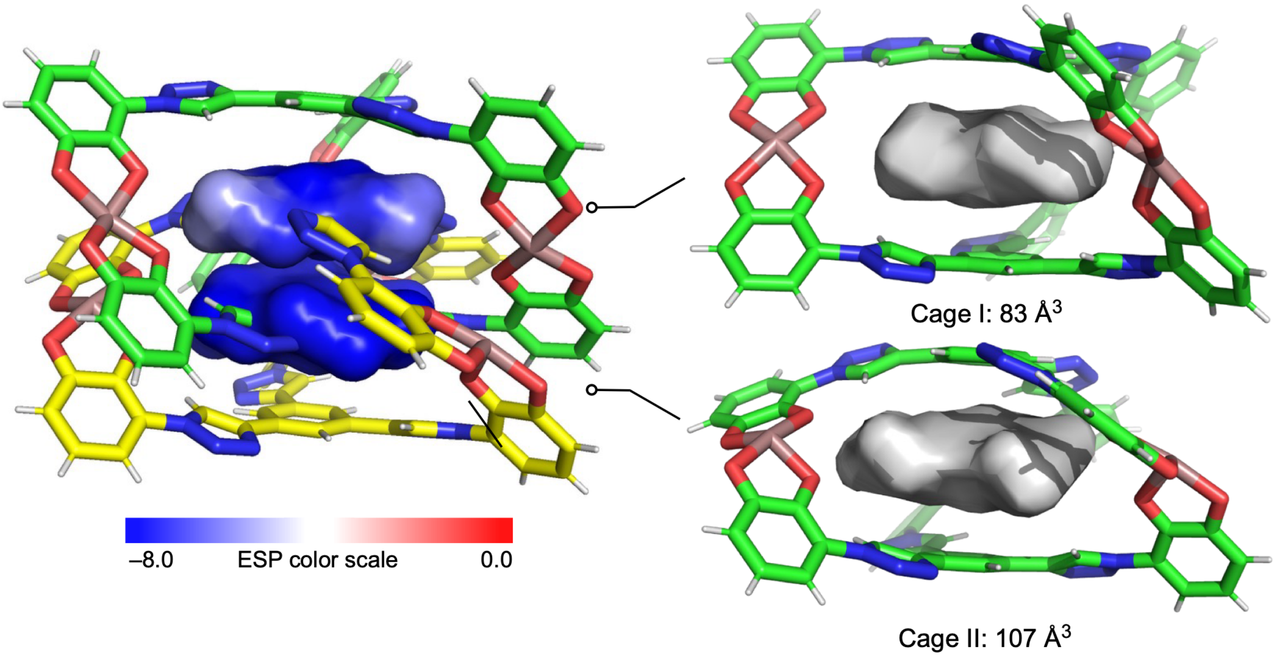

Figure S96. Cavity volume of Cage I and Cage II of Ga_6L_4 **IC2**. The cavity volume is shown as a light blue ESP surface. Calculation of the cavity volume was performed using CageCavityCalc(C3) (probe radius 1Å, grid 0.7 Å).

S10 Computational details

Calculations were performed using Gaussian16 software,[9] using B3LYP functional[10-12] combined with the Grimme's D3 correction for dispersion.[13] Structures were optimized employing basis set 6-31g(d) for all the atoms.[14, 13] The Non Covalent Interaction analysis (NCI) was performed based on a 2D plot of the reduced density gradient, *s*, and the electron density, *ρ*.[15, 16] The method bases its estimations on computing the reduced gradient of the electron density (*s*) versus the electron density (*ρ*) multiplied by the sign of the second Hessian eigenvalue (λ_2). Strong stabilizing interactions typically correspond to values of $\rho > 0.01$ a.u. and $\lambda_2 < 0$, whereas strong destabilizing interactions are associated with values of $\rho > 0.01$ a.u. and $\lambda_2 > 0$. For delocalized weak interactions both density and gradient are small (typically $\rho < 0.01$ a.u. and $\lambda_2 \sim 0$). The Non-Covalent Interaction Analysis (NCI) reported in this work was carried out with NCIPlot. NCI were first computed employing the DFT electron densities of the optimized structures; for clarity, the representation of the isosurfaces were constructed at promolecular level reporting the DFT density map. The computational data obtained in this study is available in the CORA repository and can be accessed openly at: https://doi.org/10.34810/data2420.

S10.1 NCI Analyses of IC1 and IC2

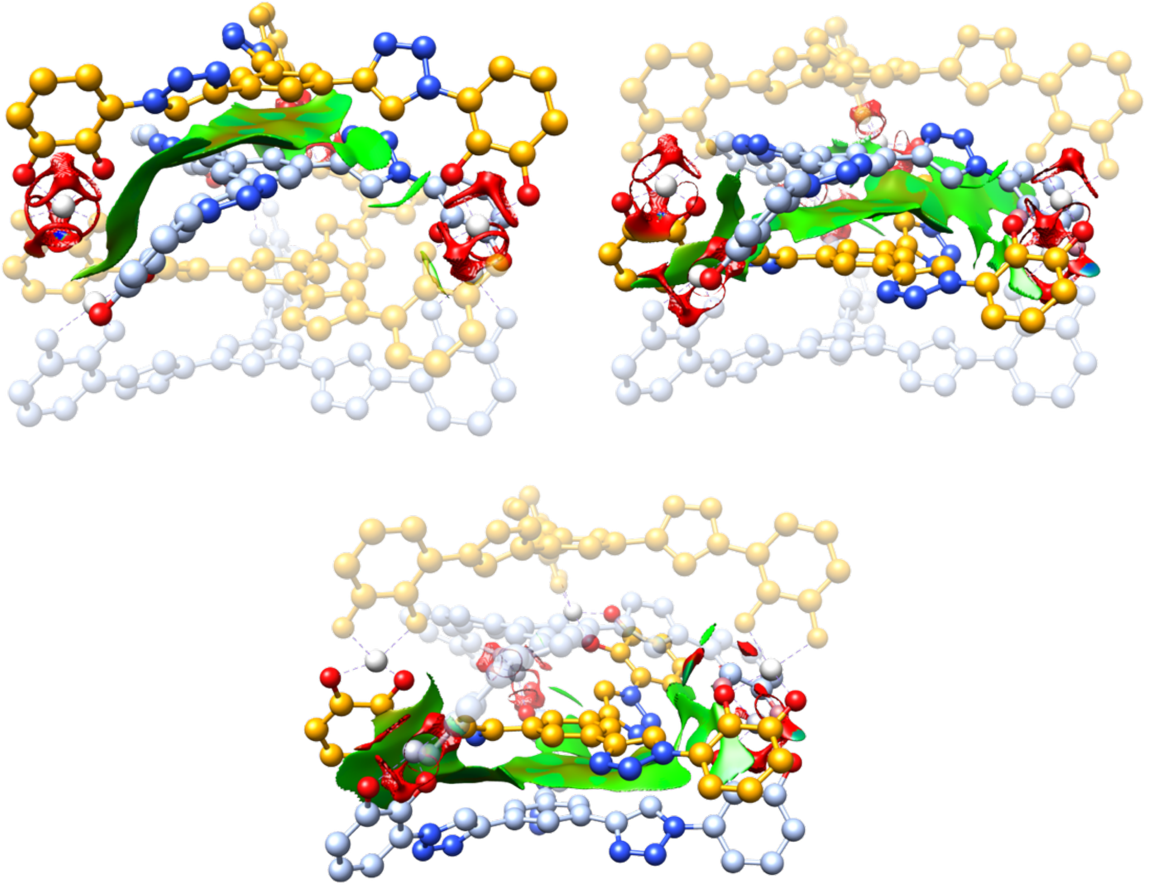

Figure S97. Non-covalent interactions (NCI) plots for **IC1**.

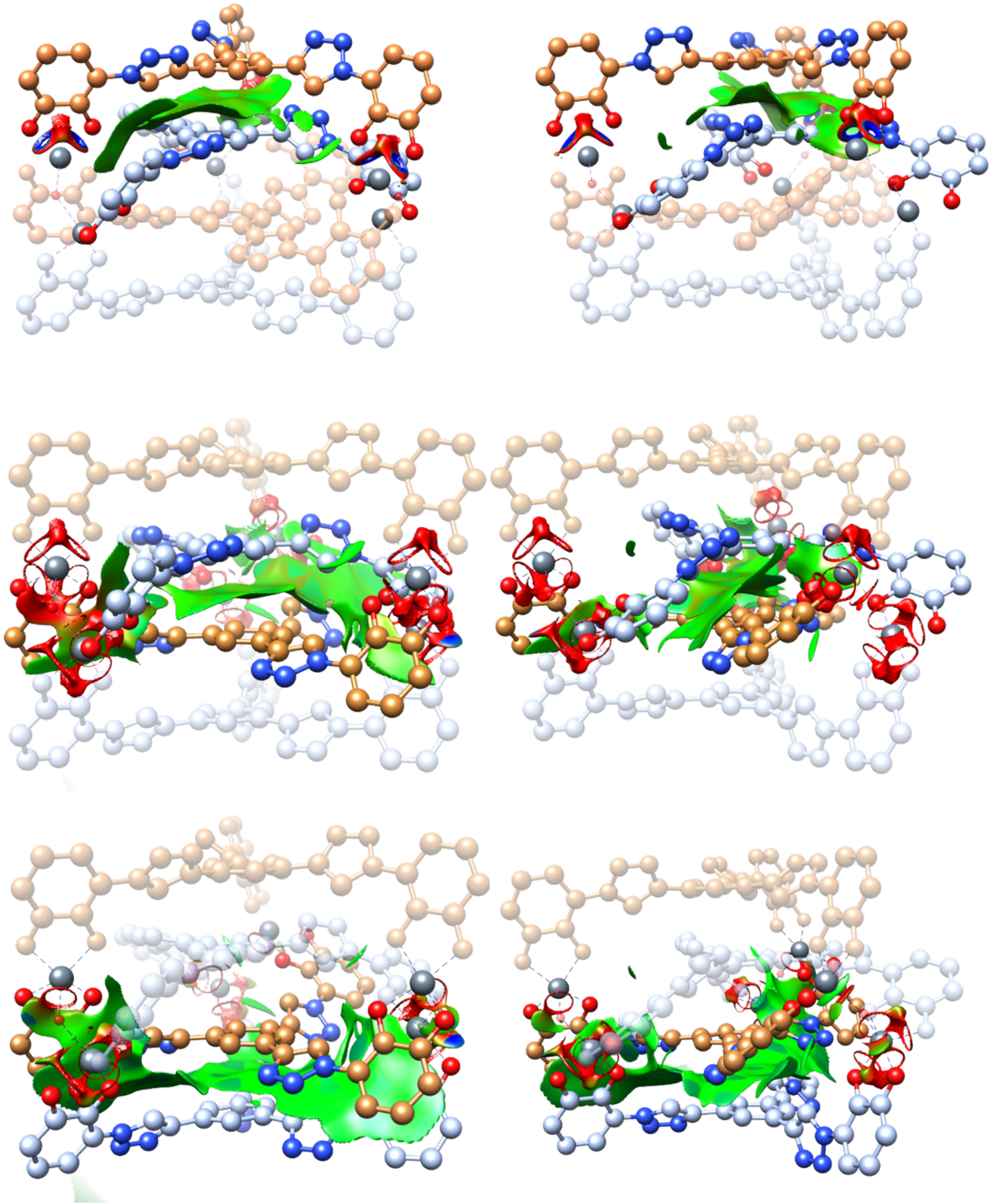

Figure S98. Non-covalent interactions (NCI) plots for **IC2**.

S10.2 Cartesian coordinates of IC1 and IC2

IC1

Al	13.2581	12.2531	23.8705
Al	10.8065	23.5677	19.0541
Al	16.7049	12.6256	24.1207
Al	19.3017	14.4397	12.0979
Al	10.2177	21.9558	15.9693
Al	19.5224	17.9257	12.7135
O	17.4580	14.0901	12.0883
O	13.5013	14.0098	23.3381
O	12.9219	11.7414	22.0904
O	12.0410	21.5009	16.0461
O	10.6623	21.7438	19.4071
O	17.7158	11.1358	23.7255
O	19.2504	13.8464	10.3010
O	17.1255	12.5576	25.9761
O	16.3640	12.8040	22.3021
O	12.6673	12.9253	25.5151
O	19.3545	14.4070	13.9549
O	17.1226	14.4436	24.2610
O	12.4946	10.5853	24.3163
O	10.6110	22.9177	14.4447
O	19.1432	19.2559	11.5068
O	12.6090	23.6535	19.5054
O	9.7255	20.1628	16.1545
O	15.0047	11.9294	24.4672
O	21.0495	13.7513	12.1582
O	17.6980	17.8603	13.1071
O	8.3594	22.2735	15.7381
O	10.9590	25.4494	18.7173
O	9.4009	23.8139	20.2317
O	10.3365	23.3070	17.2844
O	19.6656	16.2513	11.8562
O	20.0122	18.1693	14.4998
N	14.6170	13.4705	11.5273
O	21.4028	18.0952	12.3492
N	9.9737	19.0545	20.2573
N	15.6620	12.2458	19.5376
N	20.0974	14.7115	16.7347
N	14.5152	16.7225	23.2665
N	12.2551	10.4308	19.5146
N	8.4690	17.4850	16.1009
N	14.8016	18.3823	13.3984
N	12.9185	17.9876	14.3649
N	9.8456	17.8802	20.9507
N	13.4500	18.5450	13.3116
N	15.4010	24.2809	20.1190
N	19.2851	14.9413	18.7131
N	10.6570	17.0154	20.4087
N	20.1952	14.2997	18.0347
N	14.2575	18.0523	23.0971
N	15.9038	12.1608	18.1960
N	13.3333	13.4369	11.0686
N	8.6019	15.4117	16.6710
N	14.9580	21.2774	15.7124
N	14.7118	18.3884	21.9269
N	17.8085	17.2204	25.0075
N	15.3313	13.1794	17.6320
C	13.9977	15.1775	18.1843
N	21.2688	18.8103	17.1120
N	12.3775	10.3992	17.3626
N	12.4085	9.6321	18.4193
N	7.7989	16.4407	16.6697
N	12.5390	13.5346	12.0997
N	16.8445	20.4597	16.3495
C	13.0176	15.7813	18.9892
H	12.7866	15.3790	19.9716
N	16.3099	21.3465	15.5550
C	18.5869	15.7744	17.8763
C	12.3356	16.9235	18.5402
C	14.2954	15.7325	16.9298
N	21.1784	18.6766	19.2595
C	10.8807	14.7708	15.9375
C	15.8965	17.4104	19.9879
C	11.0953	13.7915	16.9205
H	10.4953	13.8179	17.8246
C	12.6362	17.4484	17.2749
H	12.1005	18.3174	16.9063
C	11.6407	14.7261	14.7612
H	11.4898	15.4777	13.9934
C	10.8851	18.9098	19.2586
H	11.1363	19.7207	18.5981
C	11.3239	17.6008	19.3642
C	12.6042	13.7253	14.5622
C	15.5399	18.4852	19.1585
H	14.7891	19.1884	19.5016
C	13.2843	13.6337	13.2478
N	17.1751	23.6641	21.1767
C	16.1694	18.6680	17.9235
C	17.5179	16.6752	18.3253
C	9.2486	20.2134	20.6542
N	16.4152	24.6989	20.9288
C	13.6146	16.8597	16.4650
C	16.2717	11.2946	20.4028
N	17.8156	19.3210	24.5198
C	14.4436	25.2354	19.6502
N	17.7567	18.4844	25.5201
C	9.8015	15.7636	16.1076
C	9.6470	21.4879	20.2176
C	12.0540	12.7842	16.7442
C	15.8574	19.8050	17.0418
C	13.5868	14.7762	24.4230
C	14.9151	13.3523	19.8146
H	14.5926	13.5799	20.8171

N	21.9504	18.4422	18.2363
C	17.1489	17.7562	17.5128
H	17.6452	17.9148	16.5610
C	14.7100	13.9539	18.5817
C	17.8153	16.1037	25.9038
C	15.2711	17.2949	21.3123
C	14.6238	13.5984	12.8876
C	12.7180	22.1141	15.0829
C	12.1055	9.8337	20.8074
C	20.9930	14.2353	15.7395
C	15.1458	16.2151	22.1731
H	15.4597	15.1846	22.1272
C	18.1897	16.3392	27.2490
H	18.4485	17.3516	27.5357
C	12.1318	11.7319	19.1297
H	12.0526	12.5306	19.8488
C	15.6904	13.3349	10.5892
C	13.9058	17.4487	15.1491
C	18.1949	20.7700	19.6356
H	18.2634	21.3172	18.7001
C	7.6630	21.1558	15.6973
C	17.0314	18.7642	12.3911
C	14.1005	16.0774	24.4627
C	13.1255	14.1794	25.6414
C	18.0337	13.5225	9.9037
C	15.1184	17.6831	14.5232
H	16.1389	17.4630	14.7818
C	13.7241	16.1940	26.8521
H	13.8113	16.7364	27.7939
C	17.8321	19.5367	11.4843
C	9.7241	17.0999	15.7381
H	10.4415	17.7705	15.2866
C	14.1061	22.0525	14.8672
C	14.6913	22.7352	13.7788
H	15.7533	22.6159	13.6105
C	21.5243	13.7479	13.4073
C	16.6360	11.6469	21.7085
C	17.0151	13.6650	10.9163
C	12.8115	12.7646	15.5609
C	18.9617	19.6074	19.8150
C	17.3576	21.2419	20.6580
C	22.8196	13.3839	13.7756
H	23.5200	13.0794	12.9971
C	8.9309	22.6406	20.6819
C	20.5742	14.1428	14.4045
C	14.1813	16.7821	25.6797
H	14.6218	17.7727	25.6709
C	19.1058	15.6287	16.6014
H	18.8771	16.0846	15.6531
C	7.7783	18.7194	15.8875
C	16.8851	16.5081	19.5646
H	17.1759	15.6754	20.1981
C	15.3769	12.8536	9.2929
H	14.3435	12.6115	9.0768
C	8.4323	19.9578	15.9337

C	15.6562	19.0459	12.4654
C	15.5266	22.9476	19.8593
H	14.8119	22.4119	19.2544
C	12.2048	11.7057	17.7435
C	12.4342	10.5151	21.9901
C	8.1402	20.0784	21.5169
H	7.8605	19.0806	21.8342
C	12.1993	9.8570	23.2523
C	14.6417	20.3388	16.6493
H	13.6203	20.1154	16.8999
C	13.1883	14.8918	26.8385
H	12.8441	14.4077	27.7530
C	16.5803	10.0151	19.8972
H	16.2662	9.7746	18.8885
C	17.2669	20.5230	21.8558
H	16.6119	20.8709	22.6461
C	13.1172	24.8696	19.3819
C	16.6708	22.5579	20.5376
C	11.9270	22.9036	14.1807
C	17.4726	13.7421	26.4560
C	11.5794	8.5199	20.8637
H	11.3464	8.0263	19.9271
C	17.3811	10.7076	22.4965
C	14.8623	26.5773	19.4880
H	15.8985	26.8184	19.6978
C	19.9834	19.1966	18.8295
C	21.3045	18.4370	14.6535
C	20.0345	19.2848	17.4436
H	19.3113	19.6134	16.7107
C	17.4655	14.8103	25.4880
C	17.7059	13.0542	8.6299
H	18.5004	12.9566	7.8899
C	15.0874	20.0465	11.6471
H	14.0368	20.2734	11.7712
C	16.3695	12.7206	8.3330
H	16.1078	12.3519	7.3380
C	22.3080	13.8785	16.1012
H	22.5914	13.9684	17.1438
C	17.9027	18.6276	23.3395
C	17.9963	19.3380	22.0459
C	7.8426	22.4942	21.5394
H	7.3140	23.3872	21.8746
C	23.2032	13.4441	15.1306
H	24.2218	13.1775	15.4191
C	21.9517	18.7613	15.8544
C	17.6979	9.4534	21.9810
H	18.2553	8.7562	22.6072
C	13.9573	27.5444	19.0728
H	14.2937	28.5757	18.9429
C	18.8425	18.8945	21.0207
H	19.4334	17.9939	21.1533
C	18.1967	15.2994	28.1676
H	18.4830	15.4999	29.2030
C	6.2899	21.0724	15.4486
H	5.7356	21.9959	15.2817

C	11.6853	8.5596	23.2896
H	11.5252	8.0906	24.2605
C	7.4514	21.2043	21.9526
H	6.5952	21.0842	22.6195
C	13.9077	23.4977	12.9260
H	14.3754	23.9993	12.0783
C	17.8975	17.2732	23.6477
H	17.8963	16.3793	23.0467
C	17.2572	20.5030	10.6647
H	17.9035	21.0673	9.9922
C	22.0856	18.3953	13.4409
C	12.1899	25.8818	18.9382
C	11.3822	7.8929	22.0857
H	10.9799	6.8772	22.1111
C	15.8754	20.7536	10.7516
H	15.4203	21.5388	10.1476
C	23.3393	19.0441	15.8658
H	23.8006	19.2812	16.8180
C	12.5171	23.5898	13.1244
H	11.8788	24.1727	12.4599
C	6.3878	18.6524	15.6249
H	5.9205	17.6740	15.6065
C	17.2856	9.1094	20.6786
H	17.5147	8.1205	20.2788
C	17.8407	13.9933	27.7787
H	17.8365	13.1653	28.4876
C	12.6171	27.2045	18.7966
H	11.8971	27.9508	18.4607
C	24.0776	18.9912	14.6930
H	25.1487	19.2053	14.7191
C	5.6580	19.8139	15.4168
H	4.5850	19.7480	15.2200
C	23.4545	18.6687	13.4725
H	24.0136	18.6219	12.5382
H	20.6063	16.2809	11.6027
H	14.9395	11.7859	25.4243
H	10.3319	24.1614	16.8268
H	15.0492	15.2586	16.3119
H	13.5589	11.9907	15.4272
H	15.5430	13.6878	13.4448

IC2

Ga	13.2642	12.1271	23.6761
Ga	10.8193	23.4705	18.8812
Ga	16.8058	12.4725	23.8643
Ga	19.3925	14.2635	11.8388
Ga	10.2043	21.8768	15.6421
Ga	19.4884	17.8434	12.4431
O	17.4771	13.8339	11.8653
O	13.5532	13.9572	23.1452
O	12.8880	11.5960	21.8217
O	12.1040	21.4409	15.7598
O	10.6481	21.5810	19.2565
O	17.7860	10.8880	23.3802
O	19.3373	13.3859	10.0798
O	17.3197	12.4244	25.7709
O	16.3924	12.6552	21.9826
O	12.7876	12.8589	25.4281
O	19.5051	14.3164	13.7637
O	17.2294	14.3709	24.0171
O	12.3149	10.4686	24.1157
O	10.7065	23.0234	14.1734
O	19.0770	19.2816	11.2475
O	12.7209	23.5400	19.2700
O	9.6963	20.0043	15.8435
O	15.0653	11.6920	24.2946
O	21.2996	13.8026	11.9021
O	17.5932	17.8040	12.8665
O	11.0251	25.4117	18.5209
O	9.3703	23.7107	20.1388
O	10.1833	23.2317	17.0640
O	19.6250	16.1519	11.4268
O	20.0333	18.0557	14.3009
N	14.6776	13.1682	11.3296
O	21.4405	17.9838	12.0740
N	9.9186	18.9274	20.0631
N	15.5880	12.1145	19.2751
N	20.2647	14.8611	16.4644
N	14.6296	16.6100	23.0521
N	12.1805	10.3049	19.2917
N	8.4826	17.3420	15.7658
N	14.7551	18.3833	13.2273
N	12.8713	17.9312	14.1660
N	9.8229	17.7590	20.7729
N	13.4015	18.5214	13.1300
N	15.4996	24.1583	19.8271
N	19.4492	15.0241	18.4482
N	10.6350	16.9001	20.2229
N	20.4144	14.4710	17.7665
N	14.3677	17.9398	22.8913
N	15.8306	12.0457	17.9323
N	13.4050	13.2143	10.8416
N	8.6146	15.2816	16.3798
N	15.0093	21.2881	15.5436
N	14.7838	18.2759	21.7073
N	17.9030	17.1142	24.7641
N	15.2759	13.0815	17.3829
C	13.9587	15.0836	17.9576
N	21.3302	18.5885	16.8712
N	12.3545	10.2560	17.1440
N	12.3559	9.4967	18.2072
N	7.8088	16.3078	16.3485
N	12.5965	13.3762	11.8536
N	16.8747	20.4215	16.1790

C	12.9754	15.6761	18.7673
H	12.7451	15.2631	19.7455
N	16.3646	21.3433	15.4109
C	18.6649	15.7761	17.6105
C	12.2868	16.8181	18.3281
C	14.2564	15.6526	16.7089
N	21.2244	18.4505	19.0173
C	10.8981	14.6291	15.6713
C	15.9210	17.3068	19.7376
C	11.0953	13.6554	16.6634
H	10.4841	13.6912	17.5595
C	12.5875	17.3572	17.0686
H	12.0497	18.2275	16.7058
C	11.6704	14.5708	14.5030
H	11.5301	15.3157	13.7265
C	10.8094	18.7830	19.0462
H	11.0339	19.5879	18.3695
C	11.2718	17.4831	19.1582
C	12.6267	13.5599	14.3208
C	15.5449	18.3866	18.9223
H	14.7931	19.0805	19.2819
C	13.3215	13.4394	13.0174
N	17.2617	23.5379	20.9030
C	16.1674	18.5934	17.6874
C	17.5488	16.6165	18.0625
C	9.1979	20.0853	20.4730
N	16.5059	24.5736	20.6491
C	13.5709	16.7821	16.2550
C	16.1790	11.1338	20.1213
N	17.8658	19.2140	24.2735
C	14.5451	25.1177	19.3604
N	17.8209	18.3774	25.2750
C	9.8192	15.6253	15.8208
C	9.6178	21.3617	20.0591
C	12.0476	12.6394	16.5039
C	15.8684	19.7540	16.8316
C	13.7136	14.6847	24.2502
C	14.8604	13.2299	19.5674
H	14.5466	13.4523	20.5740
N	21.9898	18.1867	17.9967
C	17.1573	17.6997	17.2636
H	17.6570	17.8836	16.3185
C	14.6650	13.8520	18.3426
C	17.9452	15.9985	25.6603
C	15.3230	17.1823	21.0744
C	14.6630	13.3140	12.6872
C	12.7921	22.1581	14.8778
C	11.9757	9.7231	20.5837
C	21.2085	14.4966	15.4656
C	15.2236	16.1022	21.9381
H	15.5281	15.0695	21.8776
C	18.3524	16.2405	26.9929
H	18.6048	17.2568	27.2720
C	12.0733	11.6034	18.8941
H	11.9795	12.4084	19.6042

C	15.7582	12.9232	10.4227
C	13.8613	17.3943	14.9487
C	18.2816	20.6282	19.3729
H	18.3515	21.1639	18.4309
C	7.6505	20.9830	15.1956
C	16.9701	18.7750	12.1987
C	14.2660	15.9714	24.2694
C	13.3019	14.0955	25.4933
C	18.1086	13.0301	9.7463
C	15.0741	17.6652	14.3393
H	16.0949	17.4558	14.6040
C	14.0355	16.0907	26.6734
H	14.1962	16.6231	27.6108
C	17.7705	19.5732	11.3101
C	9.7430	16.9538	15.4251
H	10.4669	17.6174	14.9721
C	14.1902	22.1275	14.7264
C	14.8245	22.9081	13.7363
H	15.8956	22.8146	13.6193
C	21.7581	13.9716	13.1455
C	16.6107	11.4749	21.4101
C	17.0809	13.2858	10.7317
C	12.8158	12.6051	15.3288
C	19.0402	19.4622	19.5654
C	17.4461	21.1159	20.3895
C	23.1038	13.8592	13.5024
H	23.8286	13.6337	12.7195
C	8.9112	22.5176	20.5403
C	20.7775	14.2682	14.1506
C	14.4394	16.6711	25.4780
H	14.9106	17.6468	25.4457
C	19.1831	15.6674	16.3324
H	18.9063	16.0918	15.3832
C	7.7836	18.5588	15.4866
C	16.9201	16.4224	19.2996
H	17.2325	15.5934	19.9277
C	15.4497	12.3020	9.1882
H	14.4163	12.0389	8.9956
C	8.4214	19.8075	15.5363
C	15.6049	19.0898	12.3206
C	15.6272	22.8255	19.5664
H	14.9198	22.2905	18.9524
C	12.1781	11.5664	17.5111
C	12.3252	10.4015	21.7637
C	8.0844	19.9422	21.3260
H	7.7949	18.9411	21.6237
C	12.0167	9.7742	23.0291
C	14.6656	20.3159	16.4383
H	13.6375	20.0918	16.6604
C	13.4622	14.8064	26.6841
H	13.1573	14.3278	27.6152
C	16.4060	9.8439	19.6023
H	16.0336	9.6180	18.6101
C	17.3500	20.4114	21.5957
H	16.6964	20.7720	22.3814

C	13.2079	24.7611	19.1239
C	16.7637	22.4331	20.2564
C	12.0354	23.0188	14.0070
C	17.6540	13.6296	26.2102
C	11.3714	8.4452	20.6354
H	11.1275	7.9603	19.6967
C	17.3537	10.5049	22.1681
C	14.9806	26.4505	19.1817
H	16.0251	26.6759	19.3664
C	20.0545	19.0235	18.5849
C	21.3415	18.2548	14.4120
C	20.1167	19.1159	17.2003
H	19.4110	19.4719	16.4642
C	17.5985	14.7023	25.2450
C	17.7815	12.4245	8.5307
H	18.5826	12.2442	7.8136
C	15.0406	20.1553	11.5879
H	14.0017	20.4047	11.7595
C	16.4486	12.0613	8.2566
H	16.1973	11.5824	7.3071
C	22.5705	14.4017	15.8083
H	22.8551	14.6200	16.8314
C	17.9741	18.5210	23.0949
C	18.0711	19.2236	21.7982
C	7.8164	22.3580	21.3902
H	7.2962	23.2505	21.7393
C	23.5049	14.0716	14.8345
H	24.5622	14.0152	15.0987
C	22.0140	18.5199	15.6148
C	17.5915	9.2397	21.6329
H	18.1480	8.5206	22.2345
C	14.0799	27.4295	18.7872
H	14.4256	28.4554	18.6418
C	18.9141	18.7630	20.7783
H	19.4970	17.8583	20.9189
C	18.3999	15.1997	27.9088
H	18.7109	15.4000	28.9369
C	6.2959	20.8602	14.8698
H	5.7433	21.7680	14.6277
C	11.4251	8.5091	23.0588

H	11.2131	8.0635	24.0309
C	7.4061	21.0675	21.7783
H	6.5450	20.9473	22.4387
C	14.0763	23.7344	12.9126
H	14.5794	24.3179	12.1410
C	17.9956	17.1677	23.4049
H	18.0161	16.2743	22.8038
C	17.1928	20.6027	10.5701
H	17.8366	21.1878	9.9132
C	22.1139	18.2089	13.1921
C	12.2814	25.7900	18.7092
C	11.1098	7.8442	21.8581
H	10.6463	6.8552	21.8871
C	15.8251	20.8925	10.7142
H	15.3812	21.7316	10.1790
C	23.4126	18.7289	15.6310
H	23.8873	18.9170	16.5874
C	12.6779	23.7958	13.0460
H	12.0673	24.4303	12.4034
C	6.4142	18.4550	15.1445
H	5.9674	17.4670	15.1309
C	17.1058	8.9095	20.3539
H	17.2743	7.9105	19.9491
C	18.0542	13.8912	27.5223
H	18.0834	13.0605	28.2274
C	12.7296	27.1042	18.5523
H	12.0092	27.8585	18.2359
C	24.1421	18.6652	14.4535
H	25.2237	18.8168	14.4778
C	5.6805	19.5946	14.8501
H	4.6222	19.5060	14.5923
C	23.4955	18.4096	13.2312
H	24.0471	18.3558	12.2930
H	20.5809	16.1975	11.2335
H	14.9860	11.7209	25.2624
H	10.3137	24.0760	16.6025
H	15.0120	15.1877	16.0864
H	13.5553	11.8219	15.2062
H	15.5720	13.3533	13.2663

S11 References

1 M. S. Khan, M. R. A. Al-Mandhary, M. K. Al-Suti, T. C. Corcoran, Y. Al-Mahrooqi, J. P. Attfield, N. Feeder, W. I. F. David, K. Shankland, R. H. Friend, A. Köhler, E. A. Marseglia, E. Tedesco, C. C. Tang, P. R. Raithby, J. C. Collings, K. P. Roscoe, A. S. Batsanov, L. M. Stimson and T. B. Marder, *New J. Chem.*, 2003, **27**, 140-149.

2 I. Mohammed, I. R. Kummetha, G. Singh, N. Sharova, G. Lichinchi, J. Dang, M. Stevenson and T. M. Rana, *J. Med. Chem.*, 2016, **59**, 7677-7682.

3 W. M. Haynes, *CRC Handbook of Chemistry and Physics*, CRC Press, 2014.

4 S. Alvarez, *Dalton Trans.*, 2013, **42**, 8617-8636.

5 G. M. Sheldrick, *Acta Crystallogr., Sect. A*, 2015, **71**, 3-8.

6 G. M. Sheldrick, *Acta Crystallogr., Sect. A*, 2008, **64**, 112-122.

7 A. Spek, *Acta Crystallographica Section C*, 2015, **71**, 9-18.

8 V. Martí-Centelles, T. K. Piskorz and F. Duarte, *J. Chem. Inf. Model.*, 2024, **64**, 5604-5616.

9 Gaussian 16 Rev. B.01, M. J. Frisch, G. W. Trucks, H. B. Schlegel, G. E. Scuseria, M. A. Robb, J. R. Cheeseman, G. Scalmani, V. Barone, G. A. Petersson, H. Nakatsuji, X. Li, M. Caricato, A. V. Marenich, J. Bloino, B. G. Janesko, R. Gomperts, B. Mennucci, H. P. Hratchian, J. V. Ortiz, A. F. Izmaylov, J. L. Sonnenberg, Williams, F. Ding, F. Lipparini, F. Egidi, J. Goings, B. Peng, A. Petrone, T. Henderson, D. Ranasinghe, V. G. Zakrzewski, J. Gao, N. Rega, G. Zheng, W. Liang, M. Hada, M. Ehara, K. Toyota, R. Fukuda, J. Hasegawa, M. Ishida, T. Nakajima, Y. Honda, O. Kitao, H. Nakai, T. Vreven, K. Throssell, J. A. Montgomery Jr., J. E. Peralta, F. Ogliaro, M. J. Bearpark, J. J. Heyd, E. N. Brothers, K. N. Kudin, V. N. Staroverov, T. A. Keith, R. Kobayashi, J. Normand, K. Raghavachari, A. P. Rendell, J. C. Burant, S. S. Iyengar, J. Tomasi, M. Cossi, J. M. Millam, M. Klene, C. Adamo, R. Cammi, J. W. Ochterski, R. L. Martin, K. Morokuma, O. Farkas, J. B. Foresman and D. J. Fox, Gaussian 16, Wallingford, CT, 2016.

10 C. Lee, W. Yang and R. G. Parr, *Phys. Rev. B.*, 1988, **37**, 785-789.

11 B. Miehlich, A. Savin, H. Stoll and H. Preuss, *Chem. Phys. Lett.*, 1989, **157**, 200-206.

12 A. D. Becke, *J. Chem. Phys.*, 1993, **98**, 5648-5652.

13 S. Grimme, J. Antony, S. Ehrlich and H. Krieg, *J. Chem. Phys.*, 2010, **132**, 154104.

14 M. M. Francl, W. J. Pietro, W. J. Hehre, J. S. Binkley, M. S. Gordon, D. J. DeFrees and J. A. Pople, *J. Chem. Phys.*, 1982, **77**, 3654-3665.

15 E. R. Johnson, S. Keinan, P. Mori-Sánchez, J. Contreras-García, A. J. Cohen and W. Yang, *J. Am. Chem. Soc.*, 2010, **132**, 6498-6506.

16 J. Contreras-García, E. R. Johnson, S. Keinan, R. Chaudret, J.-P. Piquemal, D. N. Beratan and W. Yang, *J. Chem. Theory Comput.*, 2011, **7**, 625-632.
